# Linear Response Function of Bond-Order

**DOI:** 10.3390/ijms17111779

**Published:** 2016-10-25

**Authors:** Nayuta Suzuki, Yuki Mitsuta, Mitsutaka Okumura, Shusuke Yamanaka

**Affiliations:** Graduate School of Science, Osaka University, Machikaneyama 1-1, Toyoanaka, Osaka 560-0043, Japan; suzukin15@chem.sci.osaka-u.ac.jp (N.S.); mitsutay13@chem.sci.osaka-u.ac.jp (Y.M.); ok@chem.sci.osaka-u.ac.jp (M.O.)

**Keywords:** linear response function, bond order, acid dissociation reaction

## Abstract

We present the linear response function of bond-orders (LRF-BO) based on a real space integration scheme for molecular systems. As in the case of the LRF of density, the LRF-BO is defined as the response of the bond order of the molecule for the virtual perturbation. Our calculations show that the LRF-BO enables us not only to detect inductive and resonating effects of conjugating systems, but also to predict p*K*_a_ values on substitution groups via linear relationships between the Hammett constants and the LRF-BO values for meta- and para-substituted benzoic acids. More importantly, the LRF-BO values for the O-H bonds strongly depend on the sites to which the virtual perturbation is applied, implying that the LRF-BO values include essential information about reaction mechanism of the acid-dissociation of substituted benzoic acids.

## 1. Introduction

During the past decades, the main branch of contemporary computational chemistry has been developed mainly to simulate chemical phenomena realistically, typically with quantum mechanics/molecular mechanics (QM/MM) methods [1,2]. In the QM/MM methods, it is required to cover environmental effects such as solvent effects for chemical reactions in liquid states and effects of surrounding proteins and lipids for enzyme reactions. As a result, this type of approach involves huge computational costs to achieve reliable simulations. In contrast, another branch of computational chemistry has also grown steadily, which involves a strategy to inspect intrinsic properties of molecules that are related to functionality and chemical reactivity in order to comprehend and predict chemical phenomena. One of the well-established properties is the Fukui functions, which are defined as the left (−) and right (+) side derivatives of density with respect to the number of electrons, N, i.e., $f^{\pm }(r)\equiv {\left(\partial \rho (r)/\partial N\right)}_{v}^{\pm }$, and are known to be typical indicators for nucleophilic and electrophilic attacking sites, respectively [3,4,5]. Also, a derivative of density with respect to external potential (v) with fixing the number of electrons (N), ${\left(\partial \rho (r)/\partial v(r^{\prime })\right)}_{N}$, called the linear response function (LRF), has been found to be a measure of electron delocalization properties such as induced and resonating effects of conjugated systems [4,5,6,7,8,9,10,11,12,13,14,15,16]. Also, the LRF is reported to have maximum values at transition states for several Diels-Alder reactions, being another important descriptor of chemical reactions [13]. These two properties, together with other properties, such as softness and hardness that have been well-known concepts in general chemistry, are defined as energy derivatives or grandcanonical potential (at T = 0 K) derivatives with v, N, and chemical potential, μ, and are related to each other [3,5,14], enabling us to understand intrinsic properties of molecules in a quantitative way using ab initio computational results. This type of theory is now well established and called “conceptual density functional theory (DFT)”. 

Since the purpose of the second branch of computational chemistry is to understand and to predict chemical phenomena via inspecting intrinsic properties of molecules as descriptors of chemical reactivities without implementing realistic simulations like QM/MM molecular dynamics approaches, it is also possible to consider other reactivity descriptors that are not based on density functional theory. Considering that the change of bond-orders is a more direct property that describes the essence of chemical reactions, it is valuable to try to see linear response functions of the bond-orders (LRF-BO) of molecules. However, to our best knowledge, this property, the LRF-BO, has not been investigated in ab initio quantum chemistry. It should be noted that Coulson and Longuet-Higgins [17,18], and Fukui et al. [19] formulated linear response functions of off-diagonal parts of density matrices (DM) within the Hückel model of conjugated organic molecules more than 50 years ago, but they investigated perturbation energies rather than the LRF of DM itself as a descriptor of chemical reactivities of molecules. Our purpose is to inspect the sensitivity of a specific chemical bond-order, on which we focus our attention, for virtual perturbations. 

For this purpose, we presented the LRF-BO using DFT computational results in a previous study [20]. As a continuation of the previous work, but with essential improvements for avoiding ambiguity of the numerical treatments in our previous definition, we explore the possibility of the LRF-BO as a useful descriptor of chemical reactivities of molecules. We first show the LRF-BO values of water molecule and those interpretations as a typical example of the use of the LRF-BO. Then, we examine the LRF-BO calculations of conjugated and non-conjugated organic molecules, to show that the LRF-BO can be used as a complement to the LRF of density. Further, we present the relationship between the Hammett constants and the LRF-BO values for the meta- and para-substituted benzoic acids. In addition, we inspect dependency of $\delta B^{\mathrm{OH}}/\delta v(K)$ values on K, i.e., the site to which we apply the perturbation, and discuss the implication of the LRF-BO values in describing chemical reaction mechanism of the acid dissociations. 

## 2. Linear Response Function of Bond-Order

Let us derive the linear response function (LRF) of the bond-order (BO). For simplicity, we assume that the target molecules are closed-shell systems, but the generalization of the following formulation to the open-shell systems is straightforward. 

We start from an expression of the LRF of density matrix in real space, which can be obtained by using the first-order perturbation theory as

(1) $$\frac{\delta \rho (r_{1},r_{2})}{\delta v(r)}=\sum_{\sigma }^{\alpha ,\beta }\sum_{i}^{\mathrm{Occ}}\sum_{j}^{\mathrm{Unocc}}\frac{\psi_{j}^{\sigma }(r)\psi_{i}^{\sigma }(r)}{\varepsilon_{i}^{\sigma }-\varepsilon_{j}^{\sigma }}(\psi_{j}^{\sigma }(r_{1})\psi_{i}^{\sigma }(r_{2})+\psi_{i}^{\sigma }(r_{1})\psi_{j}^{\sigma }(r_{2})).$$

Here the unperturbed state is supposed to be described as a single-determinant wave function consisting of Hartree-Fock or Kohn-Sham orbitals, $\left\{\psi_{k}^{\sigma }\right\}$. The summations run over the spin variables (σ), occupied orbitals (i) and unoccupied orbitals (j). Expanding both sides in terms of atomic orbitals with molecular orbital coefficients, $\left\{C_{k\mu }^{\sigma }\right\}$,

(2) $$\sum_{\mu \nu }\frac{\delta P_{\mu \nu }}{\delta v(r^{\prime })}\phi_{\mu }(r_{1})\phi_{\nu }(r_{2})=\sum_{\sigma }^{\alpha ,\beta }\sum_{ij}\frac{\psi_{j}^{\sigma }(r^{\prime })\psi_{i}^{\sigma }(r^{\prime })}{\varepsilon_{i}^{\sigma }-\varepsilon_{j}^{\sigma }}\sum_{\mu \nu }\left(C_{j\mu }^{\sigma }C_{i\nu }^{\sigma }+C_{i\mu }^{\sigma }C_{j\nu }^{\sigma })\phi_{\mu }(r_{1})\phi_{\nu }(r_{2}\right),$$

and extracting the expansion coefficient, we have

(3) $$\frac{\delta P_{\mu \nu }}{\delta v(r)}=\sum_{\sigma }^{\alpha ,\beta }\sum_{ij}\frac{\psi_{j}^{\sigma }(r)\psi_{i}^{\sigma }(r)}{\varepsilon_{i}^{\sigma }-\varepsilon_{j}^{\sigma }}(C_{j\mu }^{\sigma }C_{i\nu }^{\sigma }+C_{i\mu }^{\sigma }C_{j\nu }^{\sigma }).$$

Note that we neglect the change of AOs due to the perturbation in order to derive Equation (3): this is because we assume that the perturbation arises from reactants, catalysts, solvents, and other environments, not from the changes of the nuclear configuration of the target molecule. 

Now we move on to the definition of linear response function of bond-order. As for the bond-order, we employ the Mayer bond order [21], the most standard definition of the bond-order in the field of ab initio quantum chemistry, which is defined as,

(4) $$B^{\mathrm{IJ}}\equiv \sum_{\mu }^{I}\sum_{\nu }^{J}Q_{\mu \nu }Q_{\nu \mu }.$$

The two summations of basis sets’ indices, μ and ν, run over the I-th atom and the J-th atom, respectively and the matrix, **Q,** is the product of the density matrix (**P**) and the overlap matrix (**S**),

(5) $$Q_{\mu \nu }\equiv \sum_{\eta }P_{\mu \eta }S_{\eta \nu }.$$

From Equation (3), we have an expression for the linear response function of the **Q** matrix as

(6) $$\frac{\delta Q_{\mu \nu }}{\delta v(r)}=\sum_{\sigma }^{\alpha ,\beta }\sum_{ij}\frac{\psi_{j}^{\sigma }(r)\psi_{i}^{\sigma }(r)}{\varepsilon_{i}^{\sigma }-\varepsilon_{j}^{\sigma }}\sum_{\eta }\left(C_{j\mu }^{\sigma }C_{i\eta }^{\sigma }+C_{i\mu }^{\sigma }C_{j\eta }^{\sigma }\right)S_{\eta \nu }.$$

Finally, the linear response function of the bond-order can then be defined as

(7) $$\delta B^{\mathrm{IJ}}/\delta v(r)\equiv \sum_{\mu }^{I}\sum_{\nu }^{J}\left(\frac{\delta Q_{\mu \nu }}{\delta v(r)}Q_{\nu \mu }+Q_{\mu \nu }\frac{\delta Q_{\nu \mu }}{\delta v(r)}\right).$$

It is convenient to consider perturbations that are applied to atomic sites. For this purpose, we define the LRF-BO for the local perturbation, δv(ξ), that is applied to a specific atomic orbital $\phi_{\xi }$ via the following relation,

(8) $$\sum_{\xi }\frac{\delta B^{\mathrm{IJ}}}{\delta v(\xi )}\equiv \int dr\frac{\delta B^{\mathrm{IJ}}}{\delta v(r)}$$

and we then have an expression of LRF-BO for the perturbation to an L-th atom,

(9) $$\frac{\delta B^{\mathrm{IJ}}}{\delta v(L)}\equiv \sum_{\xi }^{L}\frac{\delta B^{\mathrm{IJ}}}{\delta v(\xi )}.$$

Here, the summation, $\sum_{\xi }$, at the left side of Equation (8) runs over all AOs, and that at the right side of Equation (9), $\sum_{\xi }^{L}$ is limited to the AOs that belong to the L-th atom. This is the scheme we employed in reference [20], which is the first implementation of the LRF-BO. The LRF-BO based on AO perturbations (Equation (8)) suits the linear combination of AOs (LCAO) formalism: for instance, we easily see the LRF-BO for the case that the perturbation is applied to a π orbital at a specific carbon atom in a π-conjugated system. However, when we would like to see the effects due to a nucleophilic or electrophilic attack to a specific atom, it is unclear whether the perturbation, δv(ξ), is attractive or repulsive since atomic orbitals except 1s orbitals have different (positive and negative) phases’ parts in their distributions. To avoid such ambiguity, we here define $\delta B^{\mathrm{IJ}}/\delta v(L)$ using a numerical integration

(10) $$\frac{\delta B^{\mathrm{IJ}}}{\delta v(L)}\equiv \int^{L}dr\frac{\delta B^{\mathrm{IJ}}}{\delta v(r)},$$

where the domain of integration for the L-th atom at the left side is defined as the Wigner-Seitz cell. The Wigner-Seitz cell for molecular systems is constructed in a similar manner to that of solid systems: the region for a specific atom in the molecule can be defined as a region encircled by all perpendicular bisectors with neighboring atoms (See Figure S1).

From the above definition, we can derive some fundamental features of the LRF-BO. First, the integration of the LRF-BO over the whole space always vanishes,

(11) $$\int dr\frac{\delta B^{\mathrm{IJ}}}{\delta v(r)}=\sum_{L}^{\underset{\mathrm{atoms}}{\mathrm{All}}}\frac{\delta B^{\mathrm{IJ}}}{\delta v(L)}=0,$$

because the occupied orbital, $\psi_{i}^{\sigma }$, and the unoccupied orbital, $\psi_{j}^{\sigma }$ in the right side of Equation (6) are orthogonal each other. If we notice that the left side of Equation (11) is the total response of the bond-order for the case that the perturbation is homogeneous for the whole system, we deduce Equation (11) trivially: the homogeneous perturbation is equivalent to addition of a constant term to the original Hamiltonian. Second, the summation over all atomic pairs (I, J) of the LRF-BO also vanishes as,

(12) $$\sum_{I,\;J}^{\underset{\mathrm{atoms}}{\mathrm{All}}}\frac{\delta B^{\mathrm{IJ}}}{\delta v(r)}=\sum_{I,\;J}^{\underset{\mathrm{atoms}}{\mathrm{All}}}\frac{\delta B^{\mathrm{IJ}}}{\delta v(L)}=0.$$

We should note that the summation includes I=J, so that this equation does not mean that the sum of the bond-orders is conserved. Equations (11) and (12) are the results from the fact that the LRF-BO in this paper is defined for constant N, which is parallel to a known relation for the LRF of density by fixing the number of electrons, $\int dr\delta \rho (r)/\delta v(r^{\prime })=\int dr^{\prime }\delta \rho (r)/\delta v(r^{\prime })=0$.

As for computational details, three dimensional integration in Equation (10) is approximated in a manner similar to that used in usual DFT computations [22], namely by employing the combination of the Euler-Mclaurin formula for the radial integration and the Lebedev quadrature for the spherical integration for the L-th center integral [23]:

(13) $$\int^{L}dr\frac{\delta B^{\mathrm{IJ}}}{\delta v(r)}≅\sum_{R_{L}(i)}^{\underset{\mathrm{Points}}{\mathrm{Radial}}}\sum_{S_{L}(j)}^{\underset{\mathrm{Points}}{\mathrm{Spherical}}}{w(R}_{L}{(i))w(S}_{L}(j))\frac{\delta B^{\mathrm{IJ}}}{\delta v(R_{L}{(i),S}_{L}(j))}.$$

Note that, for the integration over the L-th center integral, we do not use the Becke’s fuzzy cell [22], which is usually used in the DFT numerical integral scheme, but the Wigner-Seitz cell. The reason why the Becke’s fuzzy cell is not used is that we would like to apply the virtual perturbation to a specific localized region in the space (if the Becke’s fuzzy cell is used, the region defined as the L-th center integral partially spreads over other atomic sites). 

For all molecules we examined below, we used the B3LYP functional [24] and the 6-311G** basis set for exchange correlation functional and basis set, respectively. First, we optimized the geometries and subsequently we calculated the LRF-BO values for the geometries. All calculations were done using a locally modified version of GAMESS [25] unless stated otherwise.

## 3. Computational Results

### 3.1. Water Molecule

We will begin by considering a water molecule to exemplify how to interpret LRF-BO values. 

**Figure 1:**
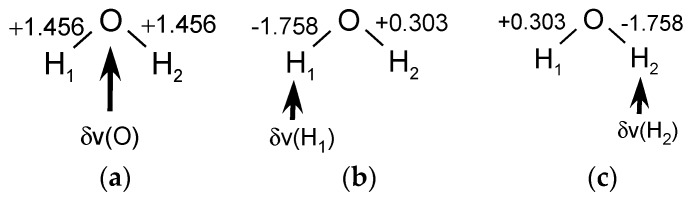
The linear response function of bond-order (LRF-BO) values of a water molecule for perturbations that are applied to the various sites: (**a**) the oxygen atom; (**b**) the left hydrogen atom; and (**c**) the right hydrogen atom.

The computational values of the LRF-BO for the water molecule are summarized in Figure 1. When the virtual perturbation is applied to the oxygen site, the LRF-BO value for both of the OH bonds is 1.456 as shown in Figure 1a. When the perturbation is applied to the left hydrogen site (H_1_), the LRF-BO values of two O-H bonds are different each other, i.e., $\delta B^{{\mathrm{OH}}_{1}}/\delta {v(H}_{1})=-1.758$ and $\delta B^{{\mathrm{OH}}_{2}}/\delta {v(H}_{1})=+0.303$ (Figure 1b). Naturally, the left and right become reverse for the perturbation to the right hydrogen (H_2_), i.e., $\delta B^{{\mathrm{OH}}_{2}}/\delta {v(H}_{2})=-1.758$ and $\delta B^{{\mathrm{OH}}_{1}}/\delta {v(H}_{2})=0.303$, which are shown in Figure 1c.

A positive value of $\delta B^{\mathrm{IJ}}/\delta v(X)$ indicates that if the virtual perturbation (δv(X)) is repulsive for electrons, the bond order increases, and that if the virtual perturbation is attractive for electrons, the bond-order decreases. This is because the following relation holds:

(14) $$\Delta B^{\mathrm{IJ}}≅\frac{\delta B^{\mathrm{IJ}}}{\delta v(X)}\delta v(X).$$

**Figure 2:**
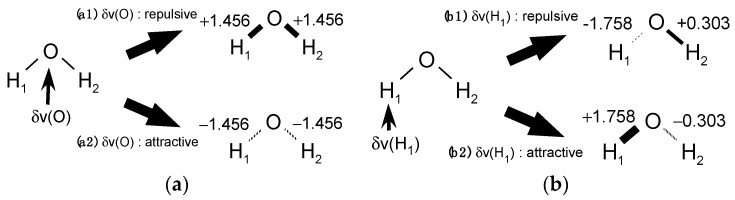
Two-fold meanings of the LRF-BO values, for which the perturbations are applied to (**a**) the oxygen atom; and (**b**) the left hydrogen atom.

Then, for the case of Figure 1a, the LRF-BO value, $\delta B^{\mathrm{OH}}/\delta v(O)=1.456$, indicates not only that the repulsive potential to the oxygen site strengthens O-H bonds of water, but also that the attractive potential weakens the bond, of which situations are illustrated in Figure 2a. Similarly, the meaning of $\delta B/\delta {v(H}_{1})$ values shown in Figure 1b is two-fold, as shown in Figure 2b. 

We summarized the relation among the sign of the LRF-BO values, the sign of the perturbation, and the change of the bond-order in Table 1. For a specific LRF-BO value, there are two cases concerning the sign of the virtual perturbation, which leads to two situations for the molecular system. Thus, we have to choose the sign of the virtual perturbation that is appropriate for the situation we consider. For example, it can be deduced from Figure 2(a2),(b1) that an attractive δv(O) and an repulsive $\delta {v(H}_{1})$ induce the dissociation of the O-H_1_ bond of the water molecule, which corresponds to a decrease of the p*K*_a_ value of water due to the ligation of H_2_O to a metal ion, which is a well-known fact in inorganic chemistry [26],

(15) $$H_{2}O\;+\;M^{2+}\to {\mathrm{M-OH}}^{+}+H^{+}.$$

For the reaction in water solution, this chemical reaction formula is rewritten in a more realistic form,

(16) $$H_{2}O\;+\;M^{2+}+H_{2}O\;\to {\mathrm{M-OH}}^{+}+H_{3}^{+}O.$$

For instance, when a water molecule ligates to $M^{2+}={\mathrm{Zn}}^{2+}$, the p*K*_a_ value decreases from 14.0 to 10.0 [26]. As an interpretation of the LRF-BO results shown in Figure 2(a2),(b1), the attractive δv(O) plays the role of the Zn^2+^ ion and the repulsive $\delta {v(H}_{1})$ the role of the oxygen site of the additional H_2_O at the left side of Equation (16) to receive the proton and to form the hydronium ion. What we would like to emphasize here is that the signs of the virtual perturbation should be appropriately determined when we interpret calculated LRF-BO values in the context of chemical reactions. 

In the remaining part, the virtual perturbation will be assumed to be repulsive for electrons unless stated otherwise.

### 3.2. Various Types of Chemical Bonds 

We first calculated the LRF-BO values of H_2_ and H-X (X = F, Cl, Br, I) to check dependency of LRF-BO values on polarity of covalent bonds. For H_2_, the calculated LRF-BO value becomes zero. This fact is straightforwardly derived from Equation (1): the leading term of the linear response function of density matrix is given by,

(17) $$\begin{array}{cc} \frac{\delta \rho (r_{1},r_{2})}{\delta v(r)} & ≅\sum_{\sigma }^{\alpha ,\beta }\frac{\psi_{LUMO}^{\sigma }(r)\psi_{HOMO}^{\sigma }(r)}{\varepsilon_{HOMO}^{\sigma }-\varepsilon_{LUMO}^{\sigma }}(\psi_{LUMO}^{\sigma }(r_{1})\psi_{HOMO}^{\sigma }(r_{2})+\psi_{HOMO}^{\sigma }(r_{1})\psi_{LUMO}^{\sigma }(r_{2}))\\ & \propto \sum_{\sigma }^{\alpha ,\beta }\frac{\psi_{LUMO}^{\sigma }(r)\psi_{HOMO}^{\sigma }(r)}{\varepsilon_{HOMO}^{\sigma }-\varepsilon_{LUMO}^{\sigma }}(\chi_{1}(r_{1})\chi_{1}(r_{2})-\chi_{2}(r_{1})\chi_{2}(r_{2})) \end{array}.$$

**Figure 3:**
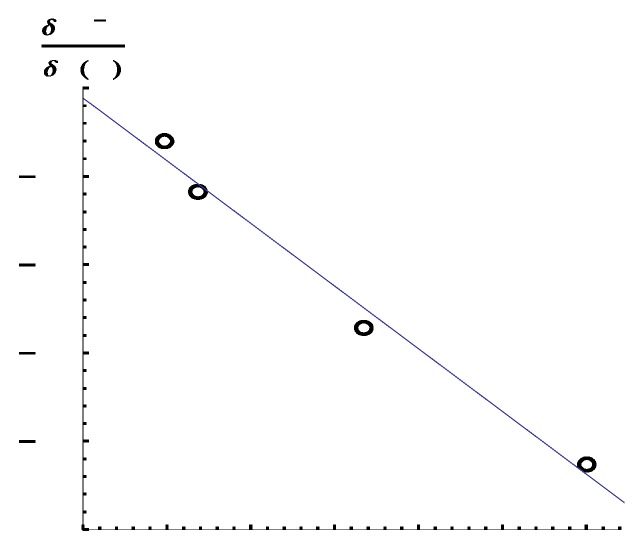
The relationship between the Mulliken charges on the hydrogen atom and $\delta B^{\mathrm{HX}}/\delta v(H)$ values for H-X (X = F, Cl, Br, I).

Here, $\chi_{1}$ and $\chi_{2}$ are atomic orbitals of hydrogens of H_2_. As can be seen from Equation (17), the cross terms, $\chi_{1}(r_{1})\chi_{2}(r_{2})$ and $\chi_{2}(r_{1})\chi_{1}(r_{2})$, disappear, which makes $\delta P_{\mu \nu }/\delta v\left(r\right)\left(\mu \in 1,\nu \in 2\right)$, and so $\delta B^{\mathrm{IJ}}/\delta v(r)$, zero. In contrast, the LRF-BO values of the halogen halides are non-zero: when the case that the perturbation is applied to the hydrogen atom the values, $\delta B^{\mathrm{HX}}/\delta v(H)$, of H-X (X= F, Cl, Br, I) are −2.132, −1.358, −0.58724, and −0.300, respectively. In Figure 3, we show the relation between the calculated Mulliken charges on the hydrogen atom and the $\delta B^{\mathrm{HX}}/\delta v(H)$ values. From this figure, we can see that a linear relationship holds. Note that this relation holds only for simple two-center two-electrons polar covalent systems (for details of the analysis of the results, see Appendix A). We would like to point out that the negative $\delta B^{\mathrm{HX}}/\delta v(H)$ values indicate that repulsive potentials to electrons on hydrogen atoms induced the dissociation of the bonds, being consistent with a reasonable picture of chemical reactions. 

**Figure 4:**
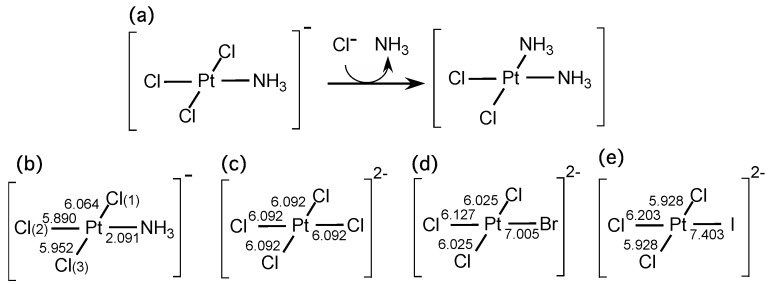
(**a**) The substitution reaction step to yield cisplatin. Calculated LRF-BO values for (**b**) [Pt(NH_3_)Cl_3_]^−^; (**c**) [PtCl_4_]^2−^; (**d**) [PtBrCl_3_]^2−^; and (**e**) [PtBrCl_3_]^2−^.

Next, we investigated coordination bonding systems. It is known that a ligand that lies trans to the leaving group increases the rate of the substitution if the ligand is a strong donor or a strong π acceptor. This is called the trans-effect, which is described in most standard text books of inorganic chemistry [27,28]. A typical example is the reaction step to yield cisplatin, which is illustrated in Figure 4a. In this case, it is known that the substitution reaction occurs next to the ammonium ligand (NH_3_), not trans to NH_3_, because the trans effect of Cl^−^ is larger than that of NH_3_ [27]. In order to check whether the LRF-BO values could be an indicator to the trans-effect, we calculated the LRF-BO values, ${\left\{\delta B^{\mathrm{PtX}}/\delta v(X)\right\}}_{{X=\mathrm{Cl}(1),\mathrm{Cl}(2),\mathrm{Cl}(3),\mathrm{NH}}_{3}}$ for [Pt(NH_3_)Cl_3_]^−^, the geometry of which is optimized with the B3LYP method. We employed the LANL2TZ(f) basis set for Pt atom, and 6-311G** basis sets for other atoms. As shown in Figure 4b, the obtained $\delta B^{\mathrm{PtX}}/\delta v(X)$ values are 6.064, 5.890, 5.952, and 2.091, for X = Cl(1), Cl(2), Cl(3), and NH_3_, respectively. On the basis of these results, the substitution reaction is predicted to occur at the Cl(1) site, not the Cl(2) site, being consistent with the substitution reaction to yield cisplatin. We have to comment that that non-negligible difference between $\delta B^{\mathrm{PtCl}(1)}/\delta v(\mathrm{Cl}(1))$ and $\delta B^{\mathrm{PtCl}(3)}/\delta v(\mathrm{Cl}(3))$ is due to the position of protons of NH_3_: one of the protons lies within the planar consisting of Pt, N, and three Cl atoms, nearby Cl(1), enhancing $\delta B^{\mathrm{PtCl}(1)}/\delta v(\mathrm{Cl}(1))$. We further examined the LRF-BO values of [PtXCl_3_]^2−^ for, X = Cl, Br, I, the results of which are presented in Figure 4c–e. The point is that the calculated LRF-BO values are in order, $\delta B^{\mathrm{PtCl}}/\delta v\left(\mathrm{Cl}\right)<\delta B^{\mathrm{PtBr}}/\delta v\left(\mathrm{Br}\right)<\delta B^{\mathrm{PtI}}/\delta v\left(I\right)$, being consistent with the trans-effect series of halogen ions, ${\mathrm{Cl}}^{-}<{\mathrm{Br}}^{-}<I^{-}$ [28]. In addition, it is noteworthy that all the values $\delta B^{\mathrm{PtX}}/\delta v(X)$ are positive, implying that an electrophilic attack induces the elimination of the leaving ions or the leaving group (see Table 1): in short, the LRF-BO values provide a reasonable qualitative picture of the chemical reactions as well.

### 3.3. Inductive and Resonance Effects of Organic Molecules

**Figure 5:**
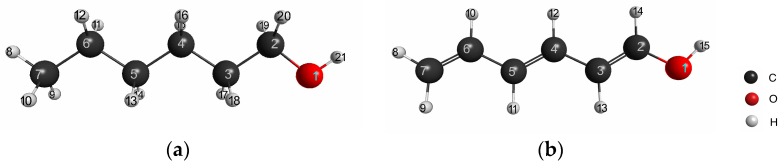
Geometries of (**a**) hexan-1-ol and (**b**) hexa-1,3,5-trien-1-ol molecules.

Next, we calculated the LRF-BOs of the saturated and unsaturated (conjugated) molecules, which has been one of the standard systems for testing of the applicability of the linear response function of density (LRF-D) to inductive and resonance effects of organic molecules [9,14]. For this purpose, we picked out hexan-1-ol and hexa-1,3,5-trien-1-ol, the molecular structures of which are illustrated in Figure 5a,b. Before testing the LRF-BO values, we computed atom-condensed LRF-D values, which are defined by,

(18) $$\frac{\delta \rho (K)}{\delta v(L)}≅\int^{L}dr\int^{K}dr^{\prime }\frac{\delta \rho (r^{\prime })}{\delta v(r)}.$$

Here K-th and L-th atom’s center integrals are similar to that defined in Equation (13). The atom numbering is given in Figure 5a,b. In this case, the virtual perturbation is placed on the oxygen of the hydroxyl group, which is denoted as δv(1).

**Figure 6:**
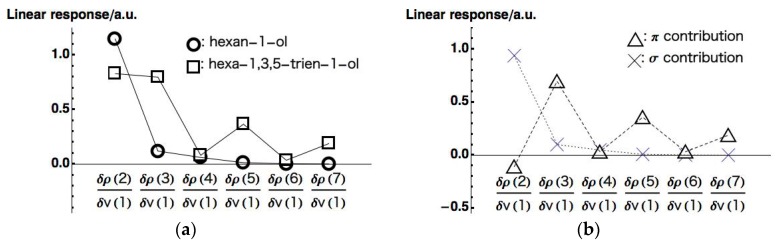
(**a**) Linear response function of density (LRF-Ds) of hexan-1-ol and hexa-1,3,5-trien-1-ol molecules; (**b**) σ and π contributions of LRF-D of the hexa-1,3,5-trien-1-ol molecules.

The responses on all carbons (δρ(2)~δρ(7)) for hexan-1-ol and hexa-1,3,5-trien-1-ol are plotted in Figure 6a (to be complete, we present all the LRF-D values in Tables S1–S3 in supplementary materials for hexan-1-ol, hexa-1,3,5-trien-1-ol, and π contributions of hexa-1,3,5-trien-1-ol, respectively). We can see from this figure that the response of hexan-1-ol decreases monotonically and rapidly from the nearest site to the farthest site, being consistent with the picture of the inductive effect of the saturated system. This contrasts with the response on the conjugated chain of hexa-1,3,5-trien-1-ol, which also decays from δρ(2) to δρ(7) but with the oscillating behavior. To analyze the behavior of the LRF-D values further, we divided the LRF-D values into σ and π contributions according to the method Fias et al. used [13]. The results are shown in Figure 6b. This figure shows that the σ contribution of the LRF-D of hexa-1,3,5-trien-1-ol is similar to that of hexan-1-ol. In particular, we found that the plus value of δρ(2)/δv(1) of hexa-1,3,5-trien-1-ol is a result of the inductive effect mainly from the σ contribution. On the other hand, being maximum at ${\left\{\delta \rho (n)/\delta v(1)\right\}}_{n=3,5,7}$ of the π contribution obviously corresponds to the resonance picture of the π conjugated network (see Figure 6a below), implying that LRF-D becomes an indicator of density fluctuations that are results from inductive and resonance effects of organic molecules. These results are similar to those of reference [14], indicating that our numerical treatment is valid for our purposes.

**Figure 7:**
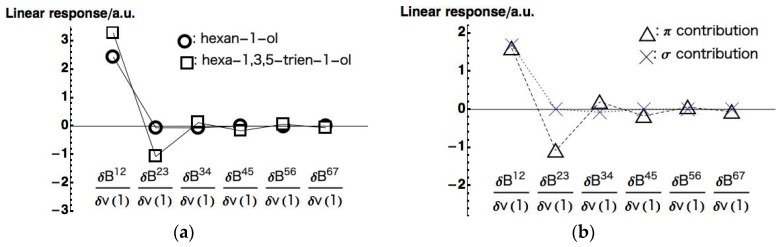
(**a**) LRF-BOs of hexan-1-ol and hexa-1,3,5-trien-1-ol molecules; (**b**) σ and π contributions of LRF-BO of the hexa-1,3,5-trien-1-ol molecules.

We then evaluated the LRF-BO values of all chemical bonds on the main chain for the perturbation, δv(1). Figure 7a shows the LRF-BO values for the chemical bonds of the main chains of the hexan-1-ol and hexa-1,3,5-trien-1-ol (all calculated LRF-BO values are presented in Tables S4–S6 in supplementary materials for hexan-1-ol, hexa-1,3,5-trien-1-ol, and π contributions of hexa-1,3,5-trien-1-ol, respectively). It is found from Figure 7a that the fluctuation of bond-orders of the hexan-1-ol molecule is nearly localized in the bond between O(1) and C(2). In contrast, the profile of the LRF-BO values of hexa-1,3,5-trien-1-ol, in which points are indicated as squares, exhibits a oscillating behavior. As in the case of LRF-D values, we divide the LRF-BO values of the hexa-1,3,5-trien-1-ol into σ and π contributions, which are shown in Figure 7b. We can see from this figure that the σ contribution indicated by the X points shows the behavior similar to that of the hexan-1-ol molecule shown in Figure 7a, while the π contribution indicated by the triangular points is obviously a main cause of the oscillating behavior of the total LRF-BO values. 

**Figure 8:**
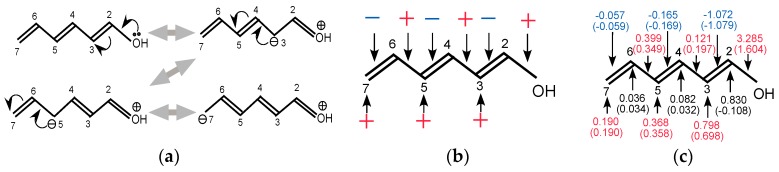
(**a**) Resonance structure of hexa-1,3,5-trien-1-ol molecule; (**b**) Expected fluctuations of charges and bond-orders; (**c**) LRF-D and LRF-BO values for which the perturbation is applied to the oxygen atom.

It should be noted that the resonance effect of the conjugate system shown in Figure 8a is described not only with the density fluctuations on the atomic sites but also with the fluctuations of chemical bonds on the main chain. From Figure 8a, the averages of density fluctuations and the bond fluctuations are expected to be those shown in Figure 8b. Here + indicates the sites where the density or the bond-order increases and—the sites where the density or the bond-order decreases. We summarized the calculated LRF-D and LRF-BO values, together with those of the π contributions in parentheses, in Figure 8c. The values in parentheses in Figure 8c are obviously consistent with the resonance picture illustrated in Figure 8b and the inductive effect on the charge fluctuation mainly on C(2), implying that the LRF-BO and the LRF-D complement each other, enabling us to estimate inductive and resonance effects quantitatively.

### 3.4. Acid Dissociation Reaction of Substituted Benzoic Acids

**Figure 9:**
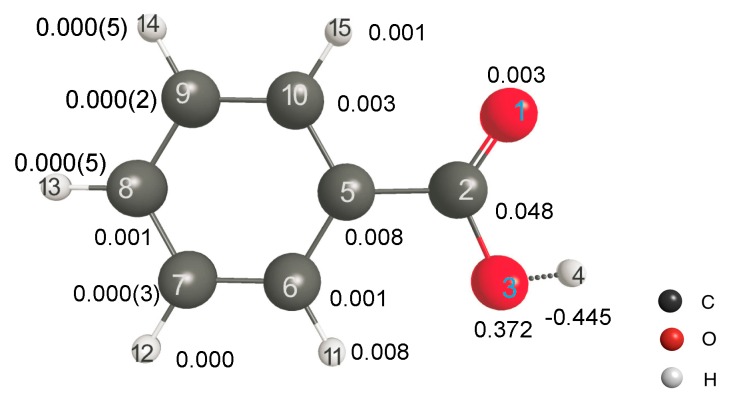
Calculated LRF-BO values for the acid dissociation mode, ${\left\{\delta B^{\text{O-H}}/\delta v(L)\right\}}_{L}^{\mathrm{All}\;\mathrm{atoms}}$, of the benzoic acid molecule.

Finally, we would like to explore the applicability of LRF-BO to the acid dissociation reaction of benzoic acids. We shall focus on the response of the bond-order between the oxygen and the hydrogen atoms of the carboxylate for the virtual perturbation that is applied to each atom in the benzoic acid molecule. In Figure 9, we listed the calculated results of the LRF-BO values, ${\left\{\delta B^{\text{O-H}}/\delta v(L)\right\}}_{L}^{\mathrm{All}\;\mathrm{atoms}}$. The response values are remarkably large for the case that the perturbation is placed on or nearby the carboxylate, in particular on O(14) and H(15) atoms of which the target O-H bond is composed, while the values become nearly zero for the perturbation that is applied to the other (phenyl) part. Not surprisingly, the absolute value of LRF-BO, $\left|\delta B^{\text{O-H}}/\delta v(L)\right|$ is maximum for the case, L = H(4), implying that the bond order, $B^{\text{O-H}}$, is most sensitive for the perturbation to the proton to be eliminated.

The substitution effect of benzoic acids is one of the most well-investigated topics among chemical reactions of organic molecules [29,30,31]. Now we will consider dissociation reactions of non-substituted and substituted benzoic acids,

(19) $$C_{6}H_{5}\mathrm{COOH}\;\overset{K_{0}}{↔}C_{6}H_{5}{\mathrm{COO}}^{-}+H^{+},$$

(20) $${\text{m-XC}}_{6}H_{4}\mathrm{COOH}\;\overset{K_{X}^{m}}{↔}{\text{m-XC}}_{6}H_{5}{\mathrm{COO}}^{-}+H^{+},$$

(21) $${\text{p-XC}}_{6}H_{4}\mathrm{COOH}\;\overset{K_{X}^{p}}{↔}{\text{p-XC}}_{6}H_{5}{\mathrm{COO}}^{-}+H^{+}.$$

Here K_0_, $K_{X}^{m}$ and $K_{X}^{p}$ are equilibrium constants for non-substituted, meta-substituted, and para-substituted benzoic acids, respectively. The logarithm of the ratio of the equilibrium constants is called the Hammett constant, which are defined by,

(22) $$\sigma_{X}^{n}\equiv \log\frac{K_{X}^{n}}{K_{0}}\;(n=m\;or\;p).$$

Hammett pointed out that the Hammett constant is proportional to the logarithm of the ratio of the rate constants for substituted benzoic acids [29]. In other words, the Hammett constant determines substitution effects on both kinetics and thermodynamics for this class of systems. Note that we use two simple sets of reaction-independent substituent constants, $\sigma_{X}^{n}(n=m\;or\;p)$, rather than more complicated constants (there are more than 20 sets! see ref. [31]), by which the researchers divided the effects and classified the types of reactions to describe the reactions in more details. This is because our intention is to lump many effects together to determine equilibrium constants of acid dissociation reactions as far as possible and to test the applicability of the LRF-BO as a descriptor for them. 

**Figure 10:**
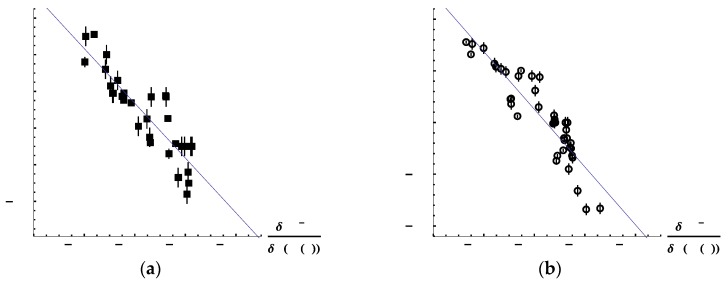
Correlation between Hammett constants and $\delta B^{\text{O-H}}/\delta v(H(4))$ values at the B3LYP/6-311G** level. (**a**) Meta-substituted benzoic acids; (**b**) Para-substituted benzoic acids.

Thus, we inspected the correlation between the Hammett constants and the calculated LRF-BO values of substituted benzoic acids in order to examine whether the LRF-BO could be a descriptor to cover the substitution effects. For this purpose, we picked out meta- and para-substituted benzoic acids, of which the errors of the experimentally determined Hammett constants are estimated to be within 0.1, from ref. [30]. We exclude all the cases that a substituent group has a positive or negative charge, because, for such cases, we have to estimate LRF-BO values of hydrated clusters, of which the structures must highly fluctuate and so which are beyond the scope of our approach. First, we examined the case in which the absolute value of the LRF-BO values takes maximum, i.e., the case, L = H(4). Figure 10a,b plot the correlations between Hammett constants and LRF-BO values for meta- and para-substituted benzoic acids, respectively. In these figures, error bars are also presented to indicate the estimated errors of the experimental data [30]. We can see from these figures that there are linear relationships for both cases. The coefficients of determination (R^2^) are calculated to be 0.772 and 0.828 for meta- and para-substituted benzoicacids, respectively. These results imply that the LRF-BO values could be useful to predict the change of the Hammett constant, i.e., p*K*_a_ as well, for a new substituent. 

When considering the reason why the linear relationships hold, it is noteworthy that $\log K_{X}^{n}/K_{0}\;(n=m\;or\;p)$ is proportional to the difference between the changes of the Gibb’s energies for the acid dissociation reactions of the non-substituted and the substituted benzoic acids, i.e.,

(23) $$\sigma_{X}^{n}=\log\frac{K_{X}^{n}}{K_{0}}\propto \Delta G_{0}-\Delta G_{X}^{n}\;(n=m\;or\;p),$$

where $\Delta G_{X}^{n}$ is the change of Gibb’s energies for a substituted (X-) benzoic acid and $\Delta G_{0}$ is that for the non-substituted benzoic acid. Thus, the linear relationship between the Hammett constants and the LRF-BO values indicates that the LRF-BO is closely related to the difference of the Gibb’s free energies between the reactant state and the product state. 

For completeness, we also examined the correlation between the Hammett constants and the LRF-BO values for the perturbation on each atom in benzoic acids and presented the resulting coefficients of determination in Figure S2a,b for meta- and para-substituted benzoic acids respectively, in the supplementary materials. Also, we listed all LRF-BO values of meta and para substituted benzoic acids in Tables S7 and S8 respectively. Surprisingly, in some of the cases that the virtual perturbation is applied to atoms in the phenyl part, we obtained large coefficients of determination values. Nevertheless, from Tables S7 and S8, the magnitudes of the LRF-BO values are found to be considerably small for such cases. This implies that although the bond-order between O and H in the carboxylate is remarkably sensitive to the perturbation at O(14) and H(15) in the carboxylate part, the description of substitution effects are affected by the perturbation not only of the O(14) and H(15) part, but also of the phenyl part because the induced and resonance effects work through the phenyl part. We also checked the basis set dependence of the results for 6-31G, 6-31G**, 6-31++G**, 6-311G, and 6-311++G**. See Tables S9–S18, and Figures S3–S6. A noteworthy point is that the use of diffuse functions (Figrues S5 and S7) deteriorates the correlation between Hammett constants and LRF-BO values. This is due to a well-known fact that the Mulliken type of population analyses often fails when the diffuse function is used [32,33].

As shown above, we actually found that the LRF-BO values could be useful in predicting the Hammett constants for new substitutions on the basis of the linear relationship if we use an appropriate basis set. However, we have to note that the LRF-BO values provide only rough estimations of the Hammett constants, i.e., rough estimations of the p*K*_a_ values. In the field of computational chemistry, the estimation of p*K*_a_ values of molecular species in various situations such as in solutions and in reaction centers of enzymes has been a hot topic. Many researchers have developed convenient and accurate methods to estimate p*K*_a_ values [34,35,36,37,38], and by using these methods, nowadays we would be able to estimate p*K*_a_ values much more accurately than R^2^ ~0.8 as we presented by using LRF-BO values. Our results are meaningful in another aspect, however, because the results shown in Figure 9 and Tables S7 and S8 are directly related to the mechanism of acid dissociation reactions: the large negative values of $\delta B^{\text{O-H}}/\delta v(H(4))$ indicate that a nucleophilic attack primarily induces the dissociation of the O-H bond, i.e., the acid dissociation, and the negative values of $\delta B^{\text{O-H}}/\delta v(O(3))$ indicate that an electrophilic effect on O(3) secondarily supports it. Although, to our knowledge, the detailed molecular mechanism of the acid dissociation of benzoic acids has not been investigated experimentally, our results are obviously reasonable from the viewpoint of chemical intuition. In addition, we would like to point out that a recent metadynamics calculation based on ab initio Car-Parinello molecular dynamics showed that the attack of water to the proton leads to form a non-dissociating meta-stable state along the reaction path (See Figure 1a, Figure S1, and the movie S1 [38]), which is consistent with our results. This implies that the LRF-BO values of molecules encode essential information about the reaction mechanism of the target molecules, which is one of the most important conditions for being a good descriptor (See Introduction [39]).

## 4. Conclusions and Future Directions

We presented the linear response function of bond-order (LRF-BO) based on a real space integration scheme. In order to exemplify the way to interpret the LRF-BO values of molecules, we first applied it to a water molecule. Next, we examined the LRF-BO values of various types of chemical bonding systems. We presented the fundamental features of the LRF-BO values for non-polar and polar covalent bonding systems. In addition, we see that LRF-BO could be an indicator to trans-effects of coordination bond systems. Then, we computed conjugated and non-conjugated organic systems, and substituted benzoic acids and showed that the LRF-BO value is a complementary property to the linear response function of density (LRF-D), which has often been used to describe inductive and resonance effects of organic molecules. We also investigated the LRF-BO values for the acid dissociation mode of benzoic acids. Examining perturbations to all atoms of the basic structure of benzoic acids, we found that the virtual perturbation to the proton to be eliminated leads to the maximum response on the O-H bond. In addition, we presented that there are significant correlations between the Hammett constants and some of LRF-BO values, $\delta B^{\text{O-H}}/\delta v(L)$ for substituted benzoic acids. An important point is that the magnitudes and signs of the LRF-BO values, as well as the coefficients of determination for the correlations, strongly depend on the site, L, to which the virtual perturbation is applied. From our computations, it is supposed that the leading step of the acid dissociations is to a nucleophilic attack to the proton to be eliminated, which is consistent with the chemical intuition and a recent study of the reaction mechanism based on ab initio Car-Parinello MD calculations. We would like to emphasize that the LRF-BO is able to include information combining the fluctuations of specific chemical bonds in molecules and the specific virtual perturbations to induce the fluctuations. We can expect that the LRF-BO will become a new standard molecular descriptor [39], which is useful for molecular design in various fields.

## Figures and Tables

**Table 1:** Relation among the sign of LRF-BO, the sign of the perturbation, and the change of the bond-order.

Sign of A LRF-BO Value	Plus ($\delta B^{\mathrm{IJ}}/\delta v(X)\;>\;0$)		Minus ($\delta B^{\mathrm{IJ}}/\delta v(X)\;<\;0$)	
A virtual perturbation (δv(X))	Repulsive (+)	Attractive (−)	Repulsive (+)	Attractive (−)
Change of the Bond-order ($\delta B^{\mathrm{IJ}}$)	Increase (+)	Decrease (−)	Decrease (−)	Increase (+)

## Supplementary Materials

Supplementary materials can be found at www.mdpi.com/1422-0067/17/10/1779/s1.

## Appendix A

In this appendix, we show the reason why the linear relationship between charges and $\delta B^{\mathrm{HX}}/\delta v(H)$ holds for H-X (X = F, Cl, Br, I). First, we assume that the target system is a two-center two electrons polar-covalent bonding system. For such systems, the HOMO and LUMO can be written by,

(A1) $$\psi_{\mathrm{HOMO}}\left(r\right)=\cos\theta \phi_{b}\left(r\right)+\sin\theta \phi_{a}\left(r\right),\;\psi_{\mathrm{LUMO}}\left(r\right)=-\sin\theta \phi_{b}\left(r\right)+\cos\theta \phi_{a}\left(r\right).$$

Here $\phi_{b}$ and $\phi_{a}$ are bonding and antibonding orbitals consisting of two atomic orbitals, $\chi_{1}$ and $\chi_{2}$ as,

(A2) $$\phi_{b}\left(r\right)=\frac{1}{\sqrt{2}}\left(\chi_{1}\left(r\right)+\chi_{2}\left(r\right)\right),\phi_{a}\left(r\right)=\frac{1}{\sqrt{2}}=\left(\chi_{1}\left(r\right)-\chi_{2}\left(r\right)\right)$$

We note that the spin indices and the overlap integral between atomic orbitals are omitted for simplicity of formulation. In this case, we can straightforwardly obtained

(A3) $$\begin{array}{l} \psi_{LUMO}(r_{1})\psi_{HOMO}(r_{2})+\psi_{HOMO}(r_{1})\psi_{LUMO}(r_{2})\\ =\frac{1}{2}\left[\cos2\theta \left(\chi_{1}(r_{1})\chi_{1}(r_{2})-\chi_{2}(r_{1})\chi_{2}(r_{2})\right)-\sin2\theta \left(\chi_{1}(r_{1})\chi_{2}(r_{2})+\chi_{2}(r_{1})\chi_{1}(r_{2})\right)\right] \end{array}$$

and

(A4) $$\int^{1\mathrm{st atom}}dr\psi_{LUMO}\left(r\right)\psi_{HOMO}\left(r\right)≅\frac{1}{2}\cos2\theta .$$

Thus, from Equations (1) to (3), the following relation holds for the linear response function of the density matrix for the perturbation applied to the first atom (δv(1)):

(A5) $$\frac{\delta P_{12}}{\delta v(1)}\propto \frac{\sin2\theta }{\cos2\theta }.$$

Here $P_{12}$ denotes the element for the atomic orbital pair, $\chi_{1}$ and $\chi_{2}$. We can also have

(A6) $$P_{12}\propto \cos2\theta$$

so that the linear response function of bond-order between the first and second atom can be approximated as,

(A7) $$\begin{array}{c} \delta B^{12}/\delta v(1)≅\frac{\delta P_{12}}{\delta v(1)}P_{12}+P_{12}\frac{\delta P_{12}}{\delta v(1)}\\ \propto \sin2\theta \end{array}.$$

Then, the atomic (polarization) charge at the 1 st atom is also given by, sin2θ, which can be derived from the form of HOMO given by (A-1). Therefore, the LRF-BO value, $\delta B^{12}/\delta v(1)$, is proportional to the atomic charge. In addition, the non-polar covalent bonding system (for instance, H_2_) satisfies this relation because both $\delta B^{12}/\delta v(1)$ and atomic polarization charge become zero. However, we should note that this relation holds only for simple two-center two-electrons covalent bond: for general cases that the HOMO and LUMO and other active orbitals are delocalized over many sites, the above equations are not satisfied.
