# Supplementary material for: Linear Response Function of Bond-Order
Source: Int J Mol Sci. 2016 Oct 25;17(11):1779. doi: 10.3390/ijms17111779 (PMC5133780; doi:10.3390/ijms17111779)
Supplement: Supplementary file 1 [file ijms-17-01779-s001.pdf]

# Supplementary Materials: Linear Response Function of Bond-Order

**Nayuta Suzuki, Yuki Mitsuta, Mitsutaka Okumura and Shusuke Yamanaka**

## 1. Linear Response Function of Bond-Order

Now we move on to the definition of linear response function of bond-order. As for the bond-order, we employ the Mayer bond order [1], the most standard definition of the bond-order in the field of ab initio quantum chemistry, which is defined as,

$$\mathbf{B}^{\mathrm{IJ}} \equiv \sum_{\mu}^{\mathrm{I}} \sum_{\nu}^{\mathrm{J}} \mathbf{Q}_{\mu\nu} \mathbf{Q}_{\nu\mu}. \tag{1}$$

The two summations of basis sets' indices, $\mu$ and $\nu$, run over the I-th atom and the J-th atom, respectively and the matrix, **Q,** is the product of the density matrix (**P**) and the overlap matrix (**S**),

$$\mathbf{Q}_{\mu\nu} \equiv \sum_{\eta} \mathbf{P}_{\mu\eta} \mathbf{S}_{\eta\nu}. \tag{2}$$

From Equation (3), we have an expression for the linear response function of the **Q** matrix as

$$\frac{\delta \mathbf{Q}_{\mu\nu}}{\delta \mathrm{v}(\mathbf{r})} = \sum_{\sigma}^{\alpha,\beta} \sum_{ij} \frac{\psi_j^{\sigma}(\mathbf{r}) \psi_i^{\sigma}(\mathbf{r})}{\varepsilon_i^{\sigma} - \varepsilon_j^{\sigma}} \sum_{\eta} (C_{j\mu}^{\sigma} C_{i\eta}^{\sigma} + C_{i\mu}^{\sigma} C_{j\eta}^{\sigma}) \mathbf{S}_{\eta\nu}. \tag{3}$$

Finally, the linear response function of the bond-order can then be defined as

$$\delta \mathbf{B}^{\mathrm{IJ}} / \delta \mathrm{v}(\mathbf{r}) \equiv \sum_{\mu}^{\mathrm{I}} \sum_{\nu}^{\mathrm{J}} \left( \frac{\delta \mathbf{Q}_{\mu\nu}}{\delta \mathrm{v}(\mathbf{r})} \mathbf{Q}_{\nu\mu} + \mathbf{Q}_{\mu\nu} \frac{\delta \mathbf{Q}_{\nu\mu}}{\delta \mathrm{v}(\mathbf{r})} \right). \tag{4}$$

It is convenient to consider perturbations that are applied to atomic sites. For this purpose, we define the LRF-BO for the local perturbation, $\delta \mathrm{v}(\xi)$, that is applied to a specific atomic orbital $\phi_{\xi}$ via the following relation,

$$\sum_{\xi} \frac{\delta \mathbf{B}^{\mathrm{IJ}}}{\delta \mathrm{v}(\xi)} \equiv \int d\mathbf{r} \frac{\delta \mathbf{B}^{\mathrm{IJ}}}{\delta \mathrm{v}(\mathbf{r})} \tag{5}$$

and we then have an expression of LRF-BO for the perturbation to an L-th atom,

$$\frac{\delta \mathbf{B}^{\mathrm{IJ}}}{\delta v(\mathrm{L})} \equiv \sum_{\xi}^{\mathrm{L}} \frac{\delta \mathbf{B}^{\mathrm{IJ}}}{\delta v(\xi)}. \tag{6}$$

Here, the summation, $\sum_{\xi}$, at the left side of Equation (8) runs over all AOs, and that at the right side of Equation (9), $\sum_{\xi}^{\mathrm{L}}$ is limited to the AOs that belong to the L-th atom. This is the scheme we employed in reference [2], which is the first implementation of the LRF-BO. The LRF-BO based on AO perturbations (Equation (8)) suits the linear combination of AOs (LCAO) formalism: for instance, we easily see the LRF-BO for the case that the perturbation is applied to a $\pi$ orbital at a specific carbon atom in a $\pi$-conjugated system. However, when we would like to see the effects due to a nucleophilic or electrophilic attack to a specific atom, it is unclear whether the perturbation, $\delta \mathrm{v}(\xi)$, is attractive or repulsive since atomic orbitals except 1s orbitals have different (positive and negative) phases' parts in their distributions. To avoid such ambiguity, we here define $\delta \mathbf{B}^{\mathrm{IJ}} / \delta \mathrm{v}(\mathrm{L})$ using a numerical integration

$$\frac{\delta \mathbf{B}^{\mathrm{IJ}}}{\delta \mathrm{v}(\mathrm{L})} \equiv \int^{\mathrm{L}} d\mathbf{r} \frac{\delta \mathbf{B}^{\mathrm{IJ}}}{\delta \mathrm{v}(\mathbf{r})}, \tag{7}$$

where the domain of integration for the L-th atom at the left side is defined as the Wigner-Seitz cell. The Wigner-Seitz cell for molecular systems is constructed in a similar manner to that of solid

systems: the region for a specific atom in the molecule can be defined as a region encircled by all perpendicular bisectors with neighboring atoms (See Figure S1).

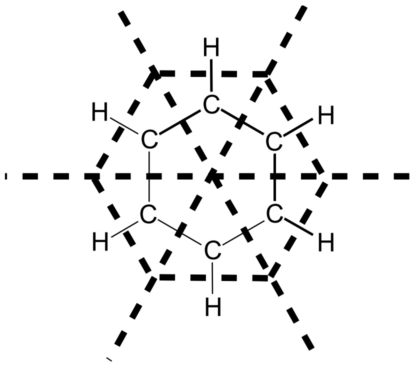


**Figure S1.** An example of Wigner-Seitz cells for molecular systems ($C_6H_6$). The region for each atom is defined as the region encircled by dashed spaced lines. For the terminal atoms (in this case, 6 hydrogens), the regions spread to infinity.

## 2. Computational Results

### *2.1. Inductive and Resonance Effects of Organic Molecules*

The responses on all carbons ($\delta\rho(2)$~$\delta\rho(7)$) for hexan-1-ol and hexa-1,3,5-trien-1-ol are plotted in Figure 6a (to be complete, we present all the LRF-D values in Tables S1–S3 in supplementary materials for hexan-1-ol, hexa-1,3,5-trien-1-ol, and $\pi$ contributions of hexa-1,3,5- trien-1-ol, respectively). We can see from this figure that the response of hexan-1-ol decreases monotonically and rapidly from the nearest site to the farthest site, being consistent with the picture of the inductive effect of the saturated system. This contrasts with the response on the conjugated chain of hexa-1,3,5-trien-1-ol, which also decays from $\delta\rho(2)$ to $\delta\rho(7)$ but with the oscillating behavior. To analyze the behavior of the LRF-D values further, we divided the LRF-D values into $\sigma$ and $\pi$ contributions according to the method Fias et al. used [3]. The results are shown in Figure 6b. This figure shows that the $\sigma$ contribution of the LRF-D of hexa-1,3,5-trien-1-ol is similar to that of hexan-1-ol. In particular, we found that the plus value of $\delta\rho(2)/\delta\mathrm{v}(1)$ of hexa-1,3,5-trien-1-ol is a result of the inductive effect mainly from the $\sigma$ contribution. On the other hand, being maximum at $\{\delta\rho(\mathrm{n})/\delta\mathrm{v}(1)\}_{n=3,5,7}$ of the $\pi$ contribution obviously corresponds to the resonance picture of the $\pi$ conjugated network (see Figure 6a below), implying that LRF-D becomes an indicator of density fluctuations that are results from inductive and resonance effects of organic molecules. These results are similar to those of reference [3], indicating that our numerical treatment is valid for our purposes.

We then evaluated the LRF-BO values of all chemical bonds on the main chain for the perturbation, $\delta\mathrm{v}(1)$. Figure 7a shows the LRF-BO values for the chemical bonds of the main chains of the hexan-1-ol and hexa-1,3,5-trien-1-ol (all calculated LRF-BO values are presented in Tables S4–S6 in supplementary materials for hexan-1-ol, hexa-1,3,5-trien-1-ol, and $\pi$ contributions of hexa-1,3,5- trien-1-ol, respectively). It is found from Figure 7a that the fluctuation of bond-orders of the hexan-1-ol molecule is nearly localized in the bond between O(1) and C(2). In contrast, the profile of the LRF-BO values of hexa-1,3,5-trien-1-ol, in which points are indicated as squares, exhibits a oscillating behavior. As in the case of LRF-D values, we divide the LRF-BO values of the hexa-1,3,5-trien-1-ol into $\sigma$ and $\pi$ contributions, which are shown in Figure 7b. We can see from this figure that the $\sigma$ contribution indicated by the X points shows the behavior similar to that of the hexan-1-ol molecule shown in Figure 7a, while the $\pi$ contribution indicated by the triangular points is obviously a main cause of the oscillating behavior of the total LRF-BO values.

### *2.2. Acid Dissociation Reaction of Substituted Benzoic Acids*

For completeness, we also examined the correlation between the Hammett constants and the LRF-BO values for the perturbation on each atom in benzoic acids and presented the resulting coefficients of determination in Figure S2a,b for meta- and para- substituted benzoic acids respectively, in the supplementary materials. Also, we listed all LRF-BO values of meta and para substituted benzoic acids in Tables S7 and S8 respectively. Surprisingly, in some of the cases that the virtual perturbation is applied to atoms in the phenyl part, we obtained large coefficients of determination values. Nevertheless, from Tables S7 and S8, the magnitudes of the LRF-BO values are found to be considerably small for such cases. This implies that although the bond-order between O and H in the carboxylate is remarkably sensitive to the perturbation at O(14) and H(15) in the carboxylate part, the description of substitution effects are affected by the perturbation not only of the O(14) and H(15) part, but also of the phenyl part because the induced and resonance effects work through the phenyl part. We also checked the bais set dependence of the results for 6-31G, 6-31G**, 6-31++G**, 6-311G, and 6-311++G**. See Tables S9–S18, and Figures S3–S6. A noteworthy point is that the use of diffuse functions (Figures S5 and S7) deteriorates the correlation between Hammett constants and LRF-BO values. This is due to a well-known fact that the Mulliken type of population analyses often fails when the diffuse function is used [4,5].

**Table S1.** All linear response function of density (LRF-D) values of hexan-1-ol at the B3LYP/6-311G** level. The numbering of the atoms listed is the same as presented in Figure 5a in the text.

| | **1** | **2** | **3** | **4** | **5** | **6** | **7** | **8** | **9** | **10** | **11** | **12** | **13** | **14** | **15** | **16** | **17** | **18** | **19** | **20** | **21** |
|---|---|---|---|---|---|---|---|---|---|---|---|---|---|---|---|---|---|---|---|---|---|
| 1 | −3.67 | 1.152 | 0.12 | 0.061 | 0.013 | 0.003 | 0.001 | 0.001 | 0.000 | 0.000 | 0.001 | 0.001 | 0.002 | 0.002 | 0.02 | 0.02 | 0.027 | 0.027 | 0.341 | 0.341 | 1.537 |
| 2 | 1.152 | −4.865 | 0.879 | 0.101 | 0.05 | 0.015 | 0.003 | 0.002 | 0.000 | 0.000 | 0.001 | 0.001 | 0.011 | 0.011 | 0.033 | 0.033 | 0.173 | 0.173 | 0.991 | 0.991 | 0.242 |
| 3 | 0.12 | 0.879 | −4.998 | 0.896 | 0.095 | 0.05 | 0.013 | 0.006 | 0.002 | 0.002 | 0.011 | 0.011 | 0.036 | 0.036 | 0.179 | 0.179 | 1.023 | 1.023 | 0.179 | 0.179 | 0.079 |
| 4 | 0.061 | 0.101 | 0.896 | −5.062 | 0.896 | 0.095 | 0.048 | 0.025 | 0.011 | 0.011 | 0.037 | 0.037 | 0.176 | 0.176 | 1.023 | 1.023 | 0.176 | 0.176 | 0.035 | 0.035 | 0.027 |
| 5 | 0.013 | 0.05 | 0.095 | 0.896 | −5.069 | 0.898 | 0.085 | 0.09 | 0.038 | 0.038 | 0.181 | 0.181 | 1.021 | 1.021 | 0.179 | 0.179 | 0.037 | 0.037 | 0.012 | 0.012 | 0.006 |
| 6 | 0.003 | 0.015 | 0.05 | 0.095 | 0.898 | −5.056 | 0.851 | 0.219 | 0.197 | 0.197 | 1.038 | 1.038 | 0.176 | 0.176 | 0.038 | 0.038 | 0.011 | 0.011 | 0.001 | 0.001 | 0.002 |
| 7 | 0.001 | 0.003 | 0.013 | 0.048 | 0.085 | 0.851 | −4.498 | 1.025 | 1.009 | 1.009 | 0.181 | 0.181 | 0.034 | 0.034 | 0.011 | 0.011 | 0.001 | 0.001 | 0.000 | 0.000 | 0.001 |
| 8 | 0.001 | 0.002 | 0.006 | 0.025 | 0.09 | 0.219 | 1.025 | −1.987 | 0.288 | 0.288 | −0.003 | −0.003 | 0.021 | 0.021 | 0.002 | 0.002 | 0.001 | 0.001 | 0.000 | 0.000 | 0.000 |
| 9 | 0.000 | 0.000 | 0.002 | 0.011 | 0.038 | 0.197 | 1.009 | 0.288 | −1.98 | 0.295 | 0.002 | 0.153 | −0.001 | −0.026 | 0.002 | 0.009 | 0.000 | 0.000 | 0.000 | 0.000 | 0.000 |
| 10 | 0.000 | 0.000 | 0.002 | 0.011 | 0.038 | 0.197 | 1.009 | 0.288 | 0.295 | −1.98 | 0.153 | 0.002 | −0.026 | −0.001 | 0.009 | 0.002 | 0.000 | 0.000 | 0.000 | 0.000 | 0.000 |
| 11 | 0.001 | 0.001 | 0.011 | 0.037 | 0.181 | 1.038 | 0.181 | −0.003 | 0.002 | 0.153 | −2.003 | 0.27 | 0.149 | 0.003 | −0.03 | −0.001 | 0.002 | 0.009 | 0.000 | 0.000 | 0.000 |
| 12 | 0.001 | 0.001 | 0.011 | 0.037 | 0.181 | 1.038 | 0.181 | −0.003 | 0.153 | 0.002 | 0.27 | −2.003 | 0.003 | 0.149 | −0.001 | −0.03 | 0.009 | 0.002 | 0.000 | 0.000 | 0.000 |
| 13 | 0.002 | 0.011 | 0.036 | 0.176 | 1.021 | 0.176 | 0.034 | 0.021 | −0.001 | −0.026 | 0.149 | 0.003 | −2.002 | 0.27 | 0.146 | 0.003 | −0.001 | −0.03 | 0.009 | 0.002 | 0.001 |
| 14 | 0.002 | 0.011 | 0.036 | 0.176 | 1.021 | 0.176 | 0.034 | 0.021 | −0.026 | −0.001 | 0.003 | 0.149 | 0.27 | −2.002 | 0.003 | 0.146 | −0.03 | −0.001 | 0.002 | 0.009 | 0.001 |
| 15 | 0.02 | 0.033 | 0.179 | 1.023 | 0.179 | 0.038 | 0.011 | 0.002 | 0.002 | 0.009 | −0.03 | −0.001 | 0.146 | 0.003 | −2.004 | 0.272 | 0.003 | 0.145 | −0.031 | 0.000 | 0.003 |
| 16 | 0.02 | 0.033 | 0.179 | 1.023 | 0.179 | 0.038 | 0.011 | 0.002 | 0.009 | 0.002 | −0.001 | −0.03 | 0.003 | 0.146 | 0.272 | −2.004 | 0.145 | 0.003 | 0.000 | −0.031 | 0.003 |
| 17 | 0.027 | 0.173 | 1.023 | 0.176 | 0.037 | 0.011 | 0.001 | 0.001 | 0.000 | 0.000 | 0.002 | 0.009 | −0.001 | −0.03 | 0.003 | 0.145 | −2.01 | 0.266 | 0.001 | 0.145 | 0.021 |
| 18 | 0.027 | 0.173 | 1.023 | 0.176 | 0.037 | 0.011 | 0.001 | 0.001 | 0.000 | 0.000 | 0.009 | 0.002 | −0.03 | −0.001 | 0.145 | 0.003 | 0.266 | −2.01 | 0.145 | 0.001 | 0.021 |
| 19 | 0.341 | 0.991 | 0.179 | 0.035 | 0.012 | 0.001 | 0.000 | 0.000 | 0.000 | 0.000 | 0.000 | 0.000 | 0.009 | 0.002 | −0.031 | 0.000 | 0.001 | 0.145 | −1.962 | 0.27 | 0.008 |
| 20 | 0.341 | 0.991 | 0.179 | 0.035 | 0.012 | 0.001 | 0.000 | 0.000 | 0.000 | 0.000 | 0.000 | 0.000 | 0.002 | 0.009 | 0.000 | −0.031 | 0.145 | 0.001 | 0.27 | −1.962 | 0.008 |
| 21 | 1.537 | 0.242 | 0.079 | 0.027 | 0.006 | 0.002 | 0.001 | 0.000 | 0.000 | 0.000 | 0.000 | 0.000 | 0.001 | 0.001 | 0.003 | 0.003 | 0.021 | 0.021 | 0.008 | 0.008 | −1.959 |

**Table S2**. All LRF−D values of hexa-1,3,5-trien-1-ol at the B3LYP/6-311G** level. The numbering of the atoms listed is the same as presented in Figure 5b in the text.

| | **1** | **2** | **3** | **4** | **5** | **6** | **7** | **8** | **9** | **10** | **11** | **12** | **13** | **14** | **15** |
|---|---|---|---|---|---|---|---|---|---|---|---|---|---|---|---|
| 1 | −4.242 | 0.831 | 0.799 | 0.081 | 0.367 | 0.035 | 0.190 | 0.040 | 0.034 | 0.003 | 0.056 | 0.017 | 0.155 | 0.239 | 1.397 |
| 2 | 0.831 | −7.088 | 2.847 | 0.135 | 1.013 | 0.086 | 0.502 | 0.103 | 0.089 | 0.008 | 0.153 | 0.029 | 0.569 | 0.521 | 0.203 |
| 3 | 0.799 | 2.847 | −6.548 | 0.821 | 0.203 | 0.070 | 0.083 | 0.021 | 0.016 | 0.012 | 0.071 | 0.206 | 0.645 | 0.613 | 0.142 |
| 4 | 0.081 | 0.135 | 0.821 | −7.012 | 2.865 | 0.221 | 1.021 | 0.217 | 0.183 | 0.050 | 0.554 | 0.597 | 0.191 | 0.053 | 0.024 |
| 5 | 0.367 | 1.013 | 0.203 | 2.865 | −7.035 | 0.786 | 0.069 | 0.075 | 0.057 | 0.199 | 0.582 | 0.557 | 0.046 | 0.169 | 0.048 |
| 6 | 0.035 | 0.086 | 0.070 | 0.221 | 0.786 | −6.595 | 3.063 | 0.684 | 0.685 | 0.668 | 0.190 | 0.076 | 0.013 | 0.013 | 0.005 |
| 7 | 0.190 | 0.502 | 0.083 | 1.021 | 0.069 | 3.063 | −6.829 | 0.521 | 0.517 | 0.574 | 0.026 | 0.150 | 0.007 | 0.082 | 0.024 |
| 8 | 0.040 | 0.103 | 0.021 | 0.217 | 0.075 | 0.684 | 0.521 | −1.990 | 0.154 | 0.104 | 0.018 | 0.031 | 0.002 | 0.016 | 0.005 |
| 9 | 0.034 | 0.089 | 0.016 | 0.183 | 0.057 | 0.685 | 0.517 | 0.154 | −1.993 | 0.238 | −0.033 | 0.032 | 0.002 | 0.015 | 0.004 |
| 10 | 0.003 | 0.008 | 0.012 | 0.050 | 0.199 | 0.668 | 0.574 | 0.104 | 0.238 | −1.972 | 0.143 | −0.041 | 0.009 | 0.002 | 0.000 |
| 11 | 0.056 | 0.153 | 0.071 | 0.554 | 0.582 | 0.190 | 0.026 | 0.018 | −0.033 | 0.143 | −1.994 | 0.228 | −0.031 | 0.029 | 0.008 |
| 12 | 0.017 | 0.029 | 0.206 | 0.597 | 0.557 | 0.076 | 0.150 | 0.031 | 0.032 | −0.041 | 0.228 | −1.983 | 0.139 | −0.040 | 0.003 |
| 13 | 0.155 | 0.569 | 0.645 | 0.191 | 0.046 | 0.013 | 0.007 | 0.002 | 0.002 | 0.009 | −0.031 | 0.139 | −2.015 | 0.223 | 0.044 |
| 14 | 0.239 | 0.521 | 0.613 | 0.053 | 0.169 | 0.013 | 0.082 | 0.016 | 0.015 | 0.002 | 0.029 | −0.040 | 0.223 | −1.915 | −0.021 |
| 15 | 1.397 | 0.203 | 0.142 | 0.024 | 0.048 | 0.005 | 0.024 | 0.005 | 0.004 | 0.000 | 0.008 | 0.003 | 0.044 | −0.021 | −1.886 |

**Table S3.** $\pi$ contributions of LRF-D values of hexa-1,3,5-trien-1-ol at the B3LYP/6-311G** level. The numbering of the atoms listed is the same as presented in Figure 5b in the text.

| | **1** | **2** | **3** | **4** | **5** | **6** | **7** | **8** | **9** | **10** | **11** | **12** | **13** | **14** | **15** |
|---|---|---|---|---|---|---|---|---|---|---|---|---|---|---|---|
| 1 | −1.45 | −0.108 | 0.698 | 0.032 | 0.358 | 0.034 | 0.19 | 0.04 | 0.034 | 0.004 | 0.054 | −0.002 | 0.128 | −0.062 | 0.054 |
| 2 | −0.108 | −3.86 | 2.188 | 0.018 | 0.984 | 0.078 | 0.5 | 0.102 | 0.088 | 0.008 | 0.142 | −0.008 | 0.402 | −0.512 | −0.02 |
| 3 | 0.698 | 2.188 | −3.272 | −0.02 | 0.1 | 0.026 | 0.076 | 0.018 | 0.014 | 0.002 | 0.02 | 0.036 | −0.43 | 0.46 | 0.084 |
| 4 | 0.032 | 0.018 | −0.02 | −3.676 | 2.188 | 0.114 | 0.994 | 0.202 | 0.174 | 0.006 | 0.398 | −0.47 | 0.028 | 0.006 | 0.004 |
| 5 | 0.358 | 0.984 | 0.1 | 2.188 | −3.698 | −0.06 | −0.024 | −0.004 | 0.002 | 0.028 | −0.484 | 0.404 | 0.004 | 0.158 | 0.044 |
| 6 | 0.034 | 0.078 | 0.026 | 0.114 | −0.06 | −3.268 | 2.43 | 0.49 | 0.512 | −0.416 | 0.024 | 0.018 | 0.004 | 0.012 | 0.004 |
| 7 | 0.19 | 0.5 | 0.076 | 0.994 | −0.024 | 2.43 | −3.756 | −0.54 | −0.528 | 0.418 | −0.012 | 0.14 | 0.006 | 0.082 | 0.024 |
| 8 | 0.04 | 0.102 | 0.018 | 0.202 | −0.004 | 0.49 | −0.54 | −0.282 | −0.152 | 0.08 | −0.002 | 0.028 | 0.002 | 0.016 | 0.004 |
| 9 | 0.034 | 0.088 | 0.014 | 0.174 | 0.002 | 0.512 | −0.528 | −0.152 | −0.28 | 0.096 | −0.004 | 0.02 | 0.002 | 0.014 | 0.004 |
| 10 | 0.004 | 0.008 | 0.002 | 0.006 | 0.028 | −0.416 | 0.418 | 0.08 | 0.096 | −0.24 | 0.016 | −0.004 | 0.000 | 0.002 | 0.000 |
| 11 | 0.054 | 0.142 | 0.02 | 0.398 | −0.484 | 0.024 | −0.012 | −0.002 | −0.004 | 0.016 | −0.272 | 0.092 | 0.000 | 0.02 | 0.006 |
| 12 | −0.002 | −0.008 | 0.036 | −0.47 | 0.404 | 0.018 | 0.14 | 0.028 | 0.02 | −0.004 | 0.092 | −0.262 | 0.018 | −0.006 | 0.000 |
| 13 | 0.128 | 0.402 | −0.43 | 0.028 | 0.004 | 0.004 | 0.006 | 0.002 | 0.002 | 0.000 | 0.000 | 0.018 | −0.272 | 0.098 | 0.016 |
| 14 | −0.062 | −0.512 | 0.46 | 0.006 | 0.158 | 0.012 | 0.082 | 0.016 | 0.014 | 0.002 | 0.02 | −0.006 | 0.098 | −0.268 | −0.018 |
| 15 | 0.054 | −0.02 | 0.084 | 0.004 | 0.044 | 0.004 | 0.024 | 0.004 | 0.004 | 0.000 | 0.006 | 0.000 | 0.016 | −0.018 | −0.208 |

**Table S4.** The LRF-BO values, $\left\{\delta B^{IJ}/\delta v(K)\right\}$, of hexan-1-ol at the B3LYP/6-311G** level. The numbering of the atoms is the same as presented in Figure 5a in the text.

| **IJ of $\delta B^{IJ}$** | | **The Site Which the Perturbation Is Applied to (K of $\delta v(K)$)** | | | | | | | | | | | | | | | | | | | | | |
|---|---|---|---|---|---|---|---|---|---|---|---|---|---|---|---|---|---|---|---|---|---|---|---|
| I | J | 1 | 2 | 3 | 4 | 5 | 6 | 7 | 8 | 9 | 10 | 11 | 12 | 13 | 14 | 15 | 16 | 17 | 18 | 19 | 20 | 21 | 22 |
| 1 | 2 | 2.440 | −1.186 | −0.135 | −0.049 | −0.017 | −0.004 | −0.001 | 0.000 | 0.000 | 0.000 | −0.001 | −0.001 | −0.003 | −0.003 | −0.024 | −0.024 | −0.006 | −0.006 | −0.632 | −0.632 | 0.283 | 0.023 |
| 2 | 3 | −0.057 | 0.064 | 0.029 | 0.052 | −0.008 | −0.006 | −0.002 | 0.001 | −0.001 | −0.001 | 0.002 | 0.002 | −0.009 | −0.009 | 0.086 | 0.086 | −0.103 | −0.103 | 0.013 | 0.013 | −0.048 | 0.023 |
| 3 | 4 | −0.061 | 0.055 | −0.058 | 0.089 | 0.042 | −0.006 | −0.006 | −0.015 | 0.003 | 0.003 | −0.009 | −0.009 | 0.098 | 0.098 | −0.107 | −0.107 | −0.118 | −0.118 | 0.116 | 0.116 | −0.002 | 0.030 |
| 4 | 5 | 0.002 | −0.006 | 0.040 | 0.068 | −0.002 | 0.041 | −0.009 | −0.044 | −0.022 | −0.022 | 0.104 | 0.104 | −0.105 | −0.105 | −0.106 | −0.106 | 0.100 | 0.100 | −0.007 | −0.007 | −0.019 | 0.030 |
| 5 | 6 | −0.004 | −0.008 | −0.008 | 0.029 | 0.043 | 0.060 | 0.065 | −0.176 | 0.089 | 0.089 | −0.027 | −0.027 | −0.145 | −0.145 | 0.089 | 0.089 | −0.009 | −0.009 | 0.004 | 0.004 | −0.004 | 0.237 |
| 6 | 7 | 0.000 | −0.003 | −0.008 | −0.012 | −0.012 | 0.037 | −0.067 | 0.169 | 0.019 | 0.019 | −0.147 | −0.147 | 0.086 | 0.086 | −0.011 | −0.011 | 0.001 | 0.001 | 0.000 | 0.000 | −0.001 | 0.237 |
| 1 | 21 | 1.400 | 0.144 | 0.037 | 0.011 | 0.002 | 0.001 | 0.000 | 0.000 | 0.000 | 0.000 | 0.000 | 0.000 | 0.000 | 0.000 | 0.003 | 0.003 | 0.011 | 0.011 | 0.062 | 0.062 | −1.748 | 0.003 |
| 2 | 19 | −0.224 | 0.438 | 0.029 | 0.002 | 0.001 | 0.000 | 0.000 | 0.000 | 0.000 | 0.000 | −0.001 | 0.000 | 0.002 | 0.001 | 0.014 | 0.000 | 0.051 | 0.003 | −0.380 | 0.056 | 0.007 | 0.001 |
| 2 | 20 | −0.224 | 0.438 | 0.029 | 0.002 | 0.001 | 0.000 | 0.000 | 0.000 | 0.000 | 0.000 | 0.000 | −0.001 | 0.001 | 0.002 | 0.000 | 0.014 | 0.003 | 0.051 | 0.056 | −0.380 | 0.007 | 0.001 |
| 3 | 17 | −0.003 | 0.043 | 0.564 | 0.036 | 0.004 | 0.002 | 0.000 | 0.001 | 0.000 | 0.000 | 0.001 | 0.001 | 0.000 | 0.006 | 0.029 | 0.005 | −0.762 | 0.067 | 0.029 | −0.011 | −0.013 | 0.004 |
| 3 | 18 | −0.003 | 0.043 | 0.564 | 0.036 | 0.004 | 0.002 | 0.000 | 0.001 | 0.000 | 0.000 | 0.001 | 0.001 | 0.006 | 0.000 | 0.005 | 0.029 | 0.067 | −0.762 | −0.011 | 0.029 | −0.013 | 0.004 |
| 4 | 16 | −0.006 | 0.003 | 0.031 | 0.479 | 0.029 | 0.001 | 0.001 | 0.004 | 0.000 | 0.002 | 0.000 | 0.004 | 0.026 | −0.007 | 0.040 | −0.634 | 0.000 | 0.023 | 0.001 | 0.003 | 0.001 | 0.003 |
| 7 | 8 | 0.000 | 0.000 | 0.000 | −0.001 | 0.017 | 0.050 | 0.440 | −0.702 | 0.034 | 0.034 | 0.055 | 0.055 | 0.007 | 0.007 | 0.001 | 0.001 | 0.000 | 0.000 | 0.000 | 0.000 | 0.000 | 0.755 |
| 7 | 9 | 0.000 | 0.000 | 0.000 | 0.002 | 0.005 | 0.043 | 0.455 | 0.041 | −0.649 | 0.051 | 0.047 | −0.013 | 0.004 | 0.012 | 0.002 | 0.002 | −0.001 | 0.000 | 0.000 | 0.000 | 0.000 | 0.113 |
| 7 | 10 | 0.000 | 0.000 | 0.000 | 0.002 | 0.005 | 0.043 | 0.455 | 0.041 | 0.051 | −0.649 | −0.013 | 0.047 | 0.012 | 0.004 | 0.002 | 0.002 | 0.000 | −0.001 | 0.000 | 0.000 | 0.000 | 0.014 |
| 4 | 15 | −0.006 | 0.003 | 0.031 | 0.479 | 0.029 | 0.001 | 0.001 | 0.004 | 0.002 | 0.000 | 0.004 | 0.000 | −0.007 | 0.026 | −0.634 | 0.040 | 0.023 | 0.000 | 0.003 | 0.001 | 0.001 | 0.013 |
| 5 | 13 | 0.001 | 0.001 | 0.003 | 0.030 | 0.490 | 0.029 | 0.002 | −0.004 | 0.000 | 0.002 | 0.003 | 0.020 | −0.644 | 0.039 | −0.003 | 0.029 | 0.000 | 0.001 | 0.000 | 0.002 | 0.001 | 0.002 |
| 5 | 14 | 0.001 | 0.001 | 0.003 | 0.030 | 0.490 | 0.029 | 0.002 | −0.004 | 0.002 | 0.000 | 0.020 | 0.003 | 0.039 | −0.644 | 0.029 | −0.003 | 0.001 | 0.000 | 0.002 | 0.000 | 0.001 | 0.002 |
| 6 | 11 | 0.000 | 0.000 | 0.002 | 0.003 | 0.031 | 0.486 | 0.029 | 0.022 | 0.005 | 0.015 | −0.669 | 0.035 | −0.005 | 0.033 | 0.009 | 0.001 | 0.002 | 0.001 | −0.001 | 0.000 | 0.000 | 0.001 |
| 6 | 12 | 0.000 | 0.000 | 0.002 | 0.003 | 0.031 | 0.486 | 0.029 | 0.022 | 0.015 | 0.005 | 0.035 | −0.669 | 0.033 | −0.005 | 0.001 | 0.009 | 0.001 | 0.002 | 0.000 | −0.001 | 0.000 | 0.002 |

**Table S5.** LRF-BO values, $\left\{\delta B^{IJ}/\delta v(K)\right\}$, of hexa-1,3,5-trien-1-ol at the B3LYP/6-311G** level. The numbering of the atoms is the same as presented in Figure 5b in the text.

| IJ of $\delta B^{IJ}$ | | The Site Which the Perturbation Is Applied to (K of $\delta v(K)$) | | | | | | | | | | | | | | |
|---|---|---|---|---|---|---|---|---|---|---|---|---|---|---|---|---|
| I | J | 1 | 2 | 3 | 4 | 5 | 6 | 7 | 8 | 9 | 10 | 11 | 12 | 13 | 14 | 15 |
| 1 | 2 | 3.285 | −0.589 | −1.381 | −0.070 | −0.550 | −0.047 | −0.281 | −0.056 | −0.050 | −0.004 | −0.081 | −0.014 | −0.237 | −0.219 | 0.293 |
| 2 | 3 | −1.072 | −0.396 | 0.612 | 0.285 | 0.388 | 0.060 | 0.232 | 0.065 | 0.049 | 0.010 | 0.022 | 0.122 | 0.013 | 0.041 | −0.432 |
| 3 | 4 | 0.121 | 0.456 | 0.091 | −0.171 | −0.164 | −0.078 | −0.142 | −0.031 | −0.010 | −0.083 | 0.000 | −0.027 | −0.042 | 0.088 | −0.008 |
| 4 | 5 | −0.165 | −0.245 | −0.003 | −0.013 | 0.365 | 0.347 | 0.131 | 0.056 | −0.043 | 0.097 | −0.179 | −0.230 | −0.004 | −0.078 | −0.034 |
| 5 | 6 | 0.064 | 0.107 | 0.020 | 0.324 | −0.145 | −0.023 | 0.140 | −0.298 | −0.131 | −0.063 | −0.098 | 0.128 | −0.060 | 0.024 | 0.010 |
| 6 | 7 | −0.057 | −0.090 | −0.048 | −0.187 | 0.046 | 0.030 | 0.446 | 0.160 | −0.002 | −0.287 | 0.046 | −0.038 | −0.003 | −0.010 | −0.006 |
| 1 | 15 | 1.397 | 0.169 | 0.062 | 0.010 | 0.019 | 0.002 | 0.010 | 0.002 | 0.002 | 0.000 | 0.003 | 0.003 | 0.020 | 0.075 | −1.774 |
| 2 | 14 | −0.098 | 0.371 | 0.030 | −0.008 | 0.022 | 0.002 | 0.010 | 0.002 | 0.002 | 0.002 | 0.004 | −0.035 | −0.036 | −0.494 | 0.227 |
| 3 | 13 | 0.060 | 0.126 | 0.437 | 0.022 | 0.002 | 0.003 | 0.000 | 0.000 | 0.001 | −0.003 | 0.022 | −0.031 | −0.590 | −0.030 | −0.017 |
| 4 | 12 | −0.004 | −0.001 | 0.004 | 0.346 | 0.063 | 0.000 | 0.021 | 0.004 | −0.005 | −0.007 | −0.032 | −0.394 | −0.034 | 0.039 | 0.000 |
| 5 | 11 | 0.006 | 0.021 | 0.001 | 0.066 | 0.350 | 0.005 | 0.001 | −0.009 | 0.053 | −0.038 | −0.412 | −0.026 | −0.017 | −0.003 | 0.002 |
| 6 | 10 | 0.000 | −0.001 | 0.002 | 0.000 | −0.009 | 0.327 | 0.053 | 0.106 | 0.013 | −0.472 | −0.029 | 0.007 | 0.001 | 0.001 | 0.000 |
| 7 | 8 | 0.004 | 0.012 | 0.003 | 0.025 | 0.016 | 0.108 | 0.328 | −0.766 | 0.130 | 0.115 | 0.009 | 0.012 | 0.002 | 0.002 | 0.001 |
| 7 | 9 | 0.004 | 0.010 | 0.002 | 0.023 | −0.006 | 0.064 | 0.379 | 0.181 | −0.629 | −0.022 | −0.021 | 0.010 | 0.003 | 0.001 | 0.000 |

**Table S6.** $\pi$ contributions of LRF-BO values, $\left\{\delta B^{IJ}/\delta v(K)\right\}$, of hexa-1,3,5-trien-1-ol at the B3LYP/6-311G** level. The numbering of the atom pair listed is the same as presented in Figure 5b in the text.

| IJ of $\delta B^{IJ}$ | | The Site Which the Perturbation Is Applied to (K of $\delta v(K)$) | | | | | | | | | | | | | | |
|---|---|---|---|---|---|---|---|---|---|---|---|---|---|---|---|---|
| I | J | 1 | 2 | 3 | 4 | 5 | 6 | 7 | 8 | 9 | 10 | 11 | 12 | 13 | 14 | 15 |
| 1 | 2 | 1.605 | 0.639 | −1.275 | −0.041 | −0.544 | −0.045 | −0.280 | −0.057 | −0.050 | −0.005 | −0.076 | 0.010 | −0.292 | 0.208 | 0.203 |
| 2 | 3 | −1.079 | −0.610 | 0.585 | 0.199 | 0.401 | 0.067 | 0.233 | 0.059 | 0.047 | −0.001 | 0.134 | −0.046 | 0.095 | 0.016 | −0.101 |
| 3 | 4 | 0.197 | 0.328 | 0.031 | −0.112 | −0.225 | −0.054 | −0.131 | −0.037 | −0.027 | 0.013 | −0.161 | 0.097 | 0.135 | −0.073 | 0.018 |
| 4 | 5 | −0.169 | −0.229 | −0.131 | −0.236 | 0.207 | 0.205 | 0.168 | 0.087 | 0.119 | −0.044 | 0.092 | 0.026 | −0.131 | 0.055 | −0.020 |
| 5 | 6 | 0.059 | 0.109 | 0.031 | 0.258 | −0.126 | −0.202 | −0.039 | −0.084 | −0.147 | 0.074 | 0.092 | −0.064 | 0.018 | 0.013 | 0.007 |
| 6 | 7 | −0.059 | −0.090 | −0.044 | −0.175 | 0.013 | −0.197 | 0.263 | 0.184 | 0.239 | −0.078 | −0.087 | 0.059 | −0.011 | −0.010 | −0.007 |
| 1 | 15 | 0.177 | 0.016 | 0.034 | 0.002 | 0.018 | 0.002 | 0.009 | 0.002 | 0.002 | 0.000 | 0.003 | 0.000 | 0.006 | −0.004 | −0.266 |
| 2 | 14 | −0.006 | −0.034 | 0.058 | 0.000 | 0.020 | 0.001 | 0.010 | 0.002 | 0.002 | 0.000 | 0.003 | 0.000 | 0.009 | −0.066 | 0.000 |
| 3 | 13 | 0.015 | 0.056 | −0.019 | 0.007 | −0.002 | 0.000 | 0.000 | 0.000 | 0.000 | 0.000 | 0.000 | 0.001 | −0.068 | 0.009 | 0.002 |
| 4 | 12 | −0.001 | −0.004 | 0.006 | −0.026 | 0.052 | 0.002 | 0.017 | 0.003 | 0.003 | 0.000 | 0.008 | −0.061 | 0.000 | 0.000 | 0.000 |
| 5 | 11 | 0.007 | 0.018 | 0.002 | 0.052 | −0.026 | 0.006 | −0.004 | −0.001 | 0.000 | 0.001 | −0.065 | 0.008 | 0.001 | 0.003 | 0.001 |
| 6 | 10 | 0.000 | −0.001 | 0.000 | −0.002 | 0.006 | −0.020 | 0.054 | 0.009 | 0.009 | −0.056 | 0.001 | 0.000 | 0.000 | 0.000 | 0.000 |
| 7 | 8 | 0.004 | 0.011 | 0.002 | 0.022 | −0.001 | 0.062 | −0.037 | −0.074 | −0.003 | 0.008 | 0.000 | 0.003 | 0.000 | 0.002 | 0.001 |
| 7 | 9 | 0.004 | 0.010 | 0.002 | 0.020 | 0.000 | 0.062 | −0.036 | −0.003 | −0.072 | 0.009 | 0.000 | 0.003 | 0.000 | 0.002 | 0.000 |

**Table S7.** All LRF-BO values, $\left\{\delta B^{O\text{-}H}/\delta v(L)\right\}_{L}^{All\ atoms}$, of meta-substituted benzoic acids at the B3LYP/6-311G** level. The numbering of the atoms listed in the top row is the same as presented in Figure 9 in the text.

| | O(1) | C(2) | O(3) | H(4) | C(5) | C(6) | C(7) | C(8) | C(9) | C(10) | H(11) | H(13) | H(15) |
|---|---|---|---|---|---|---|---|---|---|---|---|---|---|
| H | 0.00270 | 0.04773 | 0.37158 | −0.44517 | 0.00774 | 0.00141 | 0.00027 | 0.00080 | 0.00076 | 0.00282 | 0.00823 | 0.00020 | 0.00052 |
| $C_6H_5$ | 0.00291 | 0.04775 | 0.37033 | −0.44465 | 0.00772 | 0.00140 | 0.00030 | 0.00079 | 0.00078 | 0.00283 | 0.00870 | 0.00018 | 0.00049 |
| Br | 0.00356 | 0.04798 | 0.37153 | −0.44643 | 0.00791 | 0.00142 | 0.00029 | 0.00078 | 0.00076 | 0.00283 | 0.00835 | 0.00017 | 0.00050 |
| Cl | 0.00360 | 0.04796 | 0.37162 | −0.44650 | 0.00791 | 0.00141 | 0.00028 | 0.00078 | 0.00076 | 0.00283 | 0.00830 | 0.00017 | 0.00051 |
| CN | 0.00443 | 0.04816 | 0.37188 | −0.44798 | 0.00800 | 0.00138 | 0.00029 | 0.00078 | 0.00079 | 0.00289 | 0.00826 | 0.00016 | 0.00053 |
| $COCH_3$ | 0.00390 | 0.04831 | 0.36953 | −0.44478 | 0.00795 | 0.00129 | 0.00026 | 0.00077 | 0.00080 | 0.00293 | 0.00789 | 0.00015 | 0.00052 |
| $COOC_2H_5$ | 0.00376 | 0.04818 | 0.36973 | −0.44476 | 0.00791 | 0.00131 | 0.00027 | 0.00081 | 0.00081 | 0.00294 | 0.00795 | 0.00015 | 0.00052 |
| COOH | 0.00391 | 0.04820 | 0.37010 | −0.44535 | 0.00795 | 0.00132 | 0.00027 | 0.00081 | 0.00080 | 0.00295 | 0.00796 | 0.00015 | 0.00052 |
| $C(CH_3)_3$ | 0.00252 | 0.04759 | 0.36945 | −0.44387 | 0.00764 | 0.00143 | 0.00029 | 0.00077 | 0.00078 | 0.00279 | 0.00941 | 0.00016 | 0.00049 |
| $CH_3CH_3$ | 0.00259 | 0.04770 | 0.37046 | −0.44429 | 0.00772 | 0.00142 | 0.00029 | 0.00080 | 0.00078 | 0.00283 | 0.00860 | 0.00018 | 0.00049 |
| F | 0.00334 | 0.04791 | 0.37173 | −0.44614 | 0.00793 | 0.00150 | 0.00031 | 0.00079 | 0.00075 | 0.00280 | 0.00801 | 0.00018 | 0.00047 |
| I | 0.00358 | 0.04796 | 0.37144 | −0.44644 | 0.00789 | 0.00141 | 0.00029 | 0.00077 | 0.00076 | 0.00283 | 0.00850 | 0.00016 | 0.00050 |
| $IO_2$ | 0.00457 | 0.04820 | 0.37179 | −0.44794 | 0.00806 | 0.00137 | 0.00026 | 0.00078 | 0.00078 | 0.00291 | 0.00812 | 0.00016 | 0.00054 |
| $CH_3$ | 0.00257 | 0.04768 | 0.37058 | −0.44427 | 0.00768 | 0.00140 | 0.00028 | 0.00081 | 0.00078 | 0.00283 | 0.00855 | 0.00019 | 0.00050 |
| $O(CH_2)_3CH_3$ | 0.00269 | 0.04784 | 0.37011 | −0.44376 | 0.00779 | 0.00157 | 0.00032 | 0.00073 | 0.00075 | 0.00273 | 0.00815 | 0.00015 | 0.00043 |
| $O(CH_3)_4CH_3$ | 0.00268 | 0.04784 | 0.37011 | −0.44374 | 0.00779 | 0.00157 | 0.00032 | 0.00073 | 0.00075 | 0.00273 | 0.00814 | 0.00015 | 0.00043 |
| $O(CH_2)_2CH_3$ | 0.00270 | 0.04784 | 0.37013 | −0.44379 | 0.00779 | 0.00157 | 0.00032 | 0.00073 | 0.00075 | 0.00273 | 0.00815 | 0.00015 | 0.00043 |
| $NH_2$ | 0.00228 | 0.04754 | 0.37053 | −0.44394 | 0.00766 | 0.00154 | 0.00032 | 0.00072 | 0.00075 | 0.00271 | 0.00881 | 0.00016 | 0.00043 |
| $NHCOCH_3$ | 0.00269 | 0.04752 | 0.37157 | −0.44586 | 0.00775 | 0.00154 | 0.00031 | 0.00077 | 0.00076 | 0.00277 | 0.00909 | 0.00015 | 0.00043 |
| $NO_2$ | 0.00456 | 0.04829 | 0.37131 | −0.44761 | 0.00816 | 0.00140 | 0.00030 | 0.00081 | 0.00081 | 0.00296 | 0.00798 | 0.00014 | 0.00047 |
| $OC_6H_5$ | 0.00302 | 0.04785 | 0.37067 | −0.44469 | 0.00782 | 0.00149 | 0.00032 | 0.00078 | 0.00076 | 0.00280 | 0.00809 | 0.00017 | 0.00046 |
| $OCH_2CH_3$ | 0.00241 | 0.04763 | 0.36996 | −0.44415 | 0.00768 | 0.00141 | 0.00030 | 0.00079 | 0.00077 | 0.00281 | 0.00928 | 0.00017 | 0.00048 |
| OH | 0.00254 | 0.04752 | 0.37153 | −0.44539 | 0.00773 | 0.00156 | 0.00033 | 0.00078 | 0.00075 | 0.00275 | 0.00883 | 0.00018 | 0.00044 |
| $OCH(CH_3)_2$ | 0.00229 | 0.04751 | 0.36979 | −0.44404 | 0.00765 | 0.00143 | 0.00030 | 0.00079 | 0.00076 | 0.00278 | 0.00961 | 0.00017 | 0.00047 |
| $OCH_3$ | 0.00244 | 0.04760 | 0.37020 | −0.44438 | 0.00769 | 0.00143 | 0.00030 | 0.00079 | 0.00076 | 0.00281 | 0.00928 | 0.00017 | 0.00047 |
| SH | 0.00323 | 0.04788 | 0.37097 | −0.44552 | 0.00780 | 0.00141 | 0.00027 | 0.00073 | 0.00074 | 0.00277 | 0.00864 | 0.00018 | 0.00050 |
| $Si(CH_3)_3$ | 0.00256 | 0.04768 | 0.36984 | −0.44389 | 0.00767 | 0.00135 | 0.00026 | 0.00078 | 0.00078 | 0.00285 | 0.00898 | 0.00017 | 0.00053 |
| $SCH_3$ | 0.00318 | 0.04791 | 0.37103 | −0.44541 | 0.00785 | 0.00136 | 0.00028 | 0.00079 | 0.00077 | 0.00284 | 0.00834 | 0.00018 | 0.00051 |
| $SOCH_3$ | 0.00341 | 0.04770 | 0.37231 | −0.44716 | 0.00788 | 0.00149 | 0.00029 | 0.00078 | 0.00077 | 0.00283 | 0.00859 | 0.00017 | 0.00051 |
| $SO_2CH_3$ | 0.00432 | 0.04819 | 0.37123 | −0.44712 | 0.00804 | 0.00133 | 0.00027 | 0.00079 | 0.00078 | 0.00292 | 0.00816 | 0.00016 | 0.00054 |
| $SO_2NH_2$ | 0.00413 | 0.04816 | 0.37100 | −0.44668 | 0.00801 | 0.00133 | 0.00026 | 0.00079 | 0.00078 | 0.00291 | 0.00821 | 0.00016 | 0.00053 |
| $CF_3$ | 0.00405 | 0.04809 | 0.37129 | −0.44696 | 0.00795 | 0.00136 | 0.00027 | 0.00080 | 0.00079 | 0.00291 | 0.00836 | 0.00017 | 0.00052 |
| $OCOCH_3$ | 0.00361 | 0.04789 | 0.37190 | −0.44685 | 0.00793 | 0.00144 | 0.00030 | 0.00078 | 0.00076 | 0.00282 | 0.00831 | 0.00018 | 0.00047 |
| $SCOCH_3$ | 0.00368 | 0.04801 | 0.37175 | −0.44688 | 0.00794 | 0.00136 | 0.00028 | 0.00077 | 0.00076 | 0.00284 | 0.00840 | 0.00018 | 0.00052 |

**Table S8.** All LRF-BO values, $\left\{\delta B^{O\text{-}H}/\delta v(L)\right\}_{L}^{All\ atoms}$, of para-substituted benzoic acids at the B3LYP/6-311G** level. The numbering of the atoms listed in the top row is the same as presented in Figure 9 in the text.

| | O(1) | C(2) | O(3) | H(4) | C(5) | C(6) | C(7) | C(8) | C(9) | C(10) | H(11) | H(12) | H(14) | H(15) |
|---|---|---|---|---|---|---|---|---|---|---|---|---|---|---|
| H | 0.00270 | 0.04773 | 0.37158 | −0.44517 | 0.00774 | 0.00141 | 0.00027 | 0.00080 | 0.00076 | 0.00282 | 0.00823 | 0.0002 | 0.00047 | 0.00052 |
| $C_6H_5$ | 0.00272 | 0.04768 | 0.37153 | −0.44526 | 0.00773 | 0.00140 | 0.00026 | 0.00083 | 0.00077 | 0.00287 | 0.00818 | −0.00006 | 0.00041 | 0.00051 |
| Br | 0.00323 | 0.04783 | 0.37248 | −0.44692 | 0.00789 | 0.00138 | 0.00025 | 0.00082 | 0.00079 | 0.00287 | 0.00817 | −0.00010 | 0.00045 | 0.00055 |
| Cl | 0.00321 | 0.04781 | 0.37252 | −0.44695 | 0.00788 | 0.00139 | 0.00025 | 0.00082 | 0.00080 | 0.00287 | 0.00818 | −0.00010 | 0.00045 | 0.00054 |
| CN | 0.00424 | 0.04811 | 0.37281 | −0.44851 | 0.00796 | 0.00136 | 0.00028 | 0.00076 | 0.00072 | 0.00285 | 0.00815 | −0.00007 | 0.00044 | 0.00053 |
| $COCH_3$ | 0.00354 | 0.04792 | 0.37178 | −0.44653 | 0.00784 | 0.00142 | 0.00028 | 0.00080 | 0.00069 | 0.00282 | 0.00831 | −0.00004 | 0.00036 | 0.00050 |
| $CO_2C_2H_5$ | 0.00352 | 0.04791 | 0.37152 | −0.44610 | 0.00781 | 0.00139 | 0.00030 | 0.00080 | 0.00072 | 0.00285 | 0.00815 | −0.00003 | 0.00039 | 0.00050 |
| COOH | 0.00374 | 0.04797 | 0.37177 | −0.44663 | 0.00786 | 0.00138 | 0.00030 | 0.00078 | 0.00071 | 0.00286 | 0.00814 | −0.00003 | 0.00039 | 0.00050 |
| $C(CH_3)_3$ | 0.00248 | 0.04762 | 0.37120 | −0.44456 | 0.00776 | 0.00139 | 0.00026 | 0.00087 | 0.00080 | 0.00293 | 0.00816 | −0.00004 | 0.00038 | 0.00052 |
| $CH_2CH_3$ | 0.00245 | 0.04765 | 0.37143 | −0.44483 | 0.00774 | 0.00143 | 0.00026 | 0.00087 | 0.00080 | 0.00288 | 0.00820 | −0.00006 | 0.00043 | 0.00051 |
| F | 0.00282 | 0.04773 | 0.37258 | −0.44667 | 0.00789 | 0.00142 | 0.00026 | 0.00080 | 0.00084 | 0.00289 | 0.00824 | −0.00008 | 0.00044 | 0.00057 |
| I | 0.00328 | 0.04785 | 0.37243 | −0.44692 | 0.00788 | 0.00138 | 0.00025 | 0.00083 | 0.00078 | 0.00286 | 0.00816 | −0.00010 | 0.00045 | 0.00055 |
| $IO_2$ | 0.00422 | 0.04814 | 0.37280 | −0.44846 | 0.00802 | 0.00136 | 0.00028 | 0.00078 | 0.00075 | 0.00285 | 0.00821 | −0.00004 | 0.00042 | 0.00055 |
| $CH(CH_3)_2$ | 0.00248 | 0.04766 | 0.37133 | −0.44471 | 0.00774 | 0.00140 | 0.00025 | 0.00086 | 0.00079 | 0.00290 | 0.00821 | −0.00004 | 0.00042 | 0.00052 |
| $CH_3$ | 0.00244 | 0.04764 | 0.37149 | −0.44481 | 0.00772 | 0.00142 | 0.00026 | 0.00088 | 0.00080 | 0.00287 | 0.00817 | −0.00007 | 0.00044 | 0.00052 |
| SCN | 0.00335 | 0.04781 | 0.37255 | −0.44732 | 0.00784 | 0.00139 | 0.00025 | 0.00077 | 0.00077 | 0.00287 | 0.00819 | −0.00008 | 0.00043 | 0.00056 |
| $N(CH_3)_2$ | 0.00097 | 0.04709 | 0.37123 | −0.44340 | 0.00770 | 0.00141 | 0.00025 | 0.00075 | 0.00098 | 0.00296 | 0.00832 | −0.00005 | 0.00043 | 0.00048 |
| $NH_2$ | 0.00128 | 0.04719 | 0.37189 | −0.44429 | 0.00771 | 0.00140 | 0.00025 | 0.00083 | 0.00096 | 0.00293 | 0.00829 | −0.00007 | 0.00046 | 0.00053 |
| $NHCOCH_3$ | 0.00236 | 0.04768 | 0.37112 | −0.44477 | 0.00777 | 0.00140 | 0.00024 | 0.00078 | 0.00085 | 0.00291 | 0.00818 | −0.00005 | 0.00042 | 0.00053 |
| $NHCH_3$ | 0.00104 | 0.04707 | 0.37181 | −0.44395 | 0.00769 | 0.00135 | 0.00025 | 0.00080 | 0.00099 | 0.00300 | 0.00826 | −0.00006 | 0.00045 | 0.00053 |
| $NO_2$ | 0.00437 | 0.04816 | 0.37280 | −0.44870 | 0.00805 | 0.00138 | 0.00031 | 0.00073 | 0.00071 | 0.00286 | 0.00817 | −0.00003 | 0.00034 | 0.00055 |
| $OC_6H_5$ | 0.00209 | 0.04748 | 0.37193 | −0.44509 | 0.00777 | 0.00138 | 0.00025 | 0.00082 | 0.00089 | 0.00297 | 0.00818 | −0.00007 | 0.00042 | 0.00056 |
| $O(CH_2)_3CH_3$ | 0.00176 | 0.04740 | 0.37144 | −0.44451 | 0.00777 | 0.00151 | 0.00027 | 0.00082 | 0.00089 | 0.00286 | 0.00841 | −0.00006 | 0.00042 | 0.00050 |
| $OCOCH_3$ | 0.00269 | 0.04763 | 0.37218 | −0.44597 | 0.00783 | 0.00142 | 0.00027 | 0.00083 | 0.00081 | 0.00291 | 0.00820 | −0.00007 | 0.00040 | 0.00055 |
| $OCH_2CH_3$ | 0.00179 | 0.04741 | 0.37148 | −0.44460 | 0.00778 | 0.00151 | 0.00027 | 0.00082 | 0.00089 | 0.00286 | 0.00841 | −0.00006 | 0.00042 | 0.00050 |
| OH | 0.00202 | 0.04748 | 0.37198 | −0.44513 | 0.00776 | 0.00138 | 0.00024 | 0.00080 | 0.00091 | 0.00295 | 0.00819 | −0.00008 | 0.00045 | 0.00057 |
| $OCH(CH_3)_2$ | 0.00182 | 0.04740 | 0.37176 | −0.44464 | 0.00776 | 0.00136 | 0.00025 | 0.00081 | 0.00092 | 0.00301 | 0.00814 | −0.00007 | 0.00041 | 0.00056 |
| $OCH_3$ | 0.00196 | 0.04744 | 0.37184 | −0.44486 | 0.00776 | 0.00135 | 0.00025 | 0.00080 | 0.00092 | 0.00301 | 0.00814 | −0.00007 | 0.00043 | 0.00056 |
| $O(CH_3)_4CH_3$ | 0.00177 | 0.04740 | 0.37143 | −0.44449 | 0.00777 | 0.00151 | 0.00027 | 0.00082 | 0.00089 | 0.00286 | 0.00841 | −0.00006 | 0.00042 | 0.00050 |
| $O(CH_2)_2CH_3$ | 0.00178 | 0.04740 | 0.37147 | −0.44455 | 0.00777 | 0.00151 | 0.00027 | 0.00082 | 0.00089 | 0.00286 | 0.00841 | −0.00006 | 0.00042 | 0.00050 |
| $SCH(CH_3)_2$ | 0.00231 | 0.04750 | 0.37168 | −0.44522 | 0.00773 | 0.00142 | 0.00023 | 0.00084 | 0.00082 | 0.00285 | 0.00833 | −0.00006 | 0.00045 | 0.00050 |
| $SC_2H_5$ | 0.00234 | 0.04752 | 0.37177 | −0.44520 | 0.00774 | 0.00133 | 0.00022 | 0.00083 | 0.00085 | 0.00295 | 0.00814 | −0.00007 | 0.00043 | 0.00052 |
| SH | 0.00264 | 0.04762 | 0.37205 | −0.44583 | 0.00776 | 0.00136 | 0.00022 | 0.00083 | 0.00082 | 0.00288 | 0.00817 | −0.00009 | 0.00044 | 0.00054 |

**Table S8.** *Cont.*

| | O(1) | C(2) | O(3) | H(4) | C(5) | C(6) | C(7) | C(8) | C(9) | C(10) | H(11) | H(12) | H(14) | H(15) |
|---|---|---|---|---|---|---|---|---|---|---|---|---|---|---|
| $Si(CH_2CH_3)_3$ | 0.00274 | 0.04775 | 0.37103 | −0.44469 | 0.00774 | 0.00142 | 0.00026 | 0.00089 | 0.00076 | 0.00285 | 0.00822 | −0.00006 | 0.00043 | 0.00047 |
| $Si(CH_3)_3$ | 0.00271 | 0.04772 | 0.37118 | −0.44474 | 0.00773 | 0.00138 | 0.00026 | 0.00089 | 0.00076 | 0.00286 | 0.00819 | −0.00007 | 0.00045 | 0.00049 |
| $SCH_3$ | 0.00226 | 0.04751 | 0.37165 | −0.44520 | 0.00774 | 0.00145 | 0.00024 | 0.00083 | 0.00082 | 0.00283 | 0.00834 | −0.00006 | 0.00045 | 0.00050 |
| $SO_2CH_3$ | 0.00411 | 0.04809 | 0.37256 | −0.44801 | 0.00798 | 0.00138 | 0.00029 | 0.00078 | 0.00073 | 0.00284 | 0.00820 | −0.00004 | 0.00040 | 0.00052 |
| $SO_2NH_2$ | 0.00392 | 0.04804 | 0.37237 | −0.44758 | 0.00795 | 0.00138 | 0.00028 | 0.00080 | 0.00073 | 0.00285 | 0.00819 | −0.00005 | 0.00041 | 0.00052 |
| $SOCH_3$ | 0.00338 | 0.04785 | 0.37262 | −0.44713 | 0.00788 | 0.00137 | 0.00025 | 0.00083 | 0.00075 | 0.00286 | 0.00830 | −0.00005 | 0.00037 | 0.00051 |
| $CF_3$ | 0.00388 | 0.04803 | 0.37235 | −0.44752 | 0.00794 | 0.00139 | 0.00029 | 0.00079 | 0.00074 | 0.00286 | 0.00816 | −0.00004 | 0.00041 | 0.00052 |
| $SCOCH_3$ | 0.00311 | 0.04784 | 0.37163 | −0.44579 | 0.00779 | 0.00138 | 0.00026 | 0.00081 | 0.00076 | 0.00284 | 0.00816 | −0.00009 | 0.00045 | 0.00052 |

**Table S9.** All LRF-BO values, $\left\{\delta B^{O\text{-}H}/\delta v(L)\right\}_{L}^{All\ atoms}$, of meta-substituted benzoic acids at the B3LYP/6-31G level. The numbering of the atoms listed in the top row is the same as presented in Figure 9 in the text.

| | O(1) | C(2) | O(3) | H(4) | C(5) | C(6) | C(7) | C(8) | C(9) | C(10) | H(11) | H(13) | H(15) |
|---|---|---|---|---|---|---|---|---|---|---|---|---|---|
| H | −0.00541 | 0.02780 | 0.29570 | −0.33833 | 0.00156 | 0.00023 | 0.00010 | 0.00073 | 0.00254 | 0.00961 | 0.00552 | 0.00003 | 0.00029 |
| $C_6H_5$ | −0.00541 | 0.02780 | 0.29556 | −0.33863 | 0.00152 | 0.00023 | 0.00009 | 0.00073 | 0.00256 | 0.00969 | 0.00593 | 0.00002 | 0.00028 |
| Br | −0.00473 | 0.02799 | 0.29715 | −0.34110 | 0.00158 | 0.00027 | 0.00010 | 0.00074 | 0.00257 | 0.00991 | 0.00555 | 0.00003 | 0.00030 |
| Cl | −0.00465 | 0.02802 | 0.29741 | −0.34159 | 0.00159 | 0.00028 | 0.00010 | 0.00075 | 0.00258 | 0.00996 | 0.00557 | 0.00003 | 0.00031 |
| CN | −0.00427 | 0.02817 | 0.29821 | −0.34309 | 0.00152 | 0.00022 | 0.00009 | 0.00075 | 0.00262 | 0.01012 | 0.00564 | 0.00002 | 0.00030 |
| $COCH_3$ | −0.00473 | 0.02818 | 0.29654 | −0.34055 | 0.00145 | 0.00020 | 0.00009 | 0.00075 | 0.00267 | 0.01004 | 0.00539 | 0.00002 | 0.00029 |
| $COOC_2H_5$ | −0.00482 | 0.02811 | 0.29640 | −0.34024 | 0.00147 | 0.00021 | 0.00009 | 0.00075 | 0.00266 | 0.00998 | 0.00546 | 0.00001 | 0.00029 |
| COOH | −0.00465 | 0.02815 | 0.29689 | −0.34106 | 0.00148 | 0.00022 | 0.00009 | 0.00075 | 0.00266 | 0.01004 | 0.00547 | 0.00001 | 0.00029 |
| $C(CH_3)_3$ | −0.00566 | 0.02768 | 0.29502 | −0.33798 | 0.00155 | 0.00023 | 0.00010 | 0.00073 | 0.00254 | 0.00958 | 0.00628 | 0.00002 | 0.00029 |
| $CH_3CH_3$ | −0.00559 | 0.02776 | 0.29523 | −0.33788 | 0.00154 | 0.00023 | 0.00010 | 0.00073 | 0.00255 | 0.00961 | 0.00580 | 0.00003 | 0.00029 |
| F | −0.00479 | 0.02797 | 0.29715 | −0.34101 | 0.00160 | 0.00026 | 0.00010 | 0.00074 | 0.00257 | 0.00994 | 0.00546 | 0.00003 | 0.00030 |
| I | −0.00484 | 0.02797 | 0.29683 | −0.34070 | 0.00158 | 0.00027 | 0.00010 | 0.00074 | 0.00256 | 0.00985 | 0.00568 | 0.00003 | 0.00031 |
| $IO_2$ | −0.00402 | 0.02819 | 0.29854 | −0.34368 | 0.00157 | 0.00027 | 0.00009 | 0.00075 | 0.00263 | 0.01016 | 0.00546 | 0.00003 | 0.00031 |
| $CH_3$ | −0.00560 | 0.02777 | 0.29525 | −0.33784 | 0.00154 | 0.00023 | 0.00010 | 0.00073 | 0.00254 | 0.00961 | 0.00577 | 0.00003 | 0.00029 |
| $O(CH_2)_3CH_3$ | −0.00551 | 0.02786 | 0.29528 | −0.33798 | 0.00154 | 0.00023 | 0.00010 | 0.00073 | 0.00257 | 0.00974 | 0.00551 | 0.00002 | 0.00029 |
| $O(CH_3)_4CH_3$ | −0.00551 | 0.02786 | 0.29526 | −0.33796 | 0.00154 | 0.00023 | 0.00010 | 0.00073 | 0.00257 | 0.00974 | 0.00551 | 0.00002 | 0.00029 |
| $O(CH_2)_2CH_3$ | −0.00550 | 0.02786 | 0.29529 | −0.33801 | 0.00154 | 0.00023 | 0.00010 | 0.00073 | 0.00257 | 0.00974 | 0.00551 | 0.00002 | 0.00029 |
| $NH_2$ | −0.00601 | 0.02757 | 0.29458 | −0.33671 | 0.00159 | 0.00024 | 0.00009 | 0.00073 | 0.00249 | 0.00951 | 0.00601 | 0.00003 | 0.00028 |
| $NHCOCH_3$ | −0.00540 | 0.02770 | 0.29604 | −0.33933 | 0.00161 | 0.00024 | 0.00009 | 0.00073 | 0.00253 | 0.00966 | 0.00619 | 0.00002 | 0.00028 |
| $NO_2$ | −0.00399 | 0.02828 | 0.29880 | −0.34430 | 0.00153 | 0.00024 | 0.00009 | 0.00076 | 0.00268 | 0.01035 | 0.00553 | 0.00002 | 0.00030 |
| $OC_6H_5$ | −0.00521 | 0.02791 | 0.29597 | −0.33911 | 0.00155 | 0.00023 | 0.00009 | 0.00074 | 0.00257 | 0.00983 | 0.00548 | 0.00002 | 0.00029 |
| $OCH_2CH_3$ | −0.00562 | 0.02761 | 0.29536 | −0.33841 | 0.00161 | 0.00027 | 0.00010 | 0.00074 | 0.00251 | 0.00961 | 0.00630 | 0.00003 | 0.00029 |
| OH | −0.00535 | 0.02768 | 0.29612 | −0.33931 | 0.00164 | 0.00027 | 0.00010 | 0.00073 | 0.00251 | 0.00967 | 0.00599 | 0.00003 | 0.00029 |
| $OCH(CH_3)_2$ | −0.00567 | 0.02756 | 0.29523 | −0.33830 | 0.00162 | 0.00027 | 0.00010 | 0.00074 | 0.00249 | 0.00959 | 0.00646 | 0.00003 | 0.00029 |
| $OCH_3$ | −0.00558 | 0.02762 | 0.29558 | −0.33866 | 0.00162 | 0.00027 | 0.00010 | 0.00074 | 0.00250 | 0.00963 | 0.00628 | 0.00003 | 0.00029 |
| SH | −0.00509 | 0.02782 | 0.29666 | −0.34027 | 0.00163 | 0.00027 | 0.00010 | 0.00074 | 0.00252 | 0.00976 | 0.00592 | 0.00003 | 0.00030 |
| $Si(CH_3)_3$ | −0.00559 | 0.02773 | 0.29518 | −0.33790 | 0.00154 | 0.00024 | 0.00010 | 0.00073 | 0.00255 | 0.00958 | 0.00595 | 0.00003 | 0.00029 |
| $SCH_3$ | −0.00507 | 0.02792 | 0.29637 | −0.33977 | 0.00155 | 0.00025 | 0.00010 | 0.00074 | 0.00257 | 0.00979 | 0.00561 | 0.00003 | 0.00030 |
| $SOCH_3$ | −0.00471 | 0.02787 | 0.29760 | −0.34174 | 0.00164 | 0.00029 | 0.00010 | 0.00074 | 0.00257 | 0.00986 | 0.00580 | 0.00003 | 0.00030 |
| $SO_2CH_3$ | −0.00401 | 0.02823 | 0.29863 | −0.34383 | 0.00155 | 0.00027 | 0.00009 | 0.00076 | 0.00266 | 0.01022 | 0.00540 | 0.00002 | 0.00031 |
| $SO_2NH_2$ | −0.00402 | 0.02824 | 0.29858 | −0.34383 | 0.00156 | 0.00027 | 0.00009 | 0.00076 | 0.00265 | 0.01023 | 0.00543 | 0.00002 | 0.00031 |
| $CF_3$ | −0.00433 | 0.02814 | 0.29794 | −0.34281 | 0.00153 | 0.00023 | 0.00009 | 0.00075 | 0.00263 | 0.01011 | 0.00572 | 0.00002 | 0.00030 |
| $OCOCH_3$ | −0.00463 | 0.02799 | 0.29759 | −0.34182 | 0.00157 | 0.00024 | 0.00010 | 0.00074 | 0.00258 | 0.01000 | 0.00564 | 0.00003 | 0.00029 |
| $SCOCH_3$ | −0.00462 | 0.02805 | 0.29751 | −0.34174 | 0.00156 | 0.00025 | 0.00010 | 0.00074 | 0.00259 | 0.00996 | 0.00561 | 0.00003 | 0.00030 |

**Table S10.** All LRF-BO values, $\left\{\delta B^{O\text{-}H}/\delta v(L)\right\}_{L}^{All\ atoms}$, of para-substituted benzoic acids at the B3LYP/6-31G level. The numbering of the atoms listed in the top row is the same as presented in Figure 9 in the text.

| | **O(1)** | **C(2)** | **O(3)** | **H(4)** | **C(5)** | **C(6)** | **C(7)** | **C(8)** | **C(9)** | **C(10)** | **H(11)** | **H(12)** | **H(14)** | **H(15)** |
|---|---|---|---|---|---|---|---|---|---|---|---|---|---|---|
| H | −0.00541 | 0.02780 | 0.29570 | −0.33833 | 0.00156 | 0.00023 | 0.00010 | 0.00073 | 0.00254 | 0.00961 | 0.00552 | 0.00003 | 0.00029 | −0.00038 |
| $C_6H_5$ | −0.00546 | 0.02784 | 0.29540 | −0.33801 | 0.00158 | 0.00023 | 0.00009 | 0.00072 | 0.00256 | 0.00960 | 0.00553 | 0.00000 | 0.00027 | −0.00037 |
| Br | −0.00489 | 0.02796 | 0.29689 | −0.34047 | 0.00157 | 0.00023 | 0.00011 | 0.00075 | 0.00258 | 0.00980 | 0.00549 | −0.00001 | 0.00029 | −0.00033 |
| Cl | −0.00481 | 0.02799 | 0.29715 | −0.34090 | 0.00157 | 0.00022 | 0.00011 | 0.00075 | 0.00259 | 0.00985 | 0.00549 | −0.00001 | 0.00029 | −0.00032 |
| CN | −0.00442 | 0.02814 | 0.29817 | −0.34270 | 0.00157 | 0.00022 | 0.00010 | 0.00074 | 0.00259 | 0.01009 | 0.00548 | 0.00000 | 0.00029 | −0.00030 |
| $COCH_3$ | −0.00486 | 0.02797 | 0.29704 | −0.34072 | 0.00157 | 0.00023 | 0.00010 | 0.00072 | 0.00257 | 0.00989 | 0.00556 | 0.00000 | 0.00027 | −0.00033 |
| $CO_2C_2H_5$ | −0.00493 | 0.02797 | 0.29678 | −0.34030 | 0.00156 | 0.00023 | 0.00010 | 0.00073 | 0.00257 | 0.00988 | 0.00547 | 0.00001 | 0.00027 | −0.00034 |
| COOH | −0.00475 | 0.02803 | 0.29728 | −0.34112 | 0.00156 | 0.00023 | 0.00010 | 0.00073 | 0.00257 | 0.00996 | 0.00545 | 0.00001 | 0.00027 | −0.00032 |
| $C(CH_3)_3$ | −0.00564 | 0.02778 | 0.29501 | −0.33723 | 0.00157 | 0.00023 | 0.00009 | 0.00071 | 0.00255 | 0.00952 | 0.00550 | 0.00000 | 0.00026 | −0.00038 |
| $CH_2CH_3$ | −0.00560 | 0.02778 | 0.29512 | −0.33746 | 0.00158 | 0.00024 | 0.00009 | 0.00072 | 0.00256 | 0.00953 | 0.00552 | 0.00000 | 0.00028 | −0.00039 |
| F | −0.00500 | 0.02795 | 0.29668 | −0.34018 | 0.00159 | 0.00023 | 0.00010 | 0.00074 | 0.00258 | 0.00978 | 0.00553 | 0.00000 | 0.00028 | −0.00033 |
| I | −0.00498 | 0.02795 | 0.29672 | −0.34013 | 0.00156 | 0.00023 | 0.00010 | 0.00075 | 0.00258 | 0.00978 | 0.00546 | −0.00001 | 0.00029 | −0.00033 |
| $IO_2$ | −0.00430 | 0.02815 | 0.29866 | −0.34332 | 0.00154 | 0.00022 | 0.00012 | 0.00076 | 0.00259 | 0.01013 | 0.00543 | 0.00001 | 0.00030 | −0.00029 |
| $CH(CH_3)_2$ | −0.00561 | 0.02778 | 0.29513 | −0.33742 | 0.00157 | 0.00023 | 0.00009 | 0.00072 | 0.00255 | 0.00953 | 0.00553 | 0.00000 | 0.00028 | −0.00038 |
| $CH_3$ | −0.00559 | 0.02778 | 0.29514 | −0.33748 | 0.00157 | 0.00024 | 0.00009 | 0.00072 | 0.00256 | 0.00953 | 0.00551 | −0.00001 | 0.00028 | −0.00038 |
| SCN | −0.00468 | 0.02808 | 0.29739 | −0.34150 | 0.00160 | 0.00023 | 0.00010 | 0.00073 | 0.00260 | 0.00993 | 0.00554 | −0.00001 | 0.00029 | −0.00031 |
| $N(CH_3)_2$ | −0.00648 | 0.02764 | 0.29286 | −0.33396 | 0.00163 | 0.00024 | 0.00008 | 0.00069 | 0.00258 | 0.00918 | 0.00569 | −0.00001 | 0.00025 | −0.00044 |
| $NH_2$ | −0.00632 | 0.02766 | 0.29329 | −0.33461 | 0.00164 | 0.00024 | 0.00009 | 0.00070 | 0.00258 | 0.00922 | 0.00566 | −0.00001 | 0.00028 | −0.00043 |
| $NHCOCH_3$ | −0.00553 | 0.02792 | 0.29500 | −0.33763 | 0.00157 | 0.00022 | 0.00009 | 0.00072 | 0.00260 | 0.00958 | 0.00552 | 0.00000 | 0.00028 | −0.00037 |
| $NHCH_3$ | −0.00643 | 0.02762 | 0.29306 | −0.33418 | 0.00164 | 0.00024 | 0.00009 | 0.00070 | 0.00257 | 0.00918 | 0.00566 | −0.00001 | 0.00026 | −0.00044 |
| $NO_2$ | −0.00413 | 0.02823 | 0.29899 | −0.34406 | 0.00157 | 0.00022 | 0.00011 | 0.00074 | 0.00260 | 0.01027 | 0.00545 | 0.00001 | 0.00026 | −0.00027 |
| $OC_6H_5$ | −0.00556 | 0.02783 | 0.29515 | −0.33762 | 0.00160 | 0.00023 | 0.00009 | 0.00072 | 0.00257 | 0.00954 | 0.00553 | 0.00000 | 0.00027 | −0.00037 |
| $O(CH_2)_3CH_3$ | −0.00579 | 0.02777 | 0.29462 | −0.33669 | 0.00160 | 0.00024 | 0.00009 | 0.00072 | 0.00257 | 0.00945 | 0.00552 | 0.00000 | 0.00026 | −0.00038 |
| $OCOCH_3$ | −0.00527 | 0.02786 | 0.29594 | −0.33886 | 0.00160 | 0.00024 | 0.00010 | 0.00073 | 0.00256 | 0.00968 | 0.00552 | 0.00000 | 0.00025 | −0.00035 |
| $OCH_2CH_3$ | −0.00577 | 0.02777 | 0.29465 | −0.33676 | 0.00160 | 0.00024 | 0.00009 | 0.00072 | 0.00257 | 0.00945 | 0.00553 | 0.00000 | 0.00027 | −0.00038 |
| OH | −0.00556 | 0.02784 | 0.29521 | −0.33771 | 0.00160 | 0.00023 | 0.00010 | 0.00072 | 0.00258 | 0.00953 | 0.00553 | −0.00001 | 0.00028 | −0.00037 |
| $OCH(CH_3)_2$ | −0.00581 | 0.02777 | 0.29449 | −0.33651 | 0.00160 | 0.00024 | 0.00009 | 0.00071 | 0.00257 | 0.00943 | 0.00552 | 0.00000 | 0.00026 | −0.00039 |
| $OCH_3$ | −0.00574 | 0.02778 | 0.29474 | −0.33709 | 0.00162 | 0.00023 | 0.00009 | 0.00071 | 0.00258 | 0.00947 | 0.00569 | 0.00000 | 0.00027 | −0.00039 |
| $O(CH_3)_4CH_3$ | −0.00580 | 0.02777 | 0.29460 | −0.33666 | 0.00160 | 0.00024 | 0.00009 | 0.00072 | 0.00256 | 0.00945 | 0.00552 | 0.00000 | 0.00026 | −0.00038 |
| $O(CH_2)_2CH_3$ | −0.00577 | 0.02777 | 0.29460 | −0.33669 | 0.00160 | 0.00024 | 0.00009 | 0.00072 | 0.00257 | 0.00944 | 0.00552 | 0.00000 | 0.00027 | −0.00038 |
| $SCH(CH_3)_2$ | −0.00529 | 0.02784 | 0.29588 | −0.33875 | 0.00157 | 0.00023 | 0.00010 | 0.00074 | 0.00257 | 0.00964 | 0.00554 | 0.00000 | 0.00029 | −0.00036 |
| $SC_2H_5$ | −0.00548 | 0.02784 | 0.29530 | −0.33786 | 0.00157 | 0.00023 | 0.00010 | 0.00073 | 0.00257 | 0.00955 | 0.00549 | 0.00000 | 0.00028 | −0.00037 |
| SH | −0.00519 | 0.02791 | 0.29607 | −0.33915 | 0.00157 | 0.00023 | 0.00010 | 0.00074 | 0.00259 | 0.00966 | 0.00550 | −0.00001 | 0.00029 | −0.00035 |

**Table S10.** *Cont.*

| | O(1) | C(2) | O(3) | H(4) | C(5) | C(6) | C(7) | C(8) | C(9) | C(10) | H(11) | H(12) | H(14) | H(15) |
|---|---|---|---|---|---|---|---|---|---|---|---|---|---|---|
| $Si(CH_2CH_3)_3$ | −0.00551 | 0.02780 | 0.29537 | −0.33784 | 0.00155 | 0.00023 | 0.00009 | 0.00073 | 0.00255 | 0.00959 | 0.00551 | 0.00000 | 0.00029 | −0.00038 |
| $Si(CH_3)_3$ | −0.00552 | 0.02779 | 0.29537 | −0.33781 | 0.00155 | 0.00023 | 0.00010 | 0.00073 | 0.00255 | 0.00958 | 0.00550 | 0.00000 | 0.00029 | −0.00038 |
| $SCH_3$ | −0.00542 | 0.02785 | 0.29545 | −0.33811 | 0.00157 | 0.00023 | 0.00010 | 0.00073 | 0.00257 | 0.00958 | 0.00549 | 0.00000 | 0.00028 | −0.00037 |
| $SO_2CH_3$ | −0.00426 | 0.02814 | 0.29875 | −0.34353 | 0.00156 | 0.00022 | 0.00012 | 0.00076 | 0.00259 | 0.01016 | 0.00547 | 0.00001 | 0.00029 | −0.00028 |
| $SO_2NH_2$ | −0.00426 | 0.02814 | 0.29872 | −0.34352 | 0.00156 | 0.00022 | 0.00012 | 0.00076 | 0.00260 | 0.01017 | 0.00547 | 0.00001 | 0.00029 | −0.00028 |
| $SOCH_3$ | −0.00482 | 0.02794 | 0.29738 | −0.34105 | 0.00157 | 0.00022 | 0.00011 | 0.00074 | 0.00256 | 0.00987 | 0.00557 | 0.00001 | 0.00028 | −0.00033 |
| $CF_3$ | −0.00445 | 0.02810 | 0.29811 | −0.34254 | 0.00157 | 0.00023 | 0.00011 | 0.00074 | 0.00259 | 0.01007 | 0.00547 | 0.00000 | 0.00028 | −0.00030 |
| $SCOCH_3$ | −0.00514 | 0.02788 | 0.29629 | −0.33940 | 0.00157 | 0.00023 | 0.00010 | 0.00074 | 0.00257 | 0.00972 | 0.00550 | −0.00001 | 0.00029 | −0.00035 |

**Table S11.** All LRF-BO values, $\left\{\delta B^{O\text{-}H}/\delta v(L)\right\}_{L}^{All\ atoms}$, of meta-substituted benzoic acids at the B3LYP/6-31G** level. The numbering of the atoms listed in the top row is the same as presented in Figure 9 in the text.

| | O(1) | C(2) | O(3) | H(4) | C(5) | C(6) | C(7) | C(8) | C(9) | C(10) | H(11) | H(13) | H(15) |
|---|---|---|---|---|---|---|---|---|---|---|---|---|---|
| H | −0.00650 | 0.03376 | 0.28371 | −0.32881 | 0.00165 | 0.00026 | 0.00042 | 0.00069 | 0.00235 | 0.00719 | 0.00551 | 0.00010 | 0.00030 |
| $C_6H_5$ | −0.00646 | 0.03377 | 0.28338 | −0.32907 | 0.00165 | 0.00027 | 0.00041 | 0.00069 | 0.00236 | 0.00726 | 0.00597 | 0.00009 | 0.00029 |
| Br | −0.00550 | 0.03405 | 0.28387 | −0.33077 | 0.00167 | 0.00029 | 0.00041 | 0.00070 | 0.00238 | 0.00749 | 0.00562 | 0.00009 | 0.00031 |
| Cl | −0.00549 | 0.03405 | 0.28395 | −0.33081 | 0.00167 | 0.00029 | 0.00041 | 0.00070 | 0.00238 | 0.00749 | 0.00558 | 0.00009 | 0.00031 |
| CN | −0.00465 | 0.03430 | 0.28422 | −0.33250 | 0.00164 | 0.00026 | 0.00040 | 0.00071 | 0.00244 | 0.00770 | 0.00565 | 0.00008 | 0.00030 |
| $COCH_3$ | −0.00543 | 0.03429 | 0.28339 | −0.33041 | 0.00156 | 0.00025 | 0.00040 | 0.00071 | 0.00246 | 0.00758 | 0.00541 | 0.00008 | 0.00029 |
| $COOC_2H_5$ | −0.00560 | 0.03419 | 0.28340 | −0.33018 | 0.00158 | 0.00026 | 0.00041 | 0.00071 | 0.00246 | 0.00754 | 0.00546 | 0.00007 | 0.00029 |
| COOH | −0.00537 | 0.03424 | 0.28356 | −0.33070 | 0.00159 | 0.00026 | 0.00041 | 0.00071 | 0.00247 | 0.00760 | 0.00546 | 0.00007 | 0.00029 |
| $C(CH_3)_3$ | −0.00687 | 0.03361 | 0.28308 | −0.32853 | 0.00169 | 0.00027 | 0.00041 | 0.00069 | 0.00232 | 0.00716 | 0.00642 | 0.00008 | 0.00029 |
| $CH_3CH_3$ | −0.00674 | 0.03369 | 0.28339 | −0.32847 | 0.00166 | 0.00027 | 0.00042 | 0.00069 | 0.00235 | 0.00718 | 0.00584 | 0.00009 | 0.00029 |
| F | −0.00576 | 0.03394 | 0.28396 | −0.33026 | 0.00171 | 0.00030 | 0.00042 | 0.00069 | 0.00236 | 0.00746 | 0.00540 | 0.00009 | 0.00030 |
| I | −0.00557 | 0.03402 | 0.28377 | −0.33055 | 0.00167 | 0.00028 | 0.00041 | 0.00070 | 0.00237 | 0.00743 | 0.00572 | 0.00009 | 0.00031 |
| $IO_2$ | −0.00446 | 0.03433 | 0.28411 | −0.33246 | 0.00164 | 0.00027 | 0.00040 | 0.00071 | 0.00246 | 0.00772 | 0.00545 | 0.00009 | 0.00031 |
| $CH_3$ | −0.00676 | 0.03370 | 0.28339 | −0.32841 | 0.00165 | 0.00027 | 0.00042 | 0.00069 | 0.00235 | 0.00718 | 0.00581 | 0.00009 | 0.00029 |
| $O(CH_2)_3CH_3$ | −0.00663 | 0.03381 | 0.28324 | −0.32836 | 0.00173 | 0.00028 | 0.00038 | 0.00068 | 0.00231 | 0.00727 | 0.00556 | 0.00007 | 0.00028 |
| $O(CH_3)_4CH_3$ | −0.00663 | 0.03381 | 0.28324 | −0.32836 | 0.00172 | 0.00028 | 0.00038 | 0.00068 | 0.00231 | 0.00727 | 0.00555 | 0.00007 | 0.00028 |
| $O(CH_2)_2CH_3$ | −0.00662 | 0.03381 | 0.28325 | −0.32839 | 0.00173 | 0.00028 | 0.00038 | 0.00068 | 0.00231 | 0.00727 | 0.00556 | 0.00008 | 0.00028 |
| $NH_2$ | −0.00710 | 0.03353 | 0.28323 | −0.32784 | 0.00174 | 0.00029 | 0.00039 | 0.00068 | 0.00228 | 0.00710 | 0.00598 | 0.00009 | 0.00029 |
| $NHCOCH_3$ | −0.00658 | 0.03360 | 0.28377 | −0.32944 | 0.00175 | 0.00029 | 0.00041 | 0.00068 | 0.00232 | 0.00724 | 0.00621 | 0.00008 | 0.00028 |
| $NO_2$ | −0.00440 | 0.03441 | 0.28414 | −0.33291 | 0.00165 | 0.00028 | 0.00041 | 0.00072 | 0.00249 | 0.00789 | 0.00552 | 0.00007 | 0.00030 |
| $OC_6H_5$ | −0.00619 | 0.03389 | 0.28354 | −0.32927 | 0.00169 | 0.00028 | 0.00040 | 0.00069 | 0.00235 | 0.00736 | 0.00550 | 0.00008 | 0.00029 |
| $OCH_2CH_3$ | −0.00661 | 0.03383 | 0.28328 | −0.32844 | 0.00172 | 0.00028 | 0.00038 | 0.00068 | 0.00231 | 0.00728 | 0.00555 | 0.00008 | 0.00029 |
| OH | −0.00658 | 0.03357 | 0.28370 | −0.32909 | 0.00176 | 0.00031 | 0.00042 | 0.00068 | 0.00231 | 0.00722 | 0.00597 | 0.00009 | 0.00029 |
| $OCH(CH_3)_2$ | −0.00699 | 0.03345 | 0.28306 | −0.32841 | 0.00172 | 0.00030 | 0.00042 | 0.00069 | 0.00230 | 0.00714 | 0.00660 | 0.00009 | 0.00029 |
| $OCH_3$ | −0.00683 | 0.03353 | 0.28322 | −0.32864 | 0.00172 | 0.00030 | 0.00042 | 0.00069 | 0.00232 | 0.00718 | 0.00639 | 0.00009 | 0.00029 |
| SH | −0.00598 | 0.03394 | 0.28355 | −0.32980 | 0.00167 | 0.00027 | 0.00040 | 0.00069 | 0.00234 | 0.00734 | 0.00580 | 0.00009 | 0.00030 |
| $Si(CH_3)_3$ | −0.00680 | 0.03369 | 0.28324 | −0.32842 | 0.00164 | 0.00026 | 0.00042 | 0.00070 | 0.00236 | 0.00715 | 0.00607 | 0.00009 | 0.00029 |
| $SCH_3$ | −0.00658 | 0.03363 | 0.28349 | −0.32928 | 0.00170 | 0.00029 | 0.00042 | 0.00069 | 0.00232 | 0.00720 | 0.00640 | 0.00009 | 0.00030 |
| $SOCH_3$ | −0.00568 | 0.03387 | 0.28421 | −0.33092 | 0.00172 | 0.00029 | 0.00041 | 0.00070 | 0.00237 | 0.00743 | 0.00583 | 0.00009 | 0.00030 |
| $SO_2CH_3$ | −0.00478 | 0.03431 | 0.28400 | −0.33203 | 0.00161 | 0.00027 | 0.00041 | 0.00071 | 0.00246 | 0.00769 | 0.00553 | 0.00008 | 0.00030 |
| $SO_2NH_2$ | −0.00499 | 0.03426 | 0.28387 | −0.33162 | 0.00161 | 0.00028 | 0.00041 | 0.00071 | 0.00245 | 0.00765 | 0.00556 | 0.00008 | 0.00030 |
| $CF_3$ | −0.00522 | 0.03414 | 0.28386 | −0.33134 | 0.00164 | 0.00027 | 0.00041 | 0.00070 | 0.00243 | 0.00758 | 0.00575 | 0.00008 | 0.00029 |
| $OCOCH_3$ | −0.00536 | 0.03401 | 0.28412 | −0.33115 | 0.00168 | 0.00028 | 0.00041 | 0.00069 | 0.00238 | 0.00754 | 0.00558 | 0.00009 | 0.00030 |
| $SCOCH_3$ | −0.00532 | 0.03413 | 0.28409 | −0.33125 | 0.00162 | 0.00027 | 0.00041 | 0.00070 | 0.00239 | 0.00753 | 0.00561 | 0.00009 | 0.00031 |

**Table S12.** All LRF-BO values, $\left\{\delta B^{\text{O-H}}/\delta v(\text{L})\right\}_{\text{L}}^{\textit{All atoms}}$, of para-substituted benzoic acids at the B3LYP/6-31G** level. The numbering of the atoms listed in the top row is the same as presented in Figure 9 in the text.

| | O(1) | C(2) | O(3) | H(4) | C(5) | C(6) | C(7) | C(8) | C(9) | C(10) | H(11) | H(12) | H(14) | H(15) |
|---|---|---|---|---|---|---|---|---|---|---|---|---|---|---|
| H | −0.00650 | 0.03376 | 0.28371 | −0.32881 | 0.00165 | 0.00026 | 0.00042 | 0.00069 | 0.00235 | 0.00719 | 0.00551 | 0.00010 | 0.00030 | −0.00061 |
| $C_6H_5$ | −0.00661 | 0.03375 | 0.28369 | −0.32873 | 0.00165 | 0.00026 | 0.00043 | 0.00068 | 0.00238 | 0.00720 | 0.00547 | −0.00004 | 0.00027 | −0.00060 |
| Br | −0.00585 | 0.03395 | 0.28417 | −0.33039 | 0.00165 | 0.00025 | 0.00043 | 0.00071 | 0.00240 | 0.00740 | 0.00545 | −0.00005 | 0.00029 | −0.00056 |
| Cl | −0.00585 | 0.03394 | 0.28420 | −0.33043 | 0.00165 | 0.00025 | 0.00043 | 0.00071 | 0.00240 | 0.00740 | 0.00547 | −0.00005 | 0.00029 | −0.00056 |
| CN | −0.00485 | 0.03426 | 0.28455 | −0.33234 | 0.00164 | 0.00026 | 0.00040 | 0.00067 | 0.00240 | 0.00763 | 0.00548 | −0.00004 | 0.00029 | −0.00053 |
| $COCH_3$ | −0.00566 | 0.03400 | 0.28405 | −0.33049 | 0.00166 | 0.00027 | 0.00041 | 0.00065 | 0.00236 | 0.00742 | 0.00553 | −0.00003 | 0.00026 | −0.00057 |
| $CO_2C_2H_5$ | −0.00572 | 0.03400 | 0.28386 | −0.33019 | 0.00166 | 0.00028 | 0.00042 | 0.00066 | 0.00238 | 0.00740 | 0.00548 | −0.00003 | 0.00026 | −0.00057 |
| COOH | −0.00548 | 0.03407 | 0.28402 | −0.33071 | 0.00165 | 0.00028 | 0.00042 | 0.00066 | 0.00238 | 0.00747 | 0.00546 | −0.00003 | 0.00026 | −0.00056 |
| $C(CH_3)_3$ | −0.00688 | 0.03371 | 0.28352 | −0.32812 | 0.00167 | 0.00026 | 0.00044 | 0.00068 | 0.00237 | 0.00716 | 0.00546 | −0.00003 | 0.00027 | −0.00061 |
| $CH_2CH_3$ | −0.00685 | 0.03368 | 0.28361 | −0.32826 | 0.00166 | 0.00026 | 0.00045 | 0.00070 | 0.00237 | 0.00716 | 0.00548 | −0.00004 | 0.00028 | −0.00061 |
| F | −0.00629 | 0.03383 | 0.28404 | −0.32970 | 0.00167 | 0.00025 | 0.00042 | 0.00072 | 0.00240 | 0.00734 | 0.00550 | −0.00004 | 0.00029 | −0.00057 |
| I | −0.00590 | 0.03394 | 0.28420 | −0.33029 | 0.00163 | 0.00025 | 0.00044 | 0.00071 | 0.00239 | 0.00737 | 0.00543 | −0.00005 | 0.00030 | −0.00056 |
| $IO_2$ | −0.00480 | 0.03424 | 0.28451 | −0.33224 | 0.00163 | 0.00026 | 0.00043 | 0.00070 | 0.00239 | 0.00761 | 0.00549 | −0.00002 | 0.00030 | −0.00052 |
| $CH(CH_3)_2$ | −0.00686 | 0.03369 | 0.28363 | −0.32822 | 0.00165 | 0.00026 | 0.00044 | 0.00069 | 0.00238 | 0.00715 | 0.00547 | −0.00003 | 0.00028 | −0.00061 |
| $CH_3$ | −0.00684 | 0.03369 | 0.28364 | −0.32826 | 0.00166 | 0.00026 | 0.00045 | 0.00070 | 0.00237 | 0.00715 | 0.00546 | −0.00004 | 0.00029 | −0.00061 |
| SCN | −0.00568 | 0.03399 | 0.28422 | −0.33081 | 0.00166 | 0.00025 | 0.00040 | 0.00068 | 0.00240 | 0.00743 | 0.00548 | −0.00004 | 0.00029 | −0.00055 |
| $N(CH_3)_2$ | −0.00841 | 0.03324 | 0.28306 | −0.32582 | 0.00167 | 0.00026 | 0.00038 | 0.00075 | 0.00240 | 0.00694 | 0.00554 | −0.00004 | 0.00027 | −0.00066 |
| $NH_2$ | −0.00799 | 0.03335 | 0.28337 | −0.32666 | 0.00167 | 0.00026 | 0.00043 | 0.00075 | 0.00240 | 0.00700 | 0.00553 | −0.00004 | 0.00029 | −0.00065 |
| $NHCOCH_3$ | −0.00686 | 0.03377 | 0.28342 | −0.32831 | 0.00164 | 0.00024 | 0.00041 | 0.00071 | 0.00241 | 0.00720 | 0.00546 | −0.00003 | 0.00029 | −0.00060 |
| $NHCH_3$ | −0.00828 | 0.03324 | 0.28324 | −0.32611 | 0.00165 | 0.00026 | 0.00041 | 0.00076 | 0.00241 | 0.00695 | 0.00552 | −0.00004 | 0.00028 | −0.00065 |
| $NO_2$ | −0.00459 | 0.03435 | 0.28461 | −0.33278 | 0.00165 | 0.00027 | 0.00039 | 0.00066 | 0.00240 | 0.00774 | 0.00544 | −0.00002 | 0.00025 | −0.00051 |
| $OC_6H_5$ | −0.00702 | 0.03362 | 0.28363 | −0.32818 | 0.00166 | 0.00025 | 0.00042 | 0.00072 | 0.00241 | 0.00717 | 0.00546 | −0.00004 | 0.00028 | −0.00060 |
| $O(CH_2)_3CH_3$ | −0.00746 | 0.03352 | 0.28344 | −0.32757 | 0.00171 | 0.00026 | 0.00042 | 0.00072 | 0.00238 | 0.00711 | 0.00562 | −0.00003 | 0.00028 | −0.00063 |
| $OCOCH_3$ | −0.00647 | 0.03375 | 0.28388 | −0.32918 | 0.00167 | 0.00026 | 0.00043 | 0.00070 | 0.00239 | 0.00728 | 0.00548 | −0.00004 | 0.00027 | −0.00058 |
| $OCH_2CH_3$ | −0.00740 | 0.03353 | 0.28343 | −0.32764 | 0.00171 | 0.00026 | 0.00042 | 0.00072 | 0.00238 | 0.00712 | 0.00562 | −0.00003 | 0.00028 | −0.00063 |
| OH | −0.00712 | 0.03361 | 0.28365 | −0.32812 | 0.00165 | 0.00025 | 0.00042 | 0.00075 | 0.00242 | 0.00716 | 0.00547 | −0.00004 | 0.00029 | −0.00060 |
| $OCH(CH_3)_2$ | −0.00744 | 0.03350 | 0.28344 | −0.32739 | 0.00164 | 0.00025 | 0.00042 | 0.00074 | 0.00243 | 0.00709 | 0.00544 | −0.00004 | 0.00028 | −0.00061 |
| $OCH_3$ | −0.00726 | 0.03355 | 0.28352 | −0.32771 | 0.00163 | 0.00025 | 0.00041 | 0.00074 | 0.00243 | 0.00712 | 0.00544 | −0.00004 | 0.00028 | −0.00060 |
| $O(CH_3)_4CH_3$ | −0.00746 | 0.03352 | 0.28344 | −0.32754 | 0.00171 | 0.00026 | 0.00042 | 0.00072 | 0.00238 | 0.00710 | 0.00561 | −0.00003 | 0.00028 | −0.00063 |
| $O(CH_2)_2CH_3$ | −0.00744 | 0.03352 | 0.28344 | −0.32758 | 0.00171 | 0.00026 | 0.00042 | 0.00072 | 0.00238 | 0.00711 | 0.00562 | −0.00003 | 0.00028 | −0.00063 |
| $SCH(CH_3)_2$ | −0.00689 | 0.03364 | 0.28369 | −0.32849 | 0.00166 | 0.00025 | 0.00043 | 0.00071 | 0.00238 | 0.00715 | 0.00554 | −0.00003 | 0.00029 | −0.00061 |
| $SC_2H_5$ | −0.00696 | 0.03362 | 0.28364 | −0.32838 | 0.00167 | 0.00025 | 0.00043 | 0.00071 | 0.00237 | 0.00715 | 0.00556 | −0.00003 | 0.00029 | −0.00061 |
| SH | −0.00648 | 0.03377 | 0.28385 | −0.32919 | 0.00164 | 0.00025 | 0.00044 | 0.00072 | 0.00240 | 0.00724 | 0.00546 | −0.00005 | 0.00029 | −0.00059 |

**Table S12.** *Cont.*

| | O(1) | C(2) | O(3) | H(4) | C(5) | C(6) | C(7) | C(8) | C(9) | C(10) | H(11) | H(12) | H(14) | H(15) |
|---|---|---|---|---|---|---|---|---|---|---|---|---|---|---|
| $Si(CH_2CH_3)_3$ | −0.00659 | 0.03376 | 0.28358 | −0.32855 | 0.00164 | 0.00026 | 0.00045 | 0.00068 | 0.00236 | 0.00718 | 0.00548 | −0.00003 | 0.00029 | −0.00060 |
| $Si(CH_3)_3$ | −0.00665 | 0.03375 | 0.28359 | −0.32846 | 0.00165 | 0.00026 | 0.00046 | 0.00068 | 0.00235 | 0.00716 | 0.00547 | −0.00003 | 0.00030 | −0.00061 |
| $SCH_3$ | −0.00682 | 0.03367 | 0.28364 | −0.32846 | 0.00162 | 0.00024 | 0.00043 | 0.00072 | 0.00241 | 0.00717 | 0.00545 | −0.00004 | 0.00029 | −0.00060 |
| $SO_2CH_3$ | −0.00496 | 0.03420 | 0.28438 | −0.33191 | 0.00164 | 0.00027 | 0.00042 | 0.00069 | 0.00239 | 0.00758 | 0.00549 | −0.00002 | 0.00028 | −0.00053 |
| $SO_2NH_2$ | −0.00518 | 0.03415 | 0.28429 | −0.33150 | 0.00164 | 0.00027 | 0.00043 | 0.00069 | 0.00239 | 0.00754 | 0.00548 | −0.00003 | 0.00028 | −0.00054 |
| $SOCH_3$ | −0.00579 | 0.03393 | 0.28423 | −0.33045 | 0.00164 | 0.00026 | 0.00043 | 0.00069 | 0.00238 | 0.00738 | 0.00555 | −0.00003 | 0.00027 | −0.00057 |
| $CF_3$ | −0.00536 | 0.03411 | 0.28426 | −0.33119 | 0.00165 | 0.00027 | 0.00042 | 0.00068 | 0.00239 | 0.00751 | 0.00548 | −0.00003 | 0.00028 | −0.00055 |
| $SCOCH_3$ | −0.00607 | 0.03391 | 0.28379 | −0.32959 | 0.00163 | 0.00025 | 0.00043 | 0.00069 | 0.00238 | 0.00730 | 0.00545 | −0.00004 | 0.00029 | −0.00058 |

**Table S13.** All LRF-BO values, $\left\{\delta B^{O\text{-}H}/\delta v(L)\right\}_L^{All\ atoms}$, of meta-substituted benzoic acids at the B3LYP/6-31++G** level. The numbering of the atoms listed in the top row is the same as presented in Figure 9 in the text.

| | O(1) | C(2) | O(3) | H(4) | C(5) | C(6) | C(7) | C(8) | C(9) | C(10) | H(11) | H(13) | H(15) |
|---|---|---|---|---|---|---|---|---|---|---|---|---|---|
| H | 0.00431 | 0.03198 | 0.36323 | −0.39894 | −0.00021 | 0.00018 | 0.00040 | 0.00094 | 0.00221 | 0.00400 | −0.00828 | −0.00012 | 0.00037 |
| $C_6H_5$ | 0.00629 | 0.03143 | 0.37215 | −0.41081 | 0.00016 | 0.00039 | 0.00040 | 0.00094 | 0.00243 | 0.00488 | −0.00870 | 0.00005 | 0.00042 |
| Br | 0.00513 | 0.03228 | 0.36400 | −0.40109 | −0.00003 | 0.00041 | 0.00047 | 0.00094 | 0.00220 | 0.00438 | −0.00867 | −0.00008 | 0.00039 |
| Cl | 0.00546 | 0.03210 | 0.36445 | −0.40233 | 0.00001 | 0.00049 | 0.00048 | 0.00094 | 0.00218 | 0.00460 | −0.00838 | 0.00006 | 0.00038 |
| CN | 0.00701 | 0.03204 | 0.36954 | −0.40916 | −0.00023 | 0.00022 | 0.00034 | 0.00090 | 0.00241 | 0.00496 | −0.00874 | 0.00011 | 0.00042 |
| $COCH_3$ | 0.00662 | 0.03241 | 0.37292 | −0.40973 | −0.00021 | 0.00024 | 0.00033 | 0.00099 | 0.00259 | 0.00565 | −0.01197 | −0.00001 | 0.00059 |
| $COOC_2H_5$ | 0.00626 | 0.03251 | 0.37314 | −0.40931 | −0.00026 | 0.00025 | 0.00037 | 0.00097 | 0.00253 | 0.00519 | −0.01153 | −0.00009 | 0.00048 |
| COOH | 0.00687 | 0.03230 | 0.37140 | −0.41078 | −0.00043 | 0.00016 | 0.00036 | 0.00094 | 0.00249 | 0.00535 | −0.00852 | 0.00004 | 0.00043 |
| $C(CH_3)_3$ | 0.00405 | 0.03247 | 0.37229 | −0.40648 | 0.00070 | 0.00043 | 0.00040 | 0.00094 | 0.00238 | 0.00415 | −0.01141 | −0.00022 | 0.00039 |
| $CH_3CH_3$ | 0.00526 | 0.03232 | 0.36887 | −0.40544 | −0.00011 | 0.00030 | 0.00040 | 0.00089 | 0.00229 | 0.00402 | −0.00886 | −0.00002 | 0.00043 |
| F | 0.00500 | 0.03198 | 0.36257 | −0.40046 | 0.00008 | 0.00029 | 0.00050 | 0.00097 | 0.00220 | 0.00486 | −0.00823 | −0.00003 | 0.00040 |
| I | 0.00524 | 0.03187 | 0.36407 | −0.40164 | 0.00002 | 0.00046 | 0.00044 | 0.00095 | 0.00224 | 0.00450 | −0.00863 | 0.00006 | 0.00043 |
| $IO_2$ | 0.00689 | 0.03263 | 0.36959 | −0.40826 | −0.00037 | 0.00025 | 0.00037 | 0.00097 | 0.00242 | 0.00504 | −0.00936 | −0.00001 | 0.00045 |
| $CH_3$ | 0.00518 | 0.03226 | 0.36807 | −0.40527 | −0.00020 | 0.00027 | 0.00040 | 0.00090 | 0.00232 | 0.00422 | −0.00834 | 0.00005 | 0.00044 |
| $O(CH_2)_3CH_3$ | 0.00404 | 0.03263 | 0.36323 | −0.40068 | −0.00020 | 0.00019 | 0.00037 | 0.00088 | 0.00218 | 0.00510 | −0.00770 | −0.00005 | 0.00035 |
| $O(CH_3)_4CH_3$ | 0.00428 | 0.03269 | 0.36486 | −0.40160 | −0.00022 | 0.00022 | 0.00040 | 0.00095 | 0.00216 | 0.00506 | −0.00847 | −0.00005 | 0.00045 |
| $O(CH_2)_2CH_3$ | 0.00400 | 0.03272 | 0.36254 | −0.39962 | −0.00019 | 0.00017 | 0.00034 | 0.00086 | 0.00216 | 0.00501 | −0.00793 | −0.00006 | 0.00033 |
| $NH_2$ | 0.00409 | 0.03217 | 0.36340 | −0.39959 | 0.00004 | 0.00029 | 0.00043 | 0.00093 | 0.00210 | 0.00431 | −0.00835 | −0.00013 | 0.00035 |
| $NHCOCH_3$ | 0.00490 | 0.03210 | 0.37167 | −0.40987 | −0.00003 | 0.00025 | 0.00044 | 0.00097 | 0.00237 | 0.00496 | −0.00825 | −0.00001 | 0.00034 |
| $NO_2$ | 0.00637 | 0.03274 | 0.36879 | −0.40762 | −0.00031 | 0.00013 | 0.00041 | 0.00096 | 0.00242 | 0.00554 | −0.00881 | −0.00008 | 0.00041 |
| $OC_6H_5$ | 0.00502 | 0.03236 | 0.36550 | −0.40278 | −0.00033 | 0.00022 | 0.00042 | 0.00091 | 0.00223 | 0.00486 | −0.00860 | 0.00007 | 0.00029 |
| $OCH_2CH_3$ | 0.00553 | 0.03219 | 0.37411 | −0.41149 | −0.00001 | 0.00021 | 0.00043 | 0.00101 | 0.00242 | 0.00419 | −0.00859 | 0.00001 | 0.00051 |
| OH | 0.00453 | 0.03187 | 0.36429 | −0.40197 | 0.00029 | 0.00027 | 0.00048 | 0.00098 | 0.00219 | 0.00472 | −0.00782 | −0.00017 | 0.00033 |
| $OCH(CH_3)_2$ | 0.00610 | 0.03206 | 0.37823 | −0.41651 | 0.00040 | 0.00023 | 0.00042 | 0.00100 | 0.00248 | 0.00424 | −0.00880 | 0.00000 | 0.00046 |
| $OCH_3$ | 0.00467 | 0.03261 | 0.36397 | −0.40011 | −0.00031 | 0.00012 | 0.00029 | 0.00086 | 0.00207 | 0.00477 | −0.00886 | −0.00007 | 0.00035 |
| SH | 0.00536 | 0.03212 | 0.36695 | −0.40418 | −0.00006 | 0.00031 | 0.00036 | 0.00091 | 0.00218 | 0.00431 | −0.00859 | −0.00010 | 0.00042 |
| $Si(CH_3)_3$ | 0.00568 | 0.03160 | 0.37935 | −0.41585 | 0.00000 | 0.00051 | 0.00044 | 0.00105 | 0.00263 | 0.00388 | −0.01033 | 0.00003 | 0.00054 |
| $SCH_3$ | 0.00615 | 0.03138 | 0.37615 | −0.41432 | 0.00041 | 0.00035 | 0.00037 | 0.00101 | 0.00245 | 0.00443 | −0.00931 | 0.00010 | 0.00050 |
| $SOCH_3$ | 0.00606 | 0.03216 | 0.37048 | −0.40827 | 0.00030 | 0.00043 | 0.00039 | 0.00092 | 0.00222 | 0.00432 | −0.00926 | −0.00006 | 0.00043 |
| $SO_2CH_3$ | 0.00726 | 0.03261 | 0.37320 | −0.41237 | −0.00025 | 0.00035 | 0.00036 | 0.00091 | 0.00241 | 0.00491 | −0.00928 | 0.00004 | 0.00050 |
| $SO_2NH_2$ | 0.00698 | 0.03242 | 0.37212 | −0.41133 | 0.00000 | 0.00040 | 0.00039 | 0.00093 | 0.00238 | 0.00487 | −0.00880 | 0.00001 | 0.00049 |
| $CF_3$ | 0.00680 | 0.03222 | 0.37071 | −0.41006 | 0.00005 | 0.00029 | 0.00036 | 0.00092 | 0.00233 | 0.00524 | −0.00858 | 0.00004 | 0.00044 |
| $OCOCH_3$ | 0.00601 | 0.03226 | 0.36869 | −0.40635 | −0.00034 | 0.00021 | 0.00039 | 0.00095 | 0.00228 | 0.00494 | −0.00886 | −0.00006 | 0.00039 |
| $SCOCH_3$ | 0.00621 | 0.03225 | 0.37047 | −0.40783 | −0.00014 | 0.00043 | 0.00042 | 0.00092 | 0.00228 | 0.00436 | −0.00964 | −0.00006 | 0.00044 |

**Table S14.** All LRF-BO values, $\left\{\delta B^{O\text{-}H}/\delta v(L)\right\}_{L}^{All\ atoms}$, of para-substituted benzoic acids at the B3LYP/6-31++G** level. The numbering of the atoms listed in the top row is the same as presented in Figure 9 in the text.

| | O(1) | C(2) | O(3) | H(4) | C(5) | C(6) | C(7) | C(8) | C(9) | C(10) | H(11) | H(12) | H(14) | H(15) |
|---|---|---|---|---|---|---|---|---|---|---|---|---|---|---|
| H | 0.00431 | 0.03198 | 0.36323 | −0.39894 | −0.00021 | 0.00018 | 0.00040 | 0.00094 | 0.00221 | 0.00400 | −0.00828 | −0.00012 | 0.00037 | −0.00063 |
| $C_6H_5$ | 0.00538 | 0.03207 | 0.36478 | −0.39964 | 0.00028 | 0.00025 | 0.00032 | 0.00083 | 0.00223 | 0.00465 | −0.01149 | 0.00055 | 0.00031 | −0.00051 |
| Br | 0.00489 | 0.03200 | 0.36393 | −0.40150 | −0.00033 | 0.00012 | 0.00037 | 0.00089 | 0.00221 | 0.00422 | −0.00724 | 0.00033 | 0.00046 | −0.00062 |
| Cl | 0.00525 | 0.03169 | 0.36552 | −0.40378 | −0.00027 | 0.00020 | 0.00039 | 0.00087 | 0.00227 | 0.00421 | −0.00688 | 0.00049 | 0.00050 | −0.00061 |
| CN | 0.00681 | 0.03201 | 0.36929 | −0.40788 | −0.00032 | 0.00018 | 0.00027 | 0.00080 | 0.00225 | 0.00458 | −0.00824 | 0.00030 | 0.00042 | −0.00062 |
| $COCH_3$ | 0.00580 | 0.03169 | 0.36645 | −0.40128 | 0.00054 | 0.00029 | 0.00034 | 0.00077 | 0.00217 | 0.00493 | −0.01210 | 0.00043 | 0.00029 | −0.00032 |
| $CO_2C_2H_5$ | 0.00647 | 0.03179 | 0.36896 | −0.40480 | 0.00036 | 0.00029 | 0.00035 | 0.00081 | 0.00226 | 0.00497 | −0.01162 | 0.00034 | 0.00025 | −0.00043 |
| COOH | 0.00647 | 0.03213 | 0.36705 | −0.40360 | 0.00036 | 0.00031 | 0.00032 | 0.00084 | 0.00230 | 0.00528 | −0.01156 | 0.00040 | 0.00022 | −0.00057 |
| $C(CH_3)_3$ | 0.00569 | 0.03227 | 0.36891 | −0.40314 | 0.00018 | 0.00030 | 0.00041 | 0.00079 | 0.00214 | 0.00415 | −0.01154 | 0.00032 | 0.00026 | −0.00046 |
| $CH_2CH_3$ | 0.00557 | 0.03156 | 0.36839 | −0.40557 | −0.00018 | 0.00025 | 0.00035 | 0.00090 | 0.00235 | 0.00413 | −0.00764 | 0.00039 | 0.00029 | −0.00074 |
| F | 0.00462 | 0.03186 | 0.36428 | −0.40079 | −0.00021 | 0.00015 | 0.00038 | 0.00089 | 0.00224 | 0.00425 | −0.00812 | 0.00060 | 0.00037 | −0.00061 |
| I | 0.00479 | 0.03187 | 0.36401 | −0.40080 | −0.00032 | 0.00010 | 0.00039 | 0.00092 | 0.00226 | 0.00412 | −0.00790 | 0.00045 | 0.00052 | −0.00061 |
| $IO_2$ | 0.00640 | 0.03203 | 0.36801 | −0.40662 | −0.00020 | 0.00020 | 0.00038 | 0.00090 | 0.00233 | 0.00457 | −0.00818 | 0.00045 | 0.00040 | −0.00062 |
| $CH(CH_3)_2$ | 0.00562 | 0.03184 | 0.36828 | −0.40278 | 0.00032 | 0.00027 | 0.00041 | 0.00089 | 0.00229 | 0.00468 | −0.01199 | 0.00047 | 0.00042 | −0.00055 |
| $CH_3$ | 0.00532 | 0.03171 | 0.36774 | −0.40441 | −0.00031 | 0.00019 | 0.00035 | 0.00088 | 0.00228 | 0.00404 | −0.00783 | 0.00042 | 0.00030 | −0.00072 |
| SCN | 0.00673 | 0.03152 | 0.37115 | −0.41024 | −0.00019 | 0.00028 | 0.00032 | 0.00085 | 0.00234 | 0.00444 | −0.00759 | 0.00029 | 0.00036 | −0.00073 |
| $N(CH_3)_2$ | 0.00392 | 0.03157 | 0.36493 | −0.39798 | 0.00044 | 0.00030 | 0.00035 | 0.00086 | 0.00212 | 0.00431 | −0.01153 | 0.00037 | 0.00036 | −0.00030 |
| $NH_2$ | 0.00349 | 0.03178 | 0.36419 | −0.39941 | −0.00039 | 0.00016 | 0.00033 | 0.00093 | 0.00225 | 0.00371 | −0.00757 | 0.00055 | 0.00032 | −0.00070 |
| $NHCOCH_3$ | 0.00480 | 0.03182 | 0.36682 | −0.40114 | 0.00040 | 0.00025 | 0.00035 | 0.00084 | 0.00225 | 0.00464 | −0.01167 | 0.00028 | 0.00052 | −0.00037 |
| $NHCH_3$ | 0.00466 | 0.03161 | 0.36763 | −0.40496 | −0.00029 | 0.00024 | 0.00033 | 0.00091 | 0.00230 | 0.00370 | −0.00659 | 0.00051 | 0.00028 | −0.00067 |
| $NO_2$ | 0.00675 | 0.03194 | 0.36855 | −0.40797 | −0.00015 | 0.00023 | 0.00031 | 0.00079 | 0.00228 | 0.00488 | −0.00799 | 0.00043 | 0.00029 | −0.00064 |
| $OC_6H_5$ | 0.00588 | 0.03168 | 0.36841 | −0.40632 | −0.00012 | 0.00030 | 0.00037 | 0.00083 | 0.00225 | 0.00413 | −0.00750 | 0.00056 | 0.00040 | −0.00048 |
| $O(CH_2)_3CH_3$ | 0.00393 | 0.03159 | 0.36149 | −0.39603 | 0.00024 | 0.00030 | 0.00036 | 0.00077 | 0.00210 | 0.00419 | −0.00998 | 0.00062 | 0.00035 | −0.00014 |
| $OCOCH_3$ | 0.00607 | 0.03155 | 0.36884 | −0.40672 | −0.00023 | 0.00025 | 0.00034 | 0.00084 | 0.00223 | 0.00422 | −0.00766 | 0.00039 | 0.00041 | −0.00064 |
| $OCH_2CH_3$ | 0.00425 | 0.03222 | 0.36420 | −0.39961 | −0.00037 | 0.00029 | 0.00030 | 0.00080 | 0.00213 | 0.00351 | −0.00814 | 0.00032 | 0.00026 | −0.00050 |
| OH | 0.00415 | 0.03199 | 0.36443 | −0.40016 | −0.00036 | 0.00017 | 0.00035 | 0.00091 | 0.00224 | 0.00392 | −0.00803 | 0.00042 | 0.00027 | −0.00071 |
| $OCH(CH_3)_2$ | 0.00505 | 0.03189 | 0.36713 | −0.40432 | −0.00040 | 0.00027 | 0.00033 | 0.00083 | 0.00218 | 0.00360 | −0.00711 | 0.00041 | 0.00039 | −0.00043 |
| $OCH_3$ | 0.00583 | 0.03147 | 0.36911 | −0.40798 | −0.00014 | 0.00029 | 0.00035 | 0.00088 | 0.00234 | 0.00415 | −0.00677 | 0.00054 | 0.00022 | −0.00066 |
| $O(CH_3)_4CH_3$ | 0.00387 | 0.03174 | 0.36145 | −0.39605 | 0.00016 | 0.00028 | 0.00034 | 0.00076 | 0.00207 | 0.00404 | −0.00973 | 0.00060 | 0.00032 | −0.00010 |
| $O(CH_2)_2CH_3$ | 0.00395 | 0.03137 | 0.36123 | −0.39579 | 0.00032 | 0.00031 | 0.00036 | 0.00079 | 0.00213 | 0.00432 | −0.01010 | 0.00066 | 0.00036 | −0.00020 |
| $SCH(CH_3)_2$ | 0.00513 | 0.03147 | 0.36884 | −0.40610 | 0.00004 | 0.00019 | 0.00035 | 0.00081 | 0.00224 | 0.00419 | −0.00760 | 0.00027 | 0.00045 | −0.00050 |
| $SC_2H_5$ | 0.00509 | 0.03146 | 0.36951 | −0.40638 | 0.00009 | 0.00017 | 0.00035 | 0.00084 | 0.00223 | 0.00431 | −0.00816 | 0.00027 | 0.00060 | −0.00050 |
| SH | 0.00493 | 0.03180 | 0.36569 | −0.40271 | −0.00042 | 0.00010 | 0.00032 | 0.00091 | 0.00227 | 0.00404 | −0.00746 | 0.00031 | 0.00032 | −0.00065 |

**Table S14.** *Cont.*

| | O(1) | C(2) | O(3) | H(4) | C(5) | C(6) | C(7) | C(8) | C(9) | C(10) | H(11) | H(12) | H(14) | H(15) |
|---|---|---|---|---|---|---|---|---|---|---|---|---|---|---|
| $Si(CH_2CH_3)_3$ | 0.00537 | 0.03257 | 0.36467 | −0.39902 | 0.00030 | 0.00026 | 0.00035 | 0.00077 | 0.00211 | 0.00426 | −0.01204 | 0.00039 | 0.00051 | −0.00033 |
| $Si(CH_3)_3$ | 0.00612 | 0.03110 | 0.37051 | −0.40729 | 0.00001 | 0.00020 | 0.00035 | 0.00082 | 0.00228 | 0.00431 | −0.00862 | 0.00045 | 0.00058 | −0.00053 |
| $SCH_3$ | 0.00495 | 0.03227 | 0.36510 | −0.40162 | −0.00029 | 0.00021 | 0.00034 | 0.00086 | 0.00212 | 0.00363 | −0.00794 | 0.00007 | 0.00037 | −0.00045 |
| $SO_2CH_3$ | 0.00716 | 0.03164 | 0.37152 | −0.41100 | −0.00002 | 0.00026 | 0.00037 | 0.00086 | 0.00235 | 0.00477 | −0.00780 | 0.00040 | 0.00030 | −0.00062 |
| $SO_2NH_2$ | 0.00672 | 0.03205 | 0.36701 | −0.40411 | 0.00047 | 0.00028 | 0.00039 | 0.00089 | 0.00230 | 0.00533 | −0.01154 | 0.00041 | 0.00021 | −0.00058 |
| $SOCH_3$ | 0.00647 | 0.03171 | 0.37045 | −0.40919 | −0.00018 | 0.00017 | 0.00038 | 0.00086 | 0.00228 | 0.00448 | −0.00755 | 0.00045 | 0.00020 | −0.00070 |
| $CF_3$ | 0.00695 | 0.03165 | 0.37035 | −0.41019 | −0.00011 | 0.00030 | 0.00034 | 0.00087 | 0.00239 | 0.00481 | −0.00737 | 0.00033 | 0.00028 | −0.00066 |
| $SCOCH_3$ | 0.00619 | 0.03145 | 0.36912 | −0.40751 | −0.00010 | 0.00018 | 0.00032 | 0.00085 | 0.00233 | 0.00446 | −0.00756 | 0.00044 | 0.00040 | −0.00063 |

**Table S15.** All LRF-BO values, $\left\{\delta B^{O\text{-}H}/\delta v(L)\right\}_{L}^{All\ atoms}$, of meta-substituted benzoic acids at the B3LYP/6-311G level. The numbering of the atoms listed in the top row is the same as presented in Figure 9 in the text.

| | **O(1)** | **C(2)** | **O(3)** | **H(4)** | **C(5)** | **C(6)** | **C(7)** | **C(8)** | **C(9)** | **C(10)** | **H(11)** | **H(13)** | **H(15)** |
|---|---|---|---|---|---|---|---|---|---|---|---|---|---|
| H | −0.00325 | 0.03320 | 0.34799 | −0.40180 | 0.00081 | 0.00019 | 0.00008 | 0.00071 | 0.00255 | 0.00986 | 0.00904 | 0.00004 | 0.00038 |
| $C_6H_5$ | −0.00292 | 0.03326 | 0.34793 | −0.40237 | 0.00074 | 0.00017 | 0.00008 | 0.00072 | 0.00258 | 0.00985 | 0.00933 | 0.00003 | 0.00038 |
| Br | −0.00275 | 0.03348 | 0.34976 | −0.40462 | 0.00082 | 0.00021 | 0.00008 | 0.00072 | 0.00256 | 0.01009 | 0.00904 | 0.00003 | 0.00039 |
| Cl | −0.00266 | 0.03349 | 0.35023 | −0.40517 | 0.00084 | 0.00022 | 0.00009 | 0.00072 | 0.00257 | 0.01014 | 0.00893 | 0.00003 | 0.00039 |
| CN | −0.00215 | 0.03366 | 0.35100 | −0.40665 | 0.00078 | 0.00017 | 0.00009 | 0.00074 | 0.00262 | 0.01020 | 0.00888 | 0.00003 | 0.00038 |
| $COCH_3$ | −0.00248 | 0.03365 | 0.34895 | −0.40367 | 0.00068 | 0.00015 | 0.00009 | 0.00073 | 0.00268 | 0.01016 | 0.00839 | 0.00001 | 0.00038 |
| $COOC_2H_5$ | −0.00248 | 0.03357 | 0.34884 | −0.40349 | 0.00071 | 0.00016 | 0.00009 | 0.00073 | 0.00266 | 0.01009 | 0.00849 | 0.00002 | 0.00038 |
| COOH | −0.00241 | 0.03361 | 0.34950 | −0.40436 | 0.00072 | 0.00016 | 0.00009 | 0.00074 | 0.00266 | 0.01015 | 0.00852 | 0.00002 | 0.00038 |
| $C(CH_3)_3$ | −0.00317 | 0.03312 | 0.34726 | −0.40190 | 0.00071 | 0.00017 | 0.00008 | 0.00071 | 0.00256 | 0.00978 | 0.01003 | 0.00002 | 0.00038 |
| $CH_3CH_3$ | −0.00315 | 0.03320 | 0.34758 | −0.40159 | 0.00076 | 0.00018 | 0.00008 | 0.00071 | 0.00256 | 0.00982 | 0.00925 | 0.00003 | 0.00038 |
| F | −0.00282 | 0.03347 | 0.35022 | −0.40477 | 0.00088 | 0.00023 | 0.00010 | 0.00072 | 0.00257 | 0.01015 | 0.00861 | 0.00004 | 0.00038 |
| I | −0.00280 | 0.03346 | 0.34943 | −0.40432 | 0.00081 | 0.00020 | 0.00008 | 0.00072 | 0.00256 | 0.01006 | 0.00923 | 0.00003 | 0.00039 |
| $IO_2$ | −0.00221 | 0.03368 | 0.35173 | −0.40758 | 0.00084 | 0.00021 | 0.00008 | 0.00073 | 0.00262 | 0.01029 | 0.00897 | 0.00003 | 0.00039 |
| $CH_3$ | −0.00319 | 0.03318 | 0.34754 | −0.40145 | 0.00077 | 0.00018 | 0.00008 | 0.00071 | 0.00255 | 0.00980 | 0.00923 | 0.00003 | 0.00038 |
| $O(CH_2)_3CH_3$ | −0.00317 | 0.03328 | 0.34799 | −0.40168 | 0.00079 | 0.00018 | 0.00010 | 0.00072 | 0.00258 | 0.00995 | 0.00865 | 0.00002 | 0.00037 |
| $O(CH_3)_4CH_3$ | −0.00318 | 0.03328 | 0.34799 | −0.40167 | 0.00079 | 0.00018 | 0.00010 | 0.00072 | 0.00258 | 0.00995 | 0.00864 | 0.00002 | 0.00037 |
| $O(CH_2)_2CH_3$ | −0.00317 | 0.03329 | 0.34802 | −0.40172 | 0.00079 | 0.00018 | 0.00010 | 0.00072 | 0.00258 | 0.00995 | 0.00865 | 0.00002 | 0.00037 |
| $NH_2$ | −0.00353 | 0.03301 | 0.34741 | −0.40109 | 0.00078 | 0.00020 | 0.00009 | 0.00071 | 0.00253 | 0.00975 | 0.00954 | 0.00002 | 0.00038 |
| $NHCOCH_3$ | −0.00304 | 0.03319 | 0.34908 | −0.40382 | 0.00082 | 0.00019 | 0.00008 | 0.00071 | 0.00255 | 0.00986 | 0.00976 | 0.00001 | 0.00037 |
| $NO_2$ | −0.00216 | 0.03382 | 0.35202 | −0.40781 | 0.00079 | 0.00021 | 0.00010 | 0.00075 | 0.00268 | 0.01044 | 0.00850 | 0.00002 | 0.00038 |
| $OC_6H_5$ | −0.00300 | 0.03334 | 0.34872 | −0.40265 | 0.00080 | 0.00019 | 0.00010 | 0.00072 | 0.00257 | 0.00999 | 0.00860 | 0.00003 | 0.00038 |
| $OCH_2CH_3$ | −0.00317 | 0.03330 | 0.34802 | −0.40173 | 0.00079 | 0.00018 | 0.00010 | 0.00072 | 0.00258 | 0.00996 | 0.00864 | 0.00002 | 0.00037 |
| OH | −0.00319 | 0.03316 | 0.34899 | −0.40339 | 0.00088 | 0.00023 | 0.00009 | 0.00071 | 0.00251 | 0.00989 | 0.00950 | 0.00004 | 0.00038 |
| $OCH(CH_3)_2$ | −0.00329 | 0.03314 | 0.34759 | −0.40238 | 0.00078 | 0.00021 | 0.00009 | 0.00071 | 0.00252 | 0.00982 | 0.01019 | 0.00003 | 0.00038 |
| $OCH_3$ | −0.00321 | 0.03323 | 0.34794 | −0.40263 | 0.00081 | 0.00022 | 0.00009 | 0.00072 | 0.00253 | 0.00986 | 0.00985 | 0.00003 | 0.00038 |
| SH | −0.00286 | 0.03343 | 0.34925 | −0.40376 | 0.00079 | 0.00020 | 0.00008 | 0.00072 | 0.00257 | 0.01005 | 0.00896 | 0.00003 | 0.00039 |
| $Si(CH_3)_3$ | −0.00325 | 0.03316 | 0.34726 | −0.40152 | 0.00073 | 0.00016 | 0.00007 | 0.00071 | 0.00256 | 0.00982 | 0.00965 | 0.00002 | 0.00038 |
| $SCH_3$ | −0.00294 | 0.03337 | 0.34880 | −0.40317 | 0.00079 | 0.00020 | 0.00008 | 0.00072 | 0.00257 | 0.01001 | 0.00900 | 0.00003 | 0.00038 |
| $SOCH_3$ | −0.00265 | 0.03334 | 0.35064 | −0.40570 | 0.00089 | 0.00024 | 0.00008 | 0.00072 | 0.00257 | 0.01006 | 0.00924 | 0.00003 | 0.00039 |
| $SO_2CH_3$ | −0.00211 | 0.03374 | 0.35150 | −0.40708 | 0.00079 | 0.00021 | 0.00009 | 0.00074 | 0.00264 | 0.01035 | 0.00850 | 0.00002 | 0.00039 |
| $SO_2NH_2$ | −0.00212 | 0.03374 | 0.35148 | −0.40712 | 0.00080 | 0.00021 | 0.00009 | 0.00074 | 0.00264 | 0.01035 | 0.00855 | 0.00002 | 0.00039 |
| $CF_3$ | −0.00218 | 0.03368 | 0.35088 | −0.40660 | 0.00076 | 0.00017 | 0.00009 | 0.00074 | 0.00263 | 0.01023 | 0.00893 | 0.00003 | 0.00038 |
| $OCOCH_3$ | −0.00266 | 0.03345 | 0.35051 | −0.40544 | 0.00083 | 0.00020 | 0.00009 | 0.00072 | 0.00257 | 0.01015 | 0.00893 | 0.00003 | 0.00038 |
| $SCOCH_3$ | −0.00266 | 0.03351 | 0.35008 | −0.40510 | 0.00080 | 0.00020 | 0.00009 | 0.00072 | 0.00258 | 0.01014 | 0.00907 | 0.00003 | 0.00039 |

**Table S16.** All LRF-BO values, $\{\delta B^{O\text{-}H}/\delta v(L)\}_L^{All\ atoms}$, of para-substituted benzoic acids at the B3LYP/6-311G level. The numbering of the atoms listed in the top row is the same as presented in Figure 9 in the text.

| | O(1) | C(2) | O(3) | H(4) | C(5) | C(6) | C(7) | C(8) | C(9) | C(10) | H(11) | H(12) | H(14) | H(15) |
|---|---|---|---|---|---|---|---|---|---|---|---|---|---|---|
| H | −0.00325 | 0.03320 | 0.34799 | −0.40180 | 0.00081 | 0.00019 | 0.00008 | 0.00071 | 0.00255 | 0.00986 | 0.00904 | 0.00004 | 0.00038 | 0.00023 |
| $C_6H_5$ | −0.00309 | 0.03325 | 0.34774 | −0.40170 | 0.00082 | 0.00018 | 0.00008 | 0.00070 | 0.00259 | 0.00985 | 0.00898 | −0.00003 | 0.00034 | 0.00024 |
| Br | −0.00286 | 0.03338 | 0.34941 | −0.40395 | 0.00082 | 0.00018 | 0.00009 | 0.00073 | 0.00259 | 0.01003 | 0.00894 | −0.00004 | 0.00037 | 0.00026 |
| Cl | −0.00280 | 0.03341 | 0.34976 | −0.40447 | 0.00083 | 0.00018 | 0.00009 | 0.00073 | 0.00260 | 0.01007 | 0.00896 | −0.00003 | 0.00037 | 0.00027 |
| CN | −0.00241 | 0.03360 | 0.35066 | −0.40604 | 0.00081 | 0.00018 | 0.00010 | 0.00072 | 0.00261 | 0.01026 | 0.00889 | −0.00003 | 0.00037 | 0.00026 |
| $COCH_3$ | −0.00274 | 0.03344 | 0.34953 | −0.40434 | 0.00081 | 0.00017 | 0.00009 | 0.00071 | 0.00260 | 0.01011 | 0.00905 | −0.00002 | 0.00033 | 0.00025 |
| $CO_2C_2H_5$ | −0.00276 | 0.03339 | 0.34916 | −0.40369 | 0.00079 | 0.00018 | 0.00009 | 0.00072 | 0.00259 | 0.01007 | 0.00888 | −0.00001 | 0.00033 | 0.00025 |
| COOH | −0.00268 | 0.03346 | 0.34975 | −0.40451 | 0.00080 | 0.00018 | 0.00009 | 0.00072 | 0.00260 | 0.01015 | 0.00885 | −0.00001 | 0.00034 | 0.00025 |
| $C(CH_3)_3$ | −0.00330 | 0.03315 | 0.34734 | −0.40092 | 0.00082 | 0.00018 | 0.00008 | 0.00071 | 0.00258 | 0.00978 | 0.00897 | −0.00002 | 0.00032 | 0.00026 |
| $CH_2CH_3$ | −0.00326 | 0.03318 | 0.34743 | −0.40113 | 0.00082 | 0.00019 | 0.00008 | 0.00071 | 0.00258 | 0.00979 | 0.00900 | −0.00003 | 0.00036 | 0.00023 |
| F | −0.00294 | 0.03336 | 0.34941 | −0.40398 | 0.00084 | 0.00020 | 0.00009 | 0.00073 | 0.00259 | 0.01001 | 0.00903 | −0.00002 | 0.00035 | 0.00027 |
| I | −0.00289 | 0.03337 | 0.34926 | −0.40370 | 0.00082 | 0.00018 | 0.00009 | 0.00073 | 0.00259 | 0.01001 | 0.00890 | −0.00004 | 0.00038 | 0.00026 |
| $IO_2$ | −0.00245 | 0.03361 | 0.35136 | −0.40675 | 0.00079 | 0.00018 | 0.00010 | 0.00074 | 0.00260 | 0.01031 | 0.00888 | 0.00000 | 0.00037 | 0.00027 |
| $CH(CH_3)_2$ | −0.00325 | 0.03319 | 0.34741 | −0.40108 | 0.00081 | 0.00018 | 0.00007 | 0.00071 | 0.00258 | 0.00979 | 0.00902 | −0.00002 | 0.00035 | 0.00024 |
| $CH_3$ | −0.00326 | 0.03318 | 0.34740 | −0.40108 | 0.00082 | 0.00019 | 0.00008 | 0.00071 | 0.00257 | 0.00979 | 0.00900 | −0.00003 | 0.00036 | 0.00024 |
| SCN | −0.00269 | 0.03345 | 0.34989 | −0.40485 | 0.00084 | 0.00019 | 0.00009 | 0.00072 | 0.00261 | 0.01010 | 0.00900 | −0.00003 | 0.00036 | 0.00027 |
| $N(CH_3)_2$ | −0.00382 | 0.03298 | 0.34531 | −0.39823 | 0.00088 | 0.00019 | 0.00008 | 0.00070 | 0.00263 | 0.00950 | 0.00921 | −0.00003 | 0.00032 | 0.00023 |
| $NH_2$ | −0.00375 | 0.03299 | 0.34585 | −0.39893 | 0.00089 | 0.00020 | 0.00008 | 0.00070 | 0.00263 | 0.00954 | 0.00922 | −0.00002 | 0.00035 | 0.00023 |
| $NHCOCH_3$ | −0.00321 | 0.03332 | 0.34725 | −0.40124 | 0.00083 | 0.00018 | 0.00008 | 0.00071 | 0.00263 | 0.00984 | 0.00897 | −0.00001 | 0.00035 | 0.00026 |
| $NHCH_3$ | −0.00382 | 0.03293 | 0.34559 | −0.39847 | 0.00088 | 0.00021 | 0.00008 | 0.00070 | 0.00262 | 0.00949 | 0.00920 | −0.00002 | 0.00033 | 0.00024 |
| $NO_2$ | −0.00233 | 0.03367 | 0.35168 | −0.40736 | 0.00082 | 0.00019 | 0.00009 | 0.00074 | 0.00263 | 0.01040 | 0.00887 | 0.00000 | 0.00031 | 0.00028 |
| $OC_6H_5$ | −0.00332 | 0.03318 | 0.34750 | −0.40119 | 0.00085 | 0.00020 | 0.00008 | 0.00072 | 0.00260 | 0.00976 | 0.00900 | −0.00001 | 0.00034 | 0.00027 |
| $O(CH_2)_3CH_3$ | −0.00340 | 0.03315 | 0.34708 | −0.40084 | 0.00086 | 0.00019 | 0.00009 | 0.00071 | 0.00261 | 0.00972 | 0.00923 | −0.00003 | 0.00033 | 0.00023 |
| $OCOCH_3$ | −0.00307 | 0.03323 | 0.34854 | −0.40265 | 0.00085 | 0.00020 | 0.00009 | 0.00073 | 0.00260 | 0.00991 | 0.00899 | −0.00001 | 0.00031 | 0.00027 |
| $OCH_2CH_3$ | −0.00343 | 0.03313 | 0.34718 | −0.40068 | 0.00085 | 0.00020 | 0.00008 | 0.00072 | 0.00261 | 0.00972 | 0.00900 | −0.00001 | 0.00034 | 0.00026 |
| OH | −0.00327 | 0.03319 | 0.34777 | −0.40173 | 0.00087 | 0.00020 | 0.00008 | 0.00071 | 0.00260 | 0.00978 | 0.00919 | −0.00003 | 0.00034 | 0.00025 |
| $OCH(CH_3)_2$ | −0.00346 | 0.03312 | 0.34699 | −0.40041 | 0.00085 | 0.00020 | 0.00008 | 0.00072 | 0.00260 | 0.00970 | 0.00899 | −0.00001 | 0.00033 | 0.00026 |
| $OCH_3$ | −0.00336 | 0.03317 | 0.34724 | −0.40107 | 0.00086 | 0.00019 | 0.00009 | 0.00071 | 0.00261 | 0.00974 | 0.00924 | −0.00002 | 0.00033 | 0.00024 |
| $O(CH_3)_4CH_3$ | −0.00340 | 0.03315 | 0.34708 | −0.40082 | 0.00087 | 0.00019 | 0.00009 | 0.00071 | 0.00261 | 0.00971 | 0.00922 | −0.00003 | 0.00033 | 0.00023 |
| $O(CH_2)_2CH_3$ | −0.00342 | 0.03313 | 0.34714 | −0.40062 | 0.00085 | 0.00020 | 0.00008 | 0.00072 | 0.00260 | 0.00972 | 0.00898 | −0.00001 | 0.00034 | 0.00026 |
| $SCH(CH_3)_2$ | −0.00307 | 0.03328 | 0.34851 | −0.40261 | 0.00081 | 0.00018 | 0.00008 | 0.00072 | 0.00257 | 0.00992 | 0.00898 | −0.00004 | 0.00037 | 0.00024 |
| $SC_2H_5$ | −0.00304 | 0.03330 | 0.34865 | −0.40279 | 0.00081 | 0.00018 | 0.00008 | 0.00072 | 0.00257 | 0.00995 | 0.00894 | −0.00004 | 0.00037 | 0.00025 |
| SH | −0.00307 | 0.03329 | 0.34844 | −0.40259 | 0.00083 | 0.00018 | 0.00008 | 0.00071 | 0.00259 | 0.00989 | 0.00898 | −0.00003 | 0.00036 | 0.00026 |
| $Si(CH_2CH_3)_3$ | −0.00323 | 0.03321 | 0.34761 | −0.40135 | 0.00080 | 0.00018 | 0.00008 | 0.00071 | 0.00258 | 0.00984 | 0.00899 | −0.00003 | 0.00037 | 0.00022 |

**Table S16.** *Cont.*

| | O(1) | C(2) | O(3) | H(4) | C(5) | C(6) | C(7) | C(8) | C(9) | C(10) | H(11) | H(12) | H(14) | H(15) |
|---|---|---|---|---|---|---|---|---|---|---|---|---|---|---|
| $Si(CH_3)_3$ | −0.00324 | 0.03320 | 0.34762 | −0.40132 | 0.00080 | 0.00018 | 0.00008 | 0.00071 | 0.00256 | 0.00983 | 0.00899 | −0.00003 | 0.00037 | 0.00022 |
| $SCH_3$ | −0.00300 | 0.03332 | 0.34880 | −0.40302 | 0.00081 | 0.00018 | 0.00008 | 0.00072 | 0.00258 | 0.00997 | 0.00895 | −0.00004 | 0.00037 | 0.00025 |
| $SO_2CH_3$ | −0.00242 | 0.03360 | 0.35148 | −0.40696 | 0.00080 | 0.00018 | 0.00011 | 0.00074 | 0.00261 | 0.01033 | 0.00893 | 0.00000 | 0.00035 | 0.00027 |
| $SO_2NH_2$ | −0.00242 | 0.03360 | 0.35144 | −0.40695 | 0.00080 | 0.00018 | 0.00011 | 0.00074 | 0.00261 | 0.01034 | 0.00893 | 0.00000 | 0.00035 | 0.00027 |
| $SOCH_3$ | −0.00279 | 0.03338 | 0.35006 | −0.40476 | 0.00081 | 0.00018 | 0.00010 | 0.00073 | 0.00258 | 0.01010 | 0.00909 | −0.00001 | 0.00031 | 0.00024 |
| $CF_3$ | −0.00243 | 0.03358 | 0.35081 | −0.40618 | 0.00081 | 0.00018 | 0.00010 | 0.00073 | 0.00260 | 0.01027 | 0.00888 | −0.00002 | 0.00035 | 0.00026 |
| $SCOCH_3$ | −0.00300 | 0.03332 | 0.34864 | −0.40285 | 0.00080 | 0.00018 | 0.00008 | 0.00072 | 0.00258 | 0.00997 | 0.00894 | −0.00004 | 0.00037 | 0.00025 |

**Table S17.** All LRF-BO values, $\left\{\delta B^{O\text{-}H}/\delta v(L)\right\}_{L}^{All\ atoms}$, of meta-substituted benzoic acids at the B3LYP/6-311++G** level. The numbering of the atoms listed in the top row is the same as presented in Figure 9 in the text.

| | **O(1)** | **C(2)** | **O(3)** | **H(4)** | **C(5)** | **C(6)** | **C(7)** | **C(8)** | **C(9)** | **C(10)** | **H(11)** | **H(13)** | **H(15)** |
|---|---|---|---|---|---|---|---|---|---|---|---|---|---|
| H | −0.01529 | 0.04904 | 0.26933 | −0.29333 | 0.00125 | 0.00082 | 0.00124 | 0.00119 | 0.00205 | 0.00647 | −0.02633 | 0.00013 | 0.00135 |
| $C_6H_5$ | −0.01409 | 0.04894 | 0.26206 | −0.28745 | 0.00129 | 0.00080 | 0.00094 | 0.00128 | 0.00256 | 0.00909 | −0.02908 | 0.00000 | 0.00134 |
| Br | −0.01285 | 0.04901 | 0.26411 | −0.29400 | 0.00123 | 0.00116 | 0.00124 | 0.00125 | 0.00228 | 0.00749 | −0.02386 | 0.00030 | 0.00127 |
| Cl | −0.01342 | 0.04947 | 0.26401 | −0.29329 | 0.00148 | 0.00125 | 0.00126 | 0.00129 | 0.00225 | 0.00758 | −0.02414 | 0.00039 | 0.00111 |
| CN | −0.00989 | 0.04855 | 0.26424 | −0.29498 | 0.00095 | 0.00076 | 0.00091 | 0.00118 | 0.00257 | 0.00871 | −0.02647 | 0.00036 | 0.00129 |
| $COCH_3$ | −0.01172 | 0.04966 | 0.25613 | −0.28735 | 0.00101 | 0.00070 | 0.00090 | 0.00141 | 0.00287 | 0.01019 | −0.02741 | 0.00012 | 0.00132 |
| $COOC_2H_5$ | −0.01103 | 0.04868 | 0.26059 | −0.28828 | 0.00045 | 0.00077 | 0.00096 | 0.00128 | 0.00280 | 0.00916 | −0.02871 | 0.00001 | 0.00136 |
| COOH | −0.01206 | 0.04904 | 0.26074 | −0.28854 | 0.00086 | 0.00077 | 0.00098 | 0.00132 | 0.00276 | 0.00933 | −0.02894 | 0.00004 | 0.00147 |
| $C(CH_3)_3$ | −0.01324 | 0.04806 | 0.25991 | −0.28801 | 0.00174 | 0.00085 | 0.00095 | 0.00135 | 0.00265 | 0.00968 | −0.02823 | −0.00001 | 0.00112 |
| $CH_3CH_3$ | −0.01562 | 0.04885 | 0.25953 | −0.28837 | 0.00149 | 0.00086 | 0.00111 | 0.00136 | 0.00253 | 0.00848 | −0.02511 | 0.00041 | 0.00085 |
| F | −0.01393 | 0.04915 | 0.26417 | −0.29282 | 0.00145 | 0.00080 | 0.00122 | 0.00129 | 0.00224 | 0.00754 | −0.02409 | 0.00034 | 0.00130 |
| I | −0.01343 | 0.04953 | 0.26303 | −0.29299 | 0.00118 | 0.00123 | 0.00116 | 0.00125 | 0.00224 | 0.00748 | −0.02311 | 0.00048 | 0.00123 |
| $IO_2$ | −0.00972 | 0.04966 | 0.26565 | −0.29888 | 0.00098 | 0.00093 | 0.00108 | 0.00138 | 0.00260 | 0.00845 | −0.02472 | 0.00026 | 0.00125 |
| $CH_3$ | −0.01327 | 0.04847 | 0.26362 | −0.29078 | 0.00127 | 0.00083 | 0.00107 | 0.00125 | 0.00245 | 0.00810 | −0.02656 | 0.00022 | 0.00117 |
| $O(CH_2)_3CH_3$ | −0.01692 | 0.05037 | 0.25781 | −0.28561 | 0.00155 | 0.00074 | 0.00106 | 0.00140 | 0.00243 | 0.00878 | −0.02476 | 0.00020 | 0.00128 |
| $O(CH_3)_4CH_3$ | −0.01639 | 0.05034 | 0.25884 | −0.28681 | 0.00142 | 0.00072 | 0.00103 | 0.00138 | 0.00244 | 0.00881 | −0.02479 | 0.00015 | 0.00125 |
| $O(CH_2)_2CH_3$ | −0.01730 | 0.05037 | 0.25721 | −0.28506 | 0.00163 | 0.00075 | 0.00109 | 0.00141 | 0.00241 | 0.00875 | −0.02445 | 0.00026 | 0.00129 |
| $NH_2$ | −0.01739 | 0.04961 | 0.26150 | −0.28926 | 0.00204 | 0.00098 | 0.00122 | 0.00136 | 0.00221 | 0.00775 | −0.02329 | 0.00064 | 0.00080 |
| $NHCOCH_3$ | −0.01522 | 0.04917 | 0.25881 | −0.28841 | 0.00199 | 0.00089 | 0.00112 | 0.00147 | 0.00269 | 0.00971 | −0.02668 | 0.00026 | 0.00126 |
| $NO_2$ | −0.01036 | 0.04966 | 0.26406 | −0.29799 | 0.00113 | 0.00064 | 0.00115 | 0.00139 | 0.00272 | 0.00901 | −0.02285 | 0.00003 | 0.00113 |
| $OC_6H_5$ | −0.01481 | 0.04973 | 0.26029 | −0.29001 | 0.00123 | 0.00068 | 0.00110 | 0.00132 | 0.00247 | 0.00840 | −0.02355 | 0.00038 | 0.00080 |
| $OCH_2CH_3$ | −0.01545 | 0.04991 | 0.25804 | −0.28815 | 0.00154 | 0.00069 | 0.00102 | 0.00130 | 0.00239 | 0.00862 | −0.02256 | 0.00015 | 0.00081 |
| OH | −0.01540 | 0.04863 | 0.26394 | −0.29226 | 0.00201 | 0.00072 | 0.00124 | 0.00135 | 0.00222 | 0.00786 | −0.02365 | 0.00018 | 0.00132 |
| $OCH(CH_3)_2$ | −0.01450 | 0.04907 | 0.25148 | −0.28008 | 0.00122 | 0.00072 | 0.00108 | 0.00143 | 0.00260 | 0.00883 | −0.02368 | 0.00044 | 0.00103 |
| $OCH_3$ | −0.01385 | 0.04812 | 0.26665 | −0.29591 | 0.00145 | 0.00049 | 0.00109 | 0.00156 | 0.00272 | 0.00880 | −0.02519 | 0.00021 | 0.00115 |
| SH | −0.01191 | 0.04859 | 0.26772 | −0.29624 | 0.00139 | 0.00085 | 0.00093 | 0.00124 | 0.00230 | 0.00800 | −0.02564 | 0.00026 | 0.00123 |
| $Si(CH_3)_3$ | −0.01618 | 0.04873 | 0.26306 | −0.28552 | 0.00147 | 0.00137 | 0.00133 | 0.00150 | 0.00242 | 0.00731 | −0.02963 | 0.00060 | 0.00126 |
| $SCH_3$ | −0.01084 | 0.04670 | 0.27075 | −0.29805 | 0.00102 | 0.00082 | 0.00093 | 0.00149 | 0.00279 | 0.00792 | −0.02852 | 0.00043 | 0.00117 |
| $SOCH_3$ | −0.01341 | 0.04935 | 0.26280 | −0.29498 | 0.00212 | 0.00093 | 0.00101 | 0.00138 | 0.00250 | 0.00923 | −0.02471 | 0.00053 | 0.00090 |
| $SO_2CH_3$ | −0.01218 | 0.04968 | 0.26297 | −0.29458 | 0.00128 | 0.00081 | 0.00095 | 0.00142 | 0.00279 | 0.01056 | −0.02809 | 0.00023 | 0.00140 |
| $SO_2NH_2$ | −0.01342 | 0.04992 | 0.26098 | −0.29334 | 0.00151 | 0.00086 | 0.00112 | 0.00151 | 0.00274 | 0.01013 | −0.02567 | 0.00035 | 0.00132 |
| $CF_3$ | −0.01055 | 0.04912 | 0.26271 | −0.29437 | 0.00118 | 0.00071 | 0.00100 | 0.00131 | 0.00261 | 0.00915 | −0.02545 | 0.00019 | 0.00114 |
| $OCOCH_3$ | −0.01341 | 0.04978 | 0.26704 | −0.29431 | 0.00143 | 0.00070 | 0.00102 | 0.00128 | 0.00239 | 0.00844 | −0.02692 | 0.00045 | 0.00099 |
| $SCOCH_3$ | −0.01232 | 0.04890 | 0.26611 | −0.29588 | 0.00151 | 0.00107 | 0.00109 | 0.00135 | 0.00254 | 0.00876 | −0.02700 | 0.00049 | 0.00094 |

**Table S18.** All LRF-BO values, $\left\{\delta B^{O\text{-}H}/\delta v(L)\right\}_{L}^{All\ atoms}$, of para-substituted benzoic acids at the B3LYP/6-311++G** level. The numbering of the atoms listed in the top row is the same as presented in Figure 9 in the text.

| | O(1) | C(2) | O(3) | H(4) | C(5) | C(6) | C(7) | C(8) | C(9) | C(10) | H(11) | H(12) | H(14) | H(15) |
|---|---|---|---|---|---|---|---|---|---|---|---|---|---|---|
| H | −0.01529 | 0.04904 | 0.26933 | −0.29333 | 0.00125 | 0.00082 | 0.00124 | 0.00119 | 0.00205 | 0.00647 | −0.02633 | 0.00013 | 0.00135 | 0.00029 |
| $C_6H_5$ | −0.01398 | 0.04904 | 0.26368 | −0.28555 | 0.00160 | 0.00078 | 0.00090 | 0.00104 | 0.00220 | 0.00799 | −0.03089 | 0.00142 | 0.00088 | 0.00016 |
| Br | −0.01485 | 0.04913 | 0.26772 | −0.29428 | 0.00149 | 0.00082 | 0.00125 | 0.00117 | 0.00219 | 0.00688 | −0.02479 | 0.00123 | 0.00120 | 0.00027 |
| Cl | −0.01388 | 0.04859 | 0.26886 | −0.29520 | 0.00123 | 0.00080 | 0.00105 | 0.00111 | 0.00220 | 0.00678 | −0.02527 | 0.00158 | 0.00144 | 0.00022 |
| CN | −0.01059 | 0.04887 | 0.26866 | −0.29590 | 0.00118 | 0.00082 | 0.00084 | 0.00096 | 0.00221 | 0.00769 | −0.02728 | 0.00109 | 0.00116 | −0.00009 |
| $COCH_3$ | −0.01316 | 0.04907 | 0.26040 | −0.28541 | 0.00203 | 0.00073 | 0.00090 | 0.00091 | 0.00219 | 0.00925 | −0.03014 | 0.00103 | 0.00080 | 0.00034 |
| $CO_2C_2H_5$ | −0.01227 | 0.04887 | 0.26320 | −0.28773 | 0.00183 | 0.00087 | 0.00090 | 0.00093 | 0.00228 | 0.00894 | −0.03055 | 0.00118 | 0.00083 | 0.00026 |
| COOH | −0.01243 | 0.04915 | 0.26190 | −0.28735 | 0.00186 | 0.00086 | 0.00086 | 0.00096 | 0.00231 | 0.00902 | −0.03017 | 0.00124 | 0.00086 | 0.00038 |
| $C(CH_3)_3$ | −0.01348 | 0.04878 | 0.26044 | −0.28471 | 0.00200 | 0.00065 | 0.00095 | 0.00093 | 0.00225 | 0.00900 | −0.02904 | 0.00102 | 0.00082 | 0.00003 |
| $CH_2CH_3$ | −0.01447 | 0.04819 | 0.26742 | −0.29095 | 0.00169 | 0.00083 | 0.00103 | 0.00105 | 0.00226 | 0.00713 | −0.02654 | 0.00131 | 0.00101 | −0.00015 |
| F | −0.01497 | 0.04885 | 0.26991 | −0.29477 | 0.00124 | 0.00081 | 0.00120 | 0.00119 | 0.00218 | 0.00672 | −0.02600 | 0.00175 | 0.00130 | 0.00030 |
| I | −0.01444 | 0.04898 | 0.27013 | −0.29540 | 0.00113 | 0.00077 | 0.00129 | 0.00115 | 0.00212 | 0.00661 | −0.02602 | 0.00140 | 0.00153 | 0.00022 |
| $IO_2$ | −0.01111 | 0.04897 | 0.26898 | −0.29853 | 0.00127 | 0.00078 | 0.00107 | 0.00108 | 0.00222 | 0.00747 | −0.02511 | 0.00119 | 0.00123 | 0.00018 |
| $CH(CH_3)_2$ | −0.01426 | 0.04883 | 0.26534 | −0.28636 | 0.00174 | 0.00086 | 0.00105 | 0.00109 | 0.00228 | 0.00843 | −0.03190 | 0.00137 | 0.00112 | 0.00008 |
| $CH_3$ | −0.01454 | 0.04829 | 0.26953 | −0.29206 | 0.00147 | 0.00083 | 0.00107 | 0.00104 | 0.00217 | 0.00691 | −0.02744 | 0.00145 | 0.00095 | −0.00005 |
| SCN | −0.01330 | 0.04879 | 0.26070 | −0.28534 | 0.00191 | 0.00082 | 0.00090 | 0.00104 | 0.00224 | 0.00883 | −0.03033 | 0.00152 | 0.00102 | 0.00025 |
| $N(CH_3)_2$ | −0.01780 | 0.04884 | 0.26062 | −0.28143 | 0.00216 | 0.00080 | 0.00097 | 0.00130 | 0.00229 | 0.00837 | −0.02968 | 0.00091 | 0.00083 | 0.00034 |
| $NH_2$ | −0.01845 | 0.04886 | 0.26801 | −0.28956 | 0.00158 | 0.00083 | 0.00120 | 0.00133 | 0.00221 | 0.00653 | −0.02622 | 0.00150 | 0.00086 | 0.00035 |
| $NHCOCH_3$ | −0.01540 | 0.04906 | 0.26343 | −0.28658 | 0.00190 | 0.00080 | 0.00102 | 0.00120 | 0.00234 | 0.00835 | −0.02963 | 0.00098 | 0.00111 | 0.00034 |
| $NHCH_3$ | −0.01811 | 0.04876 | 0.26201 | −0.28198 | 0.00191 | 0.00088 | 0.00110 | 0.00137 | 0.00232 | 0.00786 | −0.03012 | 0.00156 | 0.00090 | 0.00043 |
| $NO_2$ | −0.01230 | 0.04964 | 0.26344 | −0.29400 | 0.00185 | 0.00083 | 0.00111 | 0.00103 | 0.00224 | 0.00840 | −0.02495 | 0.00099 | 0.00054 | 0.00032 |
| $OC_6H_5$ | −0.01534 | 0.04920 | 0.25724 | −0.28122 | 0.00207 | 0.00074 | 0.00094 | 0.00108 | 0.00230 | 0.00832 | −0.02848 | 0.00150 | 0.00088 | 0.00027 |
| $O(CH_2)_3CH_3$ | −0.01840 | 0.04891 | 0.26232 | −0.28433 | 0.00197 | 0.00075 | 0.00098 | 0.00115 | 0.00233 | 0.00789 | −0.02748 | 0.00129 | 0.00102 | 0.00031 |
| $OCOCH_3$ | −0.01429 | 0.04902 | 0.26765 | −0.29132 | 0.00152 | 0.00085 | 0.00093 | 0.00100 | 0.00215 | 0.00705 | −0.02715 | 0.00137 | 0.00102 | 0.00010 |
| $OCH_2CH_3$ | −0.01737 | 0.04845 | 0.25907 | −0.27947 | 0.00189 | 0.00081 | 0.00100 | 0.00113 | 0.00225 | 0.00789 | −0.02988 | 0.00169 | 0.00101 | 0.00056 |
| OH | −0.01709 | 0.04871 | 0.26785 | −0.29139 | 0.00166 | 0.00085 | 0.00118 | 0.00122 | 0.00223 | 0.00684 | −0.02586 | 0.00148 | 0.00083 | 0.00037 |
| $OCH(CH_3)_2$ | −0.01683 | 0.04880 | 0.25753 | −0.27912 | 0.00194 | 0.00076 | 0.00095 | 0.00111 | 0.00225 | 0.00798 | −0.02912 | 0.00159 | 0.00093 | 0.00052 |
| $OCH_3$ | −0.01532 | 0.04885 | 0.26786 | −0.29200 | 0.00175 | 0.00089 | 0.00095 | 0.00115 | 0.00239 | 0.00704 | −0.02656 | 0.00140 | 0.00068 | 0.00007 |
| $O(CH_3)_4CH_3$ | −0.01610 | 0.04839 | 0.26182 | −0.28352 | 0.00176 | 0.00074 | 0.00092 | 0.00110 | 0.00225 | 0.00755 | −0.02881 | 0.00156 | 0.00098 | 0.00070 |
| $O(CH_2)_2CH_3$ | −0.01845 | 0.04896 | 0.26247 | −0.28418 | 0.00199 | 0.00075 | 0.00101 | 0.00119 | 0.00232 | 0.00784 | −0.02767 | 0.00129 | 0.00102 | 0.00033 |
| $SCH(CH_3)_2$ | −0.01577 | 0.04902 | 0.26260 | −0.28464 | 0.00189 | 0.00076 | 0.00108 | 0.00122 | 0.00224 | 0.00851 | −0.03108 | 0.00110 | 0.00126 | 0.00036 |
| $SC_2H_5$ | −0.01658 | 0.04937 | 0.26297 | −0.28535 | 0.00204 | 0.00082 | 0.00112 | 0.00127 | 0.00223 | 0.00825 | −0.03007 | 0.00097 | 0.00126 | 0.00029 |
| SH | −0.01485 | 0.04862 | 0.27026 | −0.29479 | 0.00148 | 0.00080 | 0.00122 | 0.00113 | 0.00218 | 0.00690 | −0.02613 | 0.00122 | 0.00090 | 0.00023 |

**Table S18.** *Cont.*

| | O(1) | C(2) | O(3) | H(4) | C(5) | C(6) | C(7) | C(8) | C(9) | C(10) | H(11) | H(12) | H(14) | H(15) |
|---|---|---|---|---|---|---|---|---|---|---|---|---|---|---|
| $Si(CH_2CH_3)_3$ | −0.01223 | 0.04894 | 0.25971 | −0.28464 | 0.00181 | 0.00069 | 0.00096 | 0.00100 | 0.00226 | 0.00843 | −0.02949 | 0.00112 | 0.00094 | 0.00002 |
| $Si(CH_3)_3$ | −0.01365 | 0.04930 | 0.26097 | −0.28425 | 0.00184 | 0.00071 | 0.00098 | 0.00108 | 0.00220 | 0.00832 | −0.03025 | 0.00100 | 0.00120 | 0.00012 |
| $SCH_3$ | −0.01556 | 0.04900 | 0.26090 | −0.28234 | 0.00192 | 0.00085 | 0.00112 | 0.00114 | 0.00221 | 0.00830 | −0.03143 | 0.00125 | 0.00094 | 0.00051 |
| $SO_2CH_3$ | −0.01101 | 0.04925 | 0.26167 | −0.28973 | 0.00215 | 0.00088 | 0.00095 | 0.00100 | 0.00229 | 0.00975 | −0.02965 | 0.00097 | 0.00078 | 0.00029 |
| $SO_2NH_2$ | −0.01161 | 0.04920 | 0.26196 | −0.28966 | 0.00211 | 0.00084 | 0.00098 | 0.00104 | 0.00228 | 0.00929 | −0.02912 | 0.00098 | 0.00077 | 0.00025 |
| $SOCH_3$ | −0.01256 | 0.04924 | 0.26236 | −0.28736 | 0.00194 | 0.00076 | 0.00101 | 0.00104 | 0.00223 | 0.00909 | −0.03113 | 0.00128 | 0.00070 | 0.00034 |
| $CF_3$ | −0.01196 | 0.04943 | 0.26008 | −0.28663 | 0.00206 | 0.00084 | 0.00089 | 0.00099 | 0.00231 | 0.00957 | −0.03053 | 0.00114 | 0.00082 | 0.00027 |
| $SCOCH_3$ | −0.01281 | 0.04865 | 0.27003 | −0.29568 | 0.00147 | 0.00087 | 0.00105 | 0.00112 | 0.00226 | 0.00712 | −0.02666 | 0.00105 | 0.00122 | −0.00003 |

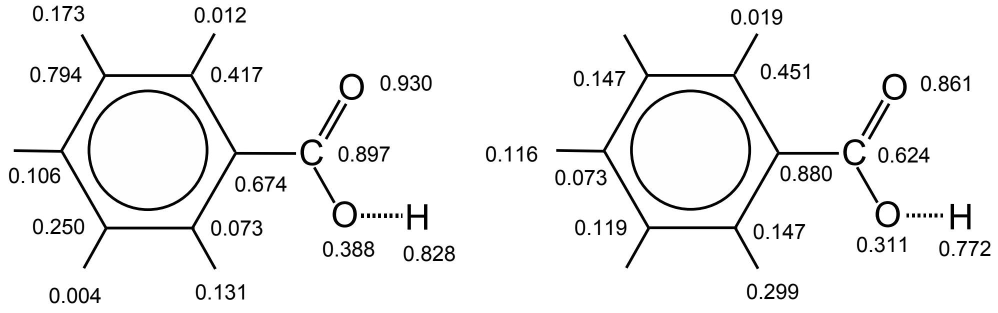


(**a**) (**b**)

**Figure S2.** The coefficients of determination for the correlations between the Hammett constants and the LRF-BO values, $\delta B^{O\text{-}H}/\delta v(L)$ with the virtual perturbation on each atomic site. (**a**) Meta-substituted benzoic acids; (**b**) Para-substituted benzoic acids.

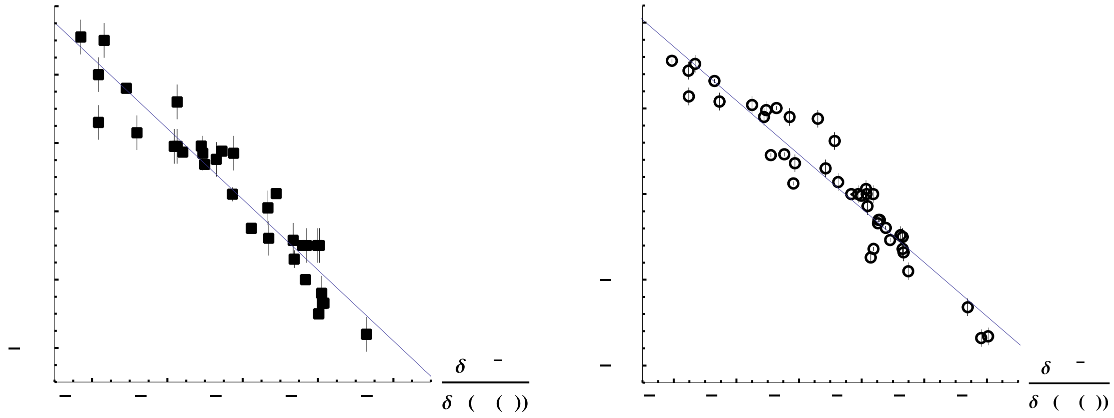

(**a**) (**b**)

**Figure S3.** Correlation between Hammett constants and $\delta B^{O\text{-}H}/\delta v(H(4))$ values at the B3LYP/6-31G level. (**a**) Meta-substituted benzoic acids; (**b**) Para-substituted benzoic acids. The coefficients of determination ($R^2$) are (**a**) 0.906 and (**b**) 0.917, respectively.

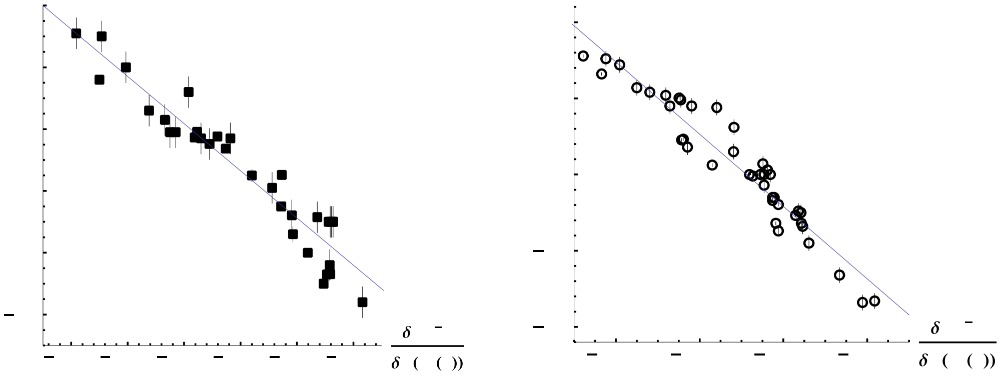

(**a**) (**b**)

**Figure S4.** Correlation between Hammett constants and $\delta B^{O\text{-}H}/\delta v(H(4))$ values at the B3LYP/6-31G** level. (**a**) meta-substituted benzoic acids; (**b**) para-substituted benzoic acids. The coefficients of determination ($R^2$) are (**a**) 0.912 and (**b**) 0.923, respectively.

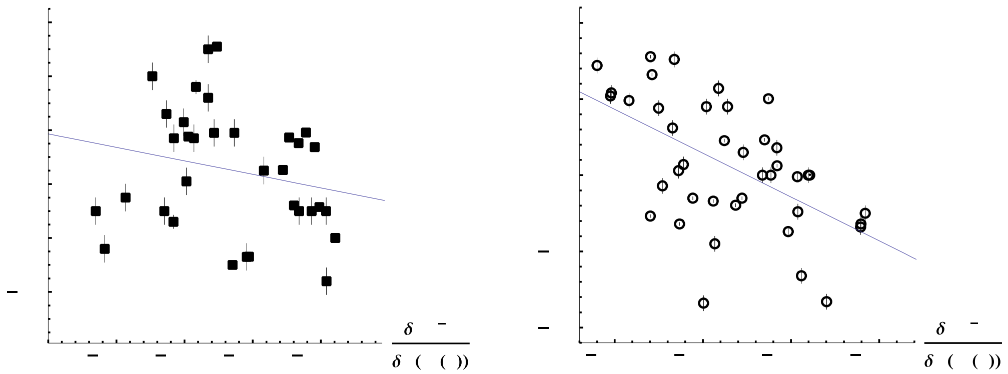

(**a**) (**b**)

**Figure S5.** Correlation between Hammett constants and $\delta B^{O\text{-}H}/\delta v(H(4))$ values at the B3LYP/ 6-31++G** level. (**a**) Meta-substituted benzoic acids; (**b**) Para-substituted benzoic acids. The coefficients of determination ($R^2$) are (**a**) 0.047 and (**b**) 0.309 respectively.

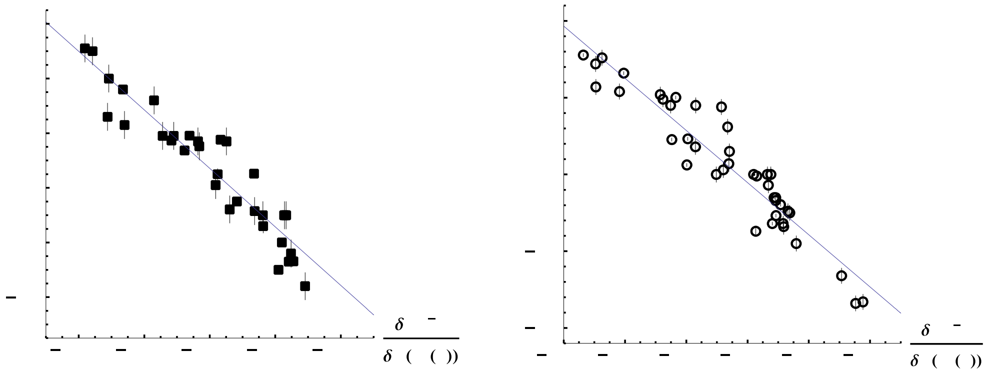

(**a**) (**b**)

**Figure S6.** Correlation between Hammett constants and $\delta B^{O\text{-}H}/\delta v(H(4))$ values at the B3LYP/ 6-311G level. (**a**) meta-substituted benzoic acids; (**b**) para-substituted benzoic acids. The coefficients of determination ($R^2$) are (**a**) 0.887 and (**b**) 0.895 respectively.

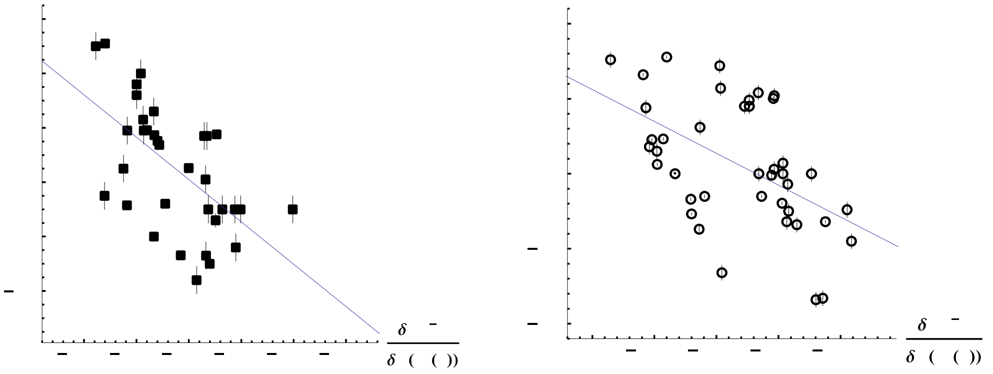

(**a**) (**b**)

**Figure S7.** Correlation between Hammett constants and $\delta B^{O\text{-}H}/\delta v(H(4))$ values at the B3LYP/ 6-311++G** level. (**a**) Meta-substituted benzoic acids; (**b**) Para-substituted benzoic acids. The coefficients of determination ($R^2$) are (**a**) 0.351 and (**b**) 0.268 respectively.

## References


1. Mayer, I. Charge, bond order and valence in the AB initio SCF theory. *Chem. Phys. Lett*. **1983**, *97*, 270–274.
2. Yamanaka, S.; Mitsuta, Y.; Okumura, M.; Yamaguchi, K.; Nakamura, H. Linear response function of the Mayer bond order: An indicator to describe intrinsic chemical reactivity of molecules. *Mol. Phys.* **2015**, *113*, 336–341.
3. Geerlings, P.; Fias, S.; Boisdenghien, Z.; de Proft, F. Conceptual DFT: Chemistry from the linear response function. *Chem. Soc. Rev.* **2014**, *43*, 4989—5008.
4. Matito, E.; Poater, J.; Solà, M.; Duran, M.; Salvador, P. Comparison of the AIM delocalization index and the Mayer and fuzzy atom bond orders. *J. Phys. Chem. A* **2005**, *109*, 9904–9910.
5. Fuentealba, P.; Cardenas, C.; Pino-Rios, R.; Tiznado, W. Applications of Toplogical Methods in Molecular Chemistry. In *Topological Analysis of Fukui Function*; Chauvin, R., Lepetit, C., Silvi, B., Alikhani, E., Eds.; Springer International Pulishing: Gewerbestrasse, Switzerland, 2016.
